# Acceptor engineering of metallacycles with high phototoxicity indices for safe and effective photodynamic therapy†

**DOI:** 10.1039/d2sc06936a

**Published:** 2023-02-08

**Authors:** Chonglu Li, Le Tu, Jingfang Yang, Chang Liu, Yuling Xu, Junrong Li, Wei Tuo, Bogdan Olenyuk, Yan Sun, Peter J. Stang, Yao Sun

**Affiliations:** a National Key Laboratory of Green Pesticide, International Joint Research Center for Intelligent Biosensor Technology and Health, College of Chemistry, Central China Normal University Wuhan 430079 China sunyaogbasp@ccnu.edu.cn; b Ministry of Education Key Laboratory for Special Functional Materials, Henan University Kaifeng 475004 China elaine.sun@utah.edu; c Department of Chemistry, University of Utah Salt Lake City Utah 84112 USA stang@chem.utah.edu; d Proteogenomics Research Institute for Systems Medicine 505 Coast Boulevard South La Jolla CA 92037 USA

## Abstract

Although metallacycle-based photosensitizers have attracted increasing attention in biomedicine, their clinical application has been hindered by their inherent dark toxicity and unsatisfactory phototherapeutic efficiency. Herein, we employ a π-expansion strategy for ruthenium acceptors to develop a series of Ru(ii) metallacycles (Ru1–Ru4), while simultaneously reducing dark toxicity and enhancing phototoxicity, thus obtaining a high phototoxicity index (PI). These metallacycles enable deep-tissue (∼7 mm) fluorescence imaging and reactive oxygen species (ROS) production and exhibit remarkable anti-tumor activity even under hypoxic conditions. Notably, Ru4 has the lowest dark toxicity, highest ROS generation ability and an optimal PI (∼146). Theoretical calculations verify that Ru4 exhibits the largest steric bulk and the lowest singlet–triplet energy gap (Δ*E*_ST_, 0.62 eV). *In vivo* studies confirm that Ru4 allows for effective and safe phototherapy against A549 tumors. This work thus is expected to open a new avenue for the design of high-performance metal-based photosensitizers for potential clinical applications.

## Abstract 2

Acceptor engineered supramolecular photosensitizers with a proming phototoxicity index (PI) for effective and safe photodynamic therapy *in vivo*.

## Graphical abstract

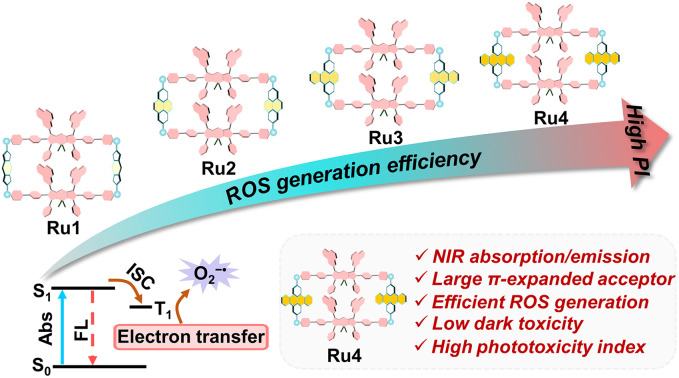

## Introduction

Photodynamic therapy (PDT) is a clinically approved therapeutic modality that has been extensively applied to treat cancer because of its temporal–spatial controllability and minimal side effects.^1–6^ The PDT mechanism relies on the conversion of an excited photosensitizer (PS) to molecular oxygen, under light irradiation, to generate ROS.^7–12^ To date, various advanced PSs have been developed, but several obstacles impede their acceptance in biomedicine.^13–16^ For example, PSs often undergo π–π stacking, which reduces the singlet oxygen quantum yield.^17,18^ Moreover, the inferior tissue penetration capability of the light sources currently used with PSs (<800 nm) reduces the PDT efficiency in deep-seated tumors.^19–21^ Dark cytotoxicity in PSs is undesired and avoiding it is critical for reducing treatment side effects.^22–24^ Thus, these challenges motivated us to develop novel photosensitizers for a safer and more effective PDT process.

Coordination-driven self-assembly involves the spontaneous formation of metallacycles with tunable sizes and shapes.^25–29^ Recently, the construction of metallacycle-based PSs has aroused continued interest in biomedicine.^30–33^ Metallacycle-based PSs demonstrate unique advantages that can effectively address issues encountered in traditional PS systems. For example, their rigid macrocyclic structure can effectively preserve ROS production through an anti-quenching effect.^34^ Furthermore, the presence of heavy atoms can improve PDT efficiency by promoting the intersystem crossing (ISC) process.^35^ By incorporating well-designed fluorescent ligands, the absorption/emission wavelengths have been recently shifted into the near-infrared (NIR) region, which can improve the diagnosis and phototherapy of deep-seated tumors.^36,37^ Despite these results, solving the inherent dark cytotoxicity of heavy metals remains largely unexplored, which greatly limited their translation from the laboratory to clinical use.

**Scheme 1:**
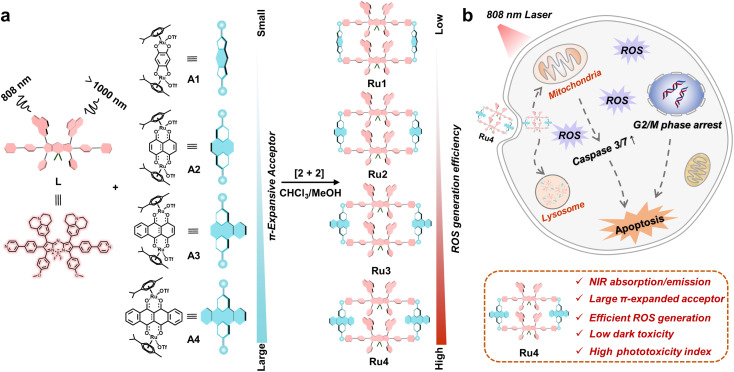
Schematic illustration of the design and antitumor mechanism of Ru1–Ru4. (a) The design, chemical structure and properties of Ru1–Ru4, which are excited at 808 nm and emitted over 1000 nm. (b) The underlying antitumor mechanism illustrates the internalization of Ru4 into A549 cells through the endocytic pathway and subsequently induces mitochondria-mediated apoptosis and cell cycle arrest in the G2/M phase.

Herein, we explore molecular engineering on Ru acceptors to develop highly efficient and NIR absorptive/emissive (800–1000 nm) Ru(ii) metallacycle-based PSs with low dark toxicity and high phototoxicity indices (PIs). A series of Ru(ii) metallacycles (Ru1–Ru4) were designed using the self-assembly of fluorescent ligands (L) and tunable π-expanded ruthenium acceptors (A1–A4) (Scheme 1). With the advantage of long-wavelength absorption/emission, Ru1–Ru4 were capable of deep-tissue fluorescence imaging and ROS production (∼7 mm) compared with visible-light-activated metallacycles (∼1 mm). Moreover, the *in vitro* anticancer results indicated that Ru1–Ru4 displayed high selectivity between A549 and 16HBE cells, and remarkable anticancer activity even under hypoxic conditions. Among the Ru(ii) metallacycles in this study, Ru4 had the largest steric bulk and highest electron density at the acceptor site. It demonstrated the highest PI (∼146) and ROS generation (∼20-fold compared to its ligand), which could be attributed to its smallest ligand bending angle (*θ*,149.4°), and the lowest singlet–triplet energy gap (Δ*E*_ST_, 0.62 eV) of the studied metallacycles. Therefore, Ru4 was selected to investigate the anticancer mechanism in detail. *In vivo* studies on PDT efficacy were then conducted, which demonstrate that Ru4 is a highly efficient PDT agent with low side effects.

## Results and discussion

### Design, synthesis and characterization of Ru(ii) metallacycles

Aza-boron-dipyrromethene (aza-BODIPY) was selected as the donor precursor owing to its high molar extinction coefficient and tunable emission wavelength.^38^ By introducing julolidine and anisole as strong electron-donating groups and phenylpyridine as the ruthenium coordinating group into the aza-BODIPY core, a fluorescent ligand (L) with NIR absorption/emission features was successfully synthesized (Scheme 1). As depicted in Fig. S1,† the energy bandgap in L was approximately 1.86 eV, which demonstrated that the emission wavelength of L was extended to the NIR-II range. Moreover, L also exhibited a low Δ*E*_ST_ value (1.23 eV). The structure of L was confirmed using nuclear magnetic resonance (NMR) spectroscopy and mass spectrometry (Fig. S2–4†).

**Fig. 1:**
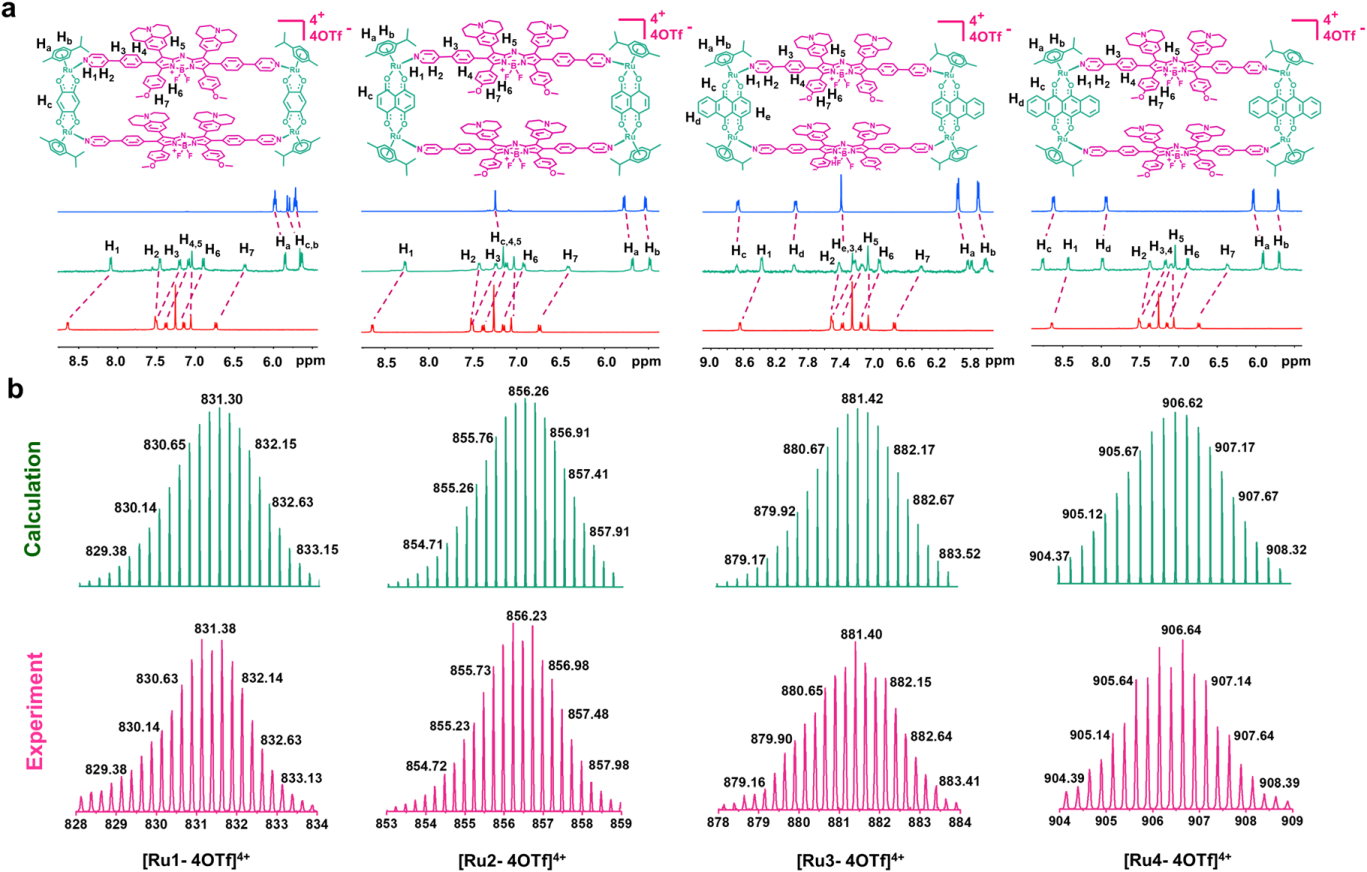
Chemical structure and characterization of Ru1–Ru4. (a) Chemical structures and partial ^1^H NMR spectra of Ru1–Ru4. (b) Experimental (magenta) and calculated (green) ESI-TOF-MS spectra of Ru1–Ru4.

Metallacycles Ru1–Ru4 were then constructed using the self-assembly of L with π-expansive ruthenium acceptors (A1–A4) (Scheme 1). Ru1–Ru4 were characterized using ^1^H-NMR spectroscopy, two-dimensional correlated spectroscopy (COSY), 2D-rotating frame Overhauser effect spectroscopy (ROESY) and electrospray ionization time-of-flight mass spectrometry (ESI-TOF-MS). The pyridyl proton H_1_ peak displayed an upfield shift (from 8.64 to 8.09 ppm for Ru1, 8.64 to 8.27 ppm for Ru2, 8.64 to 8.37 ppm for Ru3, and 8.64 to 8.43 ppm for Ru4) compared to the same peak in free L (Fig. 1a and S5–8†). The change in the chemical shift can be attributed to a loss of electron density when the ligand coordinated to the Ru(ii) acceptor. The ^1^H–^1^H COSY and ROESY spectra further supported the unambiguous assignment of the proton peaks in these metallacycles (Fig. S9–16†). ESI-TOF-MS spectra for Ru1–Ru4 were found to possess multiple prominent peaks for the assigned [2 + 2] assembly with charge states resulting from the loss of the triflate (OTf^−^) counterions (Fig. 1b and S17–20†). Peaks at *m*/*z* = 831.38, 856.23, 881.40, and 906.64 corresponded to [M − 4OTf]^4+^ for Ru1–Ru4, respectively. All the assigned peaks matched well with simulated theoretical distributions, indicating that Ru1–Ru4 possessed the expected 1 : 1 ratio of building blocks.

### Photophysical and photodynamic properties and stability studies

**Fig. 2:**
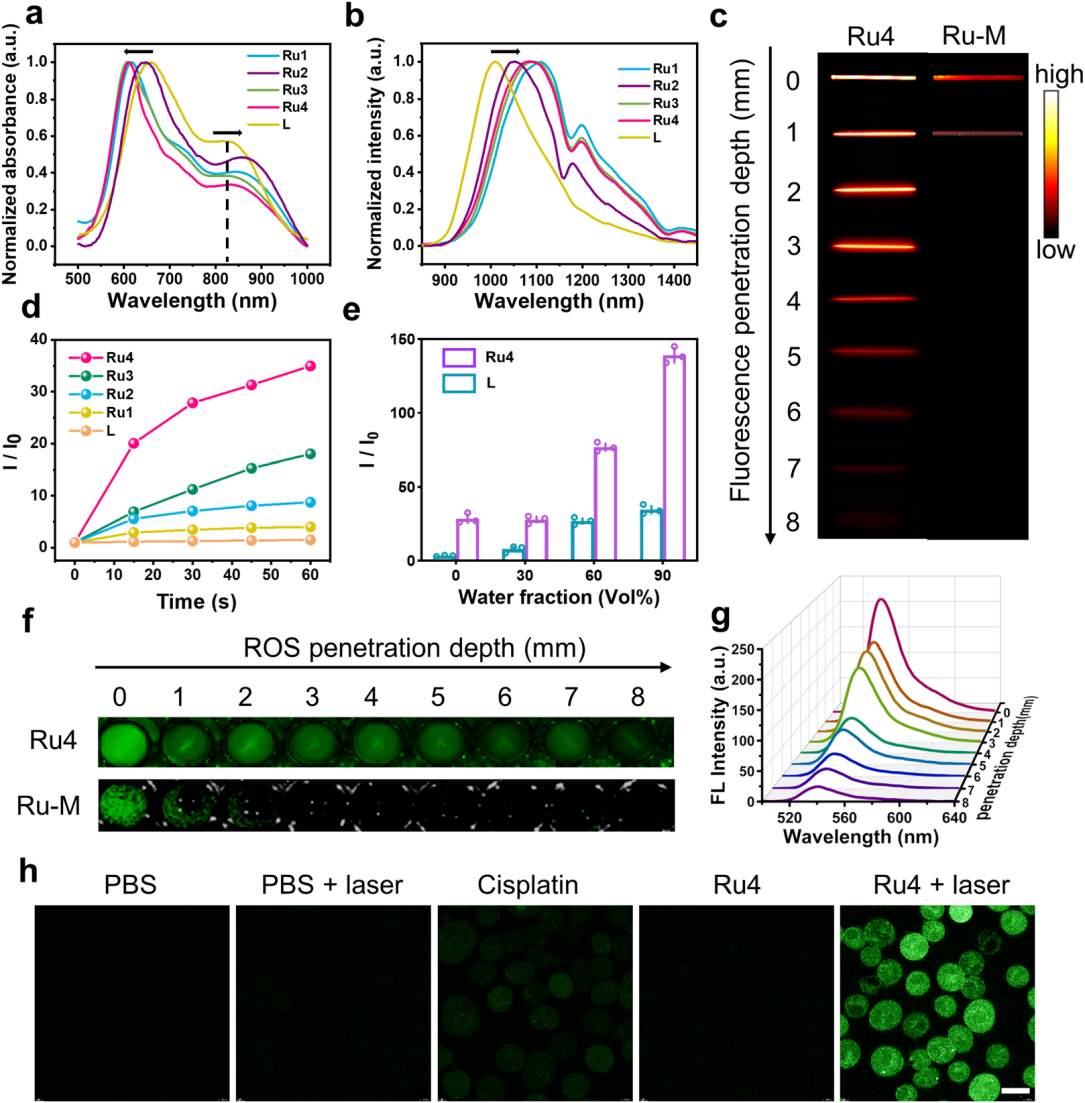
*In vitro* photophysical properties of Ru1–Ru4. (a) Normalized absorbance and (b) emission spectra (*λ*_ex_ = 808 nm) of Ru1–Ru4 in DCM. (c) Fluorescence images of Ru4 (10 μM) and Ru-M (10 μM) at various depths under 808 nm laser irradiation. (d) Comparison between the fluorescence intensity of DCFH-DA incubated with L (20 μM) and Ru1–Ru4 (10 μM), respectively. (e) Comparison between the ROS generation capacity of L (20 μM) and Ru4 (10 μM) with different *f*_w_. (f) The DCF fluorescence imaging of Ru4 (10 μM) and Ru-M (10 μM) at various depths. (g) Fluorescence spectra at different depths to monitor ROS generation by Ru4 under 808 nm laser irradiation. (h) Inverted fluorescence microscope images of A549 cells incubated with DCFH-DA after various treatments. Scale bar: 20 μm.

The ultraviolet-visible (UV-vis) absorption spectra of Ru1–Ru4 are shown in Fig. 2a. Free L exhibited two broad absorption bands with peaks at ∼650 and 830 nm. After L was coordinated with the Ru(ii) acceptors, and the absorption peak at 830 nm was red-shifted (24, 38, 7, and 15 nm for Ru1, Ru2, Ru3, and Ru4, respectively). The molar absorption coefficients (*ε*) of Ru1–Ru4 were 1.25 × 10^4^, 2.19 × 10^4^, 1.76 × 10^4^, and 1.70 × 10^4^ M^−1^ cm^−1^, respectively (Fig. S21†). Furthermore, the maximum emission wavelengths of L and Ru1–Ru4 in dichloromethane were 1008, 1108, 1050, 1093, and 1085 nm, respectively (Fig. 2b). The bathochromic shift of the absorption and emission wavelengths for Ru1–Ru4 could be attributed to the N–Ru coordination bond formation, which facilitated intramolecular charge transfer (ICT) in L. The chemical stability and photostability of Ru1–Ru4 were assessed by monitoring changes in the absorption spectra. As depicted in Fig. S22–24,†Ru1–Ru4 exhibited high chemical stability in the medium and a high photostability under continuous laser irradiation. Tissue-mimicking materials (1% intralipid) were used to assess the deep optical penetration ability of Ru1–Ru4, with a previously reported visible-light absorption/emission counterpart (Ru-M, Ex: 450 nm)^39^ used as the control. The fluorescence signals from Ru1–Ru4 could be observed at a penetration depth of 7 mm owing to the long absorption/emission wavelengths of Ru1–Ru4. Conversely, the FL signal of Ru-M became almost imperceptible when the penetration depth increases to 1 mm (Fig. 2c and S25†).

To investigate the effect of different π-expanded Ru(ii) acceptors on the PDT potential of the metallacycles, 2′,7′-dichlorodihydrofluorescein diacetate (DCFH-DA) was used to evaluate their ROS-production abilities.^40^L or Ru1–Ru4 were co-incubated with DCFH-DA and irradiated at 488 nm for 60 s. The fluorescence intensity at 525 nm increased significantly with the irradiation time. Notably, the DCF fluorescence intensity increased 31-fold after 60 s of laser irradiation in the presence of Ru4, which was higher than that of L (1.5-fold) or other Ru(ii) metallacycles (4-fold for Ru1, 9-fold for Ru2, and 18-fold for Ru3) (Fig. 2d and S26†). Additionally, 1,3-diphenylisobenzofuran (DPBF), hydroxyphenyl fluorescein (HPF) and dihydroethidium (DHE) were used to detect ^1^O_2_, hydroxyl radicals (OH˙), and superoxide anions (O_2_^−^˙), respectively. Under laser irradiation, the absorbance of DPBF at 416 nm and the fluorescence intensity of HPF at 516 nm remained relatively unchanged, while the fluorescence intensity of DHE at 595 nm significantly increased (Fig. S27–29†). Among them, the increase in O_2_^−^˙ generated by Ru4 (3.0-fold) exceeded that of Ru1 (1.2-fold), Ru2 (1.5-fold), and Ru3 (2.6-fold). To explore the anti-quenching ROS ability of the Ru(ii) metallacycles, DCFH-DA was incubated with L or Ru4, and the emission spectra were recorded in dimethyl sulfoxide/water. When the water fraction (*f*_w_) was increased from 0% to 90%, the fluorescence intensity of DCF induced by Ru4 increased rapidly compared with the intensity observed in L and DCFH-DA solutions (Fig. 2e and S30†). To evaluate whether the metallacycles can efficiently generate ROS in deep tissue, DCFH-DA incubated with either Ru4 or Ru-M was placed under 1% intralipid and exposed to laser irradiation. As shown in Fig. 2f, g and S31,†Ru4 efficiently generated ROS, even when the depth increased to 7 mm. In comparison, Ru-M cannot generate ROS beyond depths of 1 mm. Subsequently, we evaluated the capacity of Ru4 to generate ROS at the cellular level. After co-incubation with DCFH-DA, A549 cells treated with Ru4 and exposed to 808 nm laser irradiation displayed strong green fluorescence (Fig. 2h). A549 cells co-incubated with Ru4 and DHE exhibited typical red fluorescence under laser irradiation, indicating that DHE was exposed to O_2_^−^˙ (Fig. S32†). These results suggested that Ru4, which was a sterically bulky and electron-rich acceptor, can efficiently generate ROS.

### *In vitro* anticancer activity

Based on the promising photophysical properties, the anticancer efficacies of Ru1–Ru4 were evaluated using 3-[4,5-dimethylthiazol-2-yl]-2,5 diphenyl tetrazolium bromide (MTT) assays of A549, cisplatin-resistant A549 (A549/DDP), and 16HBE cells. The cells were co-incubated with different concentrations of Ru1–Ru4, A1–A4, and L and further treated with or without light, and the results are summarized in ESI Tables 1 and 2.† Under dark conditions, with the exception of Ru2, the metallacycles were inactive against A549 cells. Ru4 exhibited the lowest cytotoxicity (IC_50_ = 305.6 μM), which could be attributed to the steric bulk and the electron-rich acceptor properties preventing exchange with biomolecules.^41–43^ In contrast, under light irradiation, Ru4 was the most effective compound for the eradication of A549 cells (IC_50_ = 3.6 μM). Ru1–Ru4 exhibited good anticancer activity against cisplatin-resistant A549 cells and maintained high phototoxicity even under hypoxic conditions. Specifically, the PI of Ru4 can reach as high as 146. In addition, Ru4 demonstrated good selectivity for A549 cells with a calculated selectivity index (SI) of 2.7, which was higher than that of cisplatin (0.4), and Ru1–Ru3 (1.4, 1.0, and 0.9). These results indicated that Ru4 was superior to cisplatin and other metallacycles in maintaining excellent antitumor effects, being selective between A549 and 16HBE cells and possessing a high PI.

### Theoretical calculations of metallacycles

**Fig. 3:**
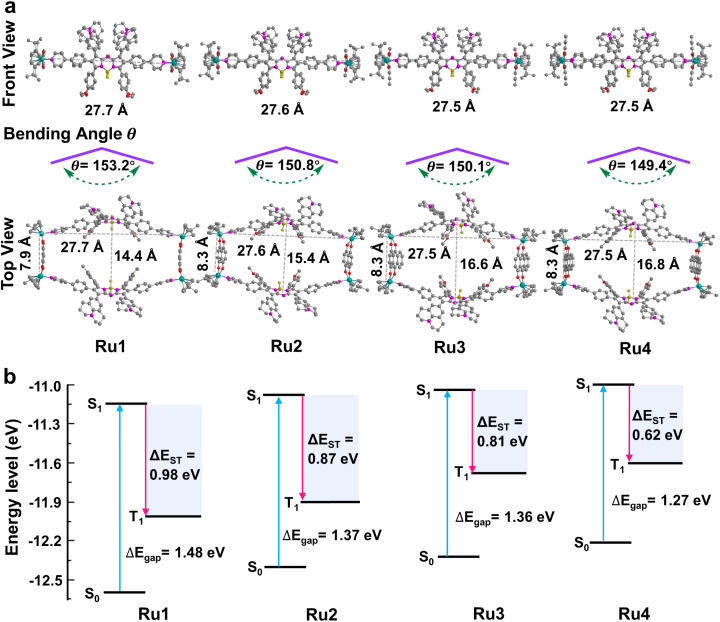
Theoretical calculations of Ru1–Ru4. (a) Optimized structure of metallacycles and the bending angle *θ* of L in metallacycles; hydrogen atoms in the structure are omitted for clarity. (b) Calculated HOMO and LUMO of Ru1–Ru4 at the B3LYP/6-31G(d) level. Excitation energy of the excited triplet state was determined under the B3LYP/6-31G(d) level by time-dependent DFT. Δ*E*_gap_ and Δ*E*_ST_ between singlet and triplet states are also presented in the figure.

These promising results inspired us to explore the relationship between the macrocycle structure and the photophysical properties using theoretical calculations. The geometric conformations of Ru1–Ru4 were optimized using density functional theory (DFT) with a 6-31G(d) basis set in Gaussian 16.^44–46^ The rectangular structure in Ru4 (27.5 × 16.8 Å cavity) was larger than that in Ru1 (27.7 × 14.4 Å), Ru2 (27.6 × 15.4 Å), and Ru3 (27.5 × 16.6 Å) (Fig. 3a). The bending angle *θ* (dihedral angle ∠N1–N2–B1–N3 that represents the angle between the phenylpyridine modified groups on both sides of the aza-BODIPY core) decreased in the order of free L (176.0°) > Ru1 (153.2°) > Ru2 (150.8°) > Ru3 (150.1°) > Ru4 (149.4°), which was expected, given the large cavity in the structure of Ru4 (Fig. 3a and S33†). These results suggested that the increase in the degree of π-expansion of the Ru(ii) acceptors enhanced the degree of twist in L, which improved the ICT effect and generation of ROS.^47^ The electronically excited structures and related photophysical properties were computed using a TD-DFT method. As shown in Fig. 3b and Table S3,† the HOMO–LUMO energy gaps of L, Ru1, Ru2, Ru3, and Ru4 were determined to be 1.86, 1.48, 1.37, 1.36, and 1.27 eV, respectively. Ru1–Ru4 exhibited a lower Δ*E*_gap_ than free L owing to the introduction of Ru-pyridyl coordination bonds and the electron-deficient nature of the transition metal. Δ*E*_ST_ was also investigated to evaluate the ROS generation efficiency. As shown in Fig. 3b, Ru4 has the smallest Δ*E*_ST_ (0.62 eV) of the Ru(ii) metallacycles, suggesting that the sterically bulky and electron-rich Ru(ii) acceptor can enhance the ISC of Ru4 and improve the efficiency of ROS production. The theoretical calculations were in good agreement with the experimental data and the results supported our design concept that efficient ROS generation can be achieved by engineering the acceptor in Ru(ii) metallacycles.

### Evaluation of cell uptake and localization of Ru4

**Fig. 4:**
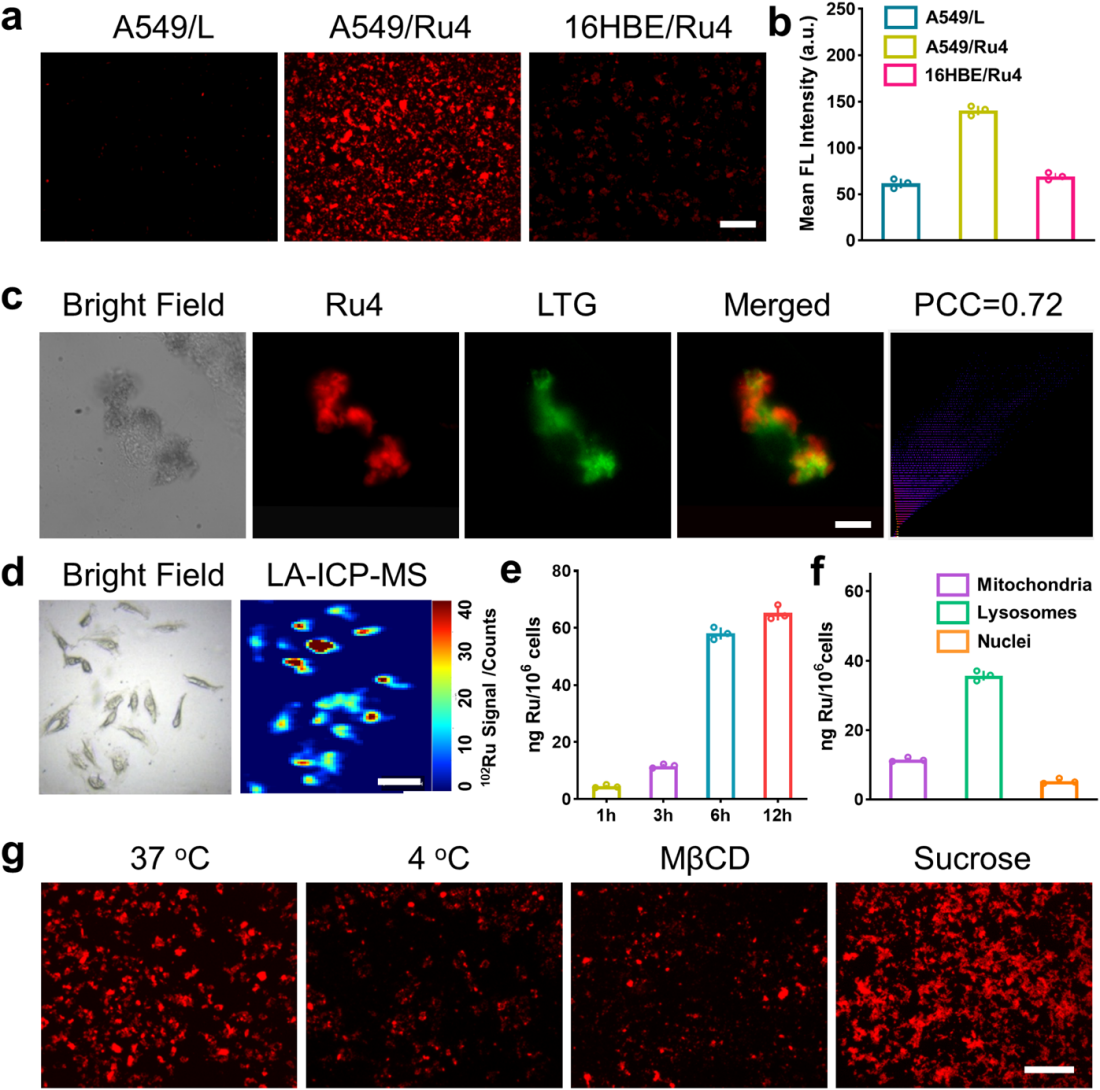
Cellular uptake and localization of Ru4 in A549 cells. (a) Fluorescence images of cells treated with Ru4 (10 μM) and L (20 μM), respectively. Scale bar: 300 μm. (b) Semi-quantitative analysis of the fluorescence imaging in Fig. 4a. (c) Colocalization assay of Ru4 (10 μM) using LTG. Scale bar: 10 μm. (d) LA-ICP-MS imaging of ^102^Ru in A549 cells incubated with Ru4 (10 μM). Scale bar: 50 μm. (e) ICP-MS results showing the Ru content in A549 cells over time. (f) Subcellular distribution of Ru concentration in A549 cells measured using ICP-MS. (g) Cellular uptake mechanism studies of Ru4 (10 μM) at 4 °C and 37 °C, MβCD and sucrose, respectively. Scale bar: 300 μm.

We firstly evaluated the octanol/water partition coefficient (log *P*_o/w_) of Ru1–Ru4 and L.^48^ As shown in Table S4,† all these compounds demonstrated similar log *P*. The cellular uptake and localization of Ru4 were further investigated using A549 cells. After incubation with Ru4, the cells showed a strong red fluorescence signal and the corresponding mean fluorescence intensity increased with the incubation time (Fig. S34†). Moreover, Ru4 demonstrated a much stronger fluorescence signal than L in A549 cells as well as in normal 16HBE cells (Fig. 4a, b and S34†). These results could be attributed to the unique shape and positive charge of the metallacycles, which are beneficial for cell uptake and selectivity towards A549 cells. An intracellular localization test was performed and, after 9 hours of incubation, the red fluorescence signal of Ru4 and green fluorescence from LysoTracker® Green (LTG) overlapped, showing a Pearson correlation coefficient (PCC) of 0.72 (Fig. 4c and S35 and 36†). Laser ablation inductively coupled plasma MS (LA-ICP-MS) for the A549 cells also showed a strong signal corresponding to ^102^Ru (Fig. 4d). Moreover, ICP-MS verified that Ru4 uptake increased with incubation time and reached 65.2 ng per million cells at 12 h (Fig. 4e). The calculated Ru content in the lysosomes was 35.8 ng per million cells, which was higher than that observed in the mitochondria and the nuclei (Fig. 4f). To investigate the cellular uptake mechanism of Ru4, cellular fluorescence imaging was done at low temperatures or with various metabolic and endocytic inhibitors. The intracellular red fluorescence of Ru4 dropped significantly at 4 °C or with metabolic inhibitors, suggesting that Ru4 was internalized into the cell through energy-dependent processes (Fig. 4g and S37†). Pretreatment with methyl-β-cyclodextrin (Mβ-CD) resulted in a decrease in the intracellular red fluorescence intensity, indicating that Ru4 was internalized into cancer cells *via* the energy-dependent caveolae-mediated endocytosis pathway.

### Cell death mechanisms

**Fig. 5:**
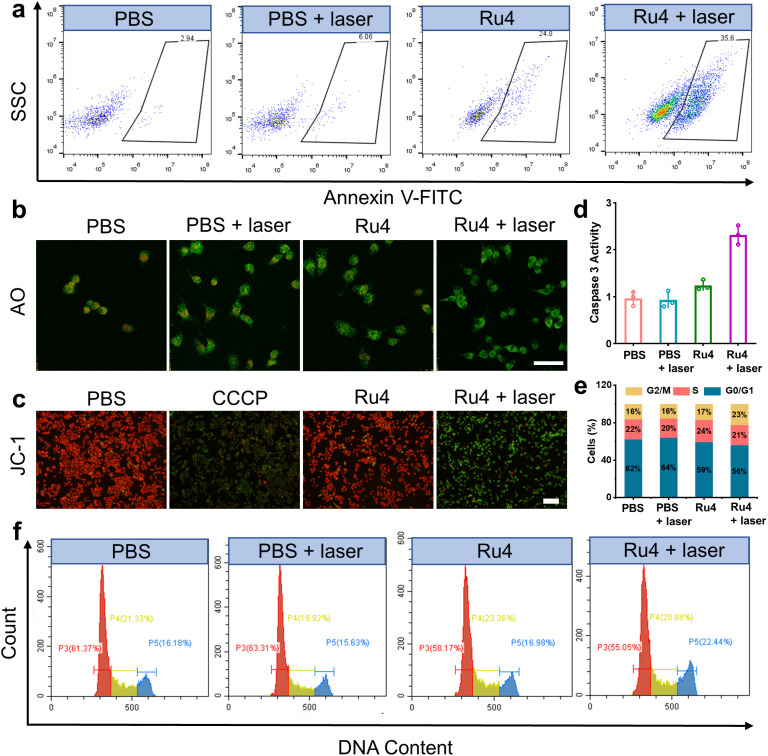
Cell death mechanisms of Ru4 in A549 cells.(a) Flow cytometry measuring cell apoptosis of A549 cells with PBS, PBS + laser, Ru4 (10 μM) and Ru4 (10 μM) + laser. (b) Confocal fluorescence imaging of AO stained A549 cells after different treatments. Scale bar: 20 μm. (c) Fluorescence imaging of JC-1 labelled A549 cells treated with PBS, CCCP (10 μM), Ru4 (10 μM), and Ru4 (10 μM) + laser. (d) Caspase 3 activity in A549 cells after various treatments. (e) Histogram depicting cell cycle distribution after various treatments. (f) Flow cytometry analysis of the cell cycle of A549 cells after various treatments.

Based on the excellent anticancer activity of Ru4, we investigated the mechanisms of cell death. A549 cells treated with Ru4 under 808 nm laser irradiation showed 35.6% apoptosis, indicating the enhanced cytotoxicity of Ru4 (Fig. 5a). To visualize the effect of Ru4 on cell death, co-staining of calcein AM and propidium iodide was performed to distinguish between live (green) and dead (red) cells.^49^ As for Ru4 or laser irradiation alone, strong green fluorescence from calcein AM and weak red fluorescence from propidium iodide were observed (Fig. S38†). In comparison, the combination of Ru4 and the laser together results in a strong propidium iodide signal, indicating the high ROS efficacy of Ru4. As Ru4 mainly accumulated in lysosomes, acridine orange (AO) was used to evaluate lysosomal membrane integrity.^50^ As shown in Fig. 5b and S39,† when A549 cells were incubated with Ru4 and subjected to laser irradiation, the red fluorescence produced by AO largely disappeared. Ru4 is also partially localized in the mitochondria, and JC-1 dye was used to evaluate the mitochondrial membrane potential (MMP) after PDT. Compared with the control groups, the Ru4-treated group under 808 nm laser irradiation showed intense green fluorescence (Fig. 5c and S40†). Mitochondrial damage often triggers downstream activation of the caspase cascade and apoptosis. As shown in Fig. 5d, the Ru4-treated group under laser irradiation showed a 2.2-fold increase in caspase 3 activity as compared to that in the Ru4 alone group. Subsequently, flow cytometry results indicated that the number of cells accumulated in the G2/M phase increased from 16.98% to 22.44% upon treatment with Ru4 and laser irradiation. Conversely, the number of cells in the S phase hardly increased (Fig. 5e and f). These results demonstrated that Ru4 under laser irradiation induced cell cycle arrests in the G2/M phase under laser irradiation.

### *In vivo* anticancer application

**Fig. 6:**
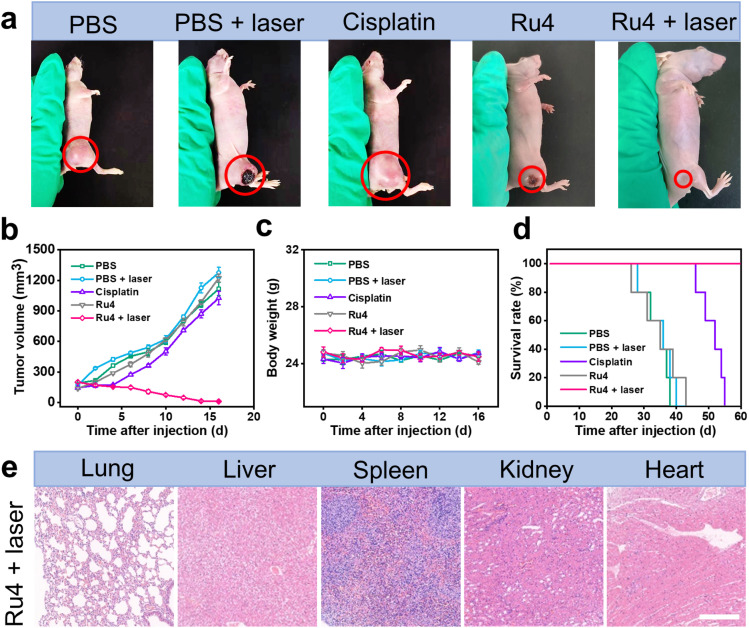
*In vivo* PDT effect of Ru4 in A549 tumor-bearing mice. (a) Representative photographs of A549 tumors treated with PBS, PBS + laser, cisplatin (10 μM), Ru4 (10 μM), and Ru4 (10 μM) + laser (808 nm, 1 W cm^−2^). (b) Tumor growth inhibition curves. (c) Body weight change curves of mice after various treatments. (d) Survival rate of the A549 tumor-bearing mice after different treatments. (e) Histological examination of the primary organs of mice treated with Ru4 (10 μM) + laser (808 nm, 1 W cm^−2^). Scale bar: 50 μm.

The outstanding *in vitro* results of Ru4 prompted us to further explore the feasibility of anti-tumor activity in the A549 tumor-bearing nude mice. When the xenograft tumor volumes reached around 100 mm^3^, the mice were randomly divided into five groups: (i) physiological saline (group 1), (ii) physiological saline and laser irradiation (group 2), (iii) cisplatin (group 3), (iv) Ru4-injected only (group 4), and (v) Ru4-injected and laser irradiation (group 5). For group 5, 12 hours after intratumoral injection of metallacycle Ru4 (1 mg Ru per kg body weight), the A549 tumor was irradiated with a laser (808 nm, 1 W cm^−2^, and 10 min). The same laser dose and the same Ru4 dose were used for group 2 and group 3. The tumor volumes and the body weight of the mice were recorded at 2 day intervals for a total of 16 days. As shown in Fig. 6a, b and S41,† the tumor volume of the Ru4 group treated with the laser reduced to 95% of the original volume on the 16 day, while the tumor volume of the mice treated with cisplatin and only Ru4 increased 7-fold and 9-fold respectively in the same period. Importantly, no significant weight loss was observed during the treatment (Fig. 6c), indicating that Ru4 has minimal side effects upon treatment. Moreover, the tumors treated with the Ru4 + laser group can effectively prolong the survival time of mice compared with the other tested groups (Fig. 6d). In addition, histological examination of the major organs (lung, liver, spleen, kidney and heart) of mice by hematoxylin and eosin (H&E) staining showed no obvious organ damage (Fig. 6e and S42†). Overall, Ru4 is effective for the treatment of cancer by PDT *in vivo*, and has no side effects.

## Conclusions

In conclusion, we applied molecular engineering to Ru(ii) acceptors in metallacycles to develop a series of metallacycle-based PSs. Among them, Ru4 exhibited the lowest dark toxicity, strongest ROS generation ability, highest PI, and excellent deep-tissue fluorescence and ROS penetration. Theoretical calculations verified that introducing sterically bulky and electron-rich moieties at the Ru(ii) acceptor position can reduce the bending angle of the ligand and Δ*E*_ST_ of the metallacycles. In addition, we also studied the antitumor mechanism *in vitro* and antitumor effect *in vivo* of Ru4 thoroughly. Therefore, this study provides a convenient and important strategy for developing high-efficiency metallacycle-based photosensitizers for potential clinical applications.

## Data availability

All the data supporting this article have been included in the main text and the ESI.†

## Author contributions

Yao Sun and P. J. S. conceived the project and designed the experiments. C. L., L. T. and Chang Liu designed, synthesized and characterized the materials. J. Y. and W. T. carried out the theoretical calculation. C. L., L. T. and Y. X. performed the *in vitro* and *in vivo* studies. Yao Sun, J. L., Yan Sun and P. J. S. wrote the manuscript. All authors analyzed and discussed the results and have given approval to the final version of the manuscript.

## Conflicts of interest

There are no conflicts to declare.

## Supplementary Material

SC-014-D2SC06936A-s001

## References

[cit1] Li X., Lovell J. F., Yoon J., Chen X. (2020). Nat. Rev. Clin. Oncol..

[cit2] Xie J., Wang Y., Choi W., Jangili P., Ge Y., Xu Y., Kang J., Liu L., Zhang B., Xie Z., He J., Xie N., Nie G., Zhang H., Kim J. S. (2021). Chem. Soc. Rev..

[cit3] Yao Q., Fan J., Long S., Zhao X., Li H., Du J., Shao K., Peng X. (2022). Chem.

[cit4] Zhu W., Li Y., Guo S., Guo W., Peng T., Li H., Liu B., Peng H., Tang B. (2022). Nat. Commun..

[cit5] McFarland S. A., Mandel A., Dumoulin-White R., Gasser G. (2020). Curr. Opin. Chem. Biol..

[cit6] Liu Z., Wang Q., Qiu W., Lyu Y., Zhu Z., Zhao X., Zhu W. (2022). Chem. Sci..

[cit7] Chakrabortty S., Agrawalla B. K., Stumper A., Vegi N. M., Fischer S., Reichardt C., Kogler M., Dietzek B., Feuring-Buske M., Buske C., Rau S., Weil T. (2017). J. Am. Chem. Soc..

[cit8] Yang B., Chen Y., Shi J. (2019). Chem. Rev..

[cit9] Lan G., Ni K., Xu Z., Veroneau S. S., Song Y., Lin W. (2018). J. Am. Chem. Soc..

[cit10] Wu W., Mao D., Xu S., Kenry, Hu F., Li X., Kong D., Liu B. (2018). Chem.

[cit11] Liu M., Chen Y., Guo Y., Yuan H., Cui T., Yao S., Jin S., Fan H., Wang C., Xie R., He W., Guo Z. (2022). Nat. Commun..

[cit12] Shi G., Zhong M., Ye F., Zhang X. (2019). Cancer Biol. Med..

[cit13] Xu Y., Li C., An J., Ma X., Yang J., Luo L., Deng Y., Kim J. S., Sun Y. (2023). Sci. China Chem..

[cit14] Ke L., Wei F., Xie L., Karges J., Chen Y., Ji L., Chao H. (2022). Angew. Chem., Int. Ed..

[cit15] Notaro A., Gasser G. (2017). Chem. Soc. Rev..

[cit16] Li J., Pu K. (2020). Acc. Chem. Res..

[cit17] Zhang T., Li Y., Zheng Z., Ye R., Zhang Y., Kwok R. T. K., Lam J. W. Y., Tang B. (2019). J. Am. Chem. Soc..

[cit18] Li X., Lee S., Yoon J. (2018). Chem. Soc. Rev..

[cit19] Fan W., Huang P., Chen X. (2016). Chem. Soc. Rev..

[cit20] Karges J., Kuang S., Maschietto F., Blacque O., Ciofini I., Chao H., Gasser G. (2020). Nat. Commun..

[cit21] Xu C., Pu K. (2021). Chem. Soc. Rev..

[cit22] Qin Y., Chen X., Gui Y., Wang H., Tang B., Wang D. (2022). J. Am. Chem. Soc..

[cit23] Heinemann F., Karges J., Gasser G. (2017). Acc. Chem. Res..

[cit24] Truksoy A., Yildiz D., Akkaya E. U. (2019). Coord. Chem. Rev..

[cit25] Cheng L., Wang C., Feng L., Yang K., Liu Z. (2014). Chem. Rev..

[cit26] Sun Y., Chen C., Liu J., Stang P. J. (2020). Chem. Soc. Rev..

[cit27] Sawada T., Fujita M. (2020). Chem.

[cit28] Xia D., Wang P., Ji X., Khashab N. M., Sessler J. L., Huang F. (2020). Chem. Rev..

[cit29] Zhu J., Xu L., Ren Y., Zhang Y., Liu X., Yin G., Sun B., Cao X., Chen Z., Zhao X., Tan H., Chen J., Li X., Yang H. (2019). Nat. Commun..

[cit30] Wang H., Liu C. H., Wang K., Wang M., Yu H., Kandapal S., Brzozowski R., Xu B., Wang M., Eswara P., Nieh M. P., Cai J., Li X. (2019). J. Am. Chem. Soc..

[cit31] Zhou J., Zhang Y., Yu G., Crawley M. R., Fulong C. R. P., Friedman A. E., Sengupta S., Sun J., Li Q., Huang F., Cook T. R. (2018). J. Am. Chem. Soc..

[cit32] Qin Y., Chen L. J., Dong F., Jiang S. T., Yin G. Q., Li X., Tian Y., Yang H. B. (2019). J. Am. Chem. Soc..

[cit33] Casini A., Woods B., Wenzel M. (2017). Inorg. Chem..

[cit34] Zhou Z., Liu J., Huang J., Rees T. W., Wang Y., Wang H., Li X., Cao H., Stang P. J. (2019). Proc. Natl. Acad. Sci. U. S. A..

[cit35] Xu Y., Tuo W., Yang L., Sun Y., Li C., Chen X., Yang W., Yang G., Stang P. J., Sun Y. (2022). Angew. Chem., Int. Ed..

[cit36] Jia P., Xu L., Hu Y., Li W., Wang X., Ling Q., Shi X., Yin G., Li X., Sun H., Jiang Y., Yang H. (2021). J. Am. Chem. Soc..

[cit37] Xu Y., Li C., Lu S., Wang Z., Liu S., Yu X., Li X., Sun Y. (2022). Nat. Commun..

[cit38] Xu Y., Li C., Ma X., Tuo W., Tu L., Li X., Sun Y., Stang P. J., Sun Y. (2022). Proc. Natl. Acad. Sci. U. S. A..

[cit39] Li X., Kolemen S., Yoon J., Akkaya E. U. (2017). Adv. Funct. Mater..

[cit40] Tu L., Li C., Liu C., Bai S., Yang J., Zhang X., Xu L., Xiong X., Sun Y. (2022). Chem. Commun..

[cit41] An R., Cheng X., Wei S., Hu Y., Sun Y., Huang Z., Chen H., Ye D. (2020). Angew. Chem., Int. Ed..

[cit42] Gupta G., Das A., Ghate N. B., Kim T., Ryu J. Y., Lee J., Mandal N., Lee C. Y. (2016). Chem. Commun..

[cit43] Reichardt C., Schneider K. R. A., Sainuddin T., Wächtler M., McFarland S. A., Dietzek B. (2017). J. Phys. Chem. A.

[cit44] Wan W., Silva M. S., McMillen C. D., Creager S. E., Smith R. C. (2019). J. Am. Chem. Soc..

[cit45] Wakamiya A., Murakami T., Yamaguchi S. (2013). Chem. Sci..

[cit46] Zhang J., Yang M., Mazi W., Adhikari K., Fang M., Xie F., Valenzano L., Tiwan A., Luo F., Liu H. (2016). ACS Sens..

[cit47] Schmid M., Brückmann J., Bösking J., Nauroozi D., Karnahl M., Ran S., Tschierlei S. (2022). Eur. J. Chem..

[cit48] Fan Y., Li C., Bai S., Ma X., Yang J., Guan X., Sun Y. (2022). Small.

[cit49] Hu W., Liu M., Zhang X. F., Shi M., Jia M., Hu X., Liu L., Wang T. (2020). J. Phys. Chem. C.

[cit50] Puckett C. A., Barton J. K. (2007). J. Am. Chem. Soc..

