# Supplementary material for: Acceptor engineering of metallacycles with high phototoxicity indices for safe and effective photodynamic therapy
Source: Chem Sci. 2023 Feb 8;14(11):2901–9. doi: 10.1039/d2sc06936a (PMC10016620; doi:10.1039/d2sc06936a)
Supplement: SC-014-D2SC06936A-s001 [file SC-014-D2SC06936A-s001.pdf]

Electronic Supplementary Material (ESI) for Chemical Science.
This journal is © The Royal Society of Chemistry 2023

Supplemental Information

Acceptor Engineering ofMetallacycles with High Phototoxicity Indexes for Safe and Effective Photodynamic Therapy

Chonglu Li,[a†] Le Tu,[a†]Jingfang Yang,[a†] Chang Liu,[a†] Yuling Xu,[a] Junrong Li,[a] Wei Tuo,[b,c] Bogdan Olenyuk,[d] Yan Sun,[b*] Peter J. Stang,[c*] Yao Sun[a*]

[a]National Key Laboratory of Green Pesticide, International Joint Research Center for Intelligent Biosensor Technology and Health, College of Chemistry, Central China Normal University, Wuhan 430079, China. E-mail: sunyaogbasp@ccnu.edu.cn

[b]Ministry of Education Key Laboratory for Special Functional Materials, Henan University, Kaifeng 475004, China. Email:elaine.sun@utah.edu

[c]Department of Chemistry, University of Utah, Salt Lake City, Utah 84112, United States. Email: stang@chem.utah.edu

[d]Proteogenomics Research Institute for Systems Medicine,505 Coast Boulevard South, La Jolla, CA 92037.

Table of Contents

Materials and Instruments 3
Synthesis and Characterization. 3
Photophysical Properties 12
Cellular Uptake, Imaging and Cytotoxicity Studies 14
In Vivo Anti-tumor Application 17
Supplementary Figures (Figures S1-S74, Table S1-S4) 19
Supplementary References 59

Materials and Instruments

All chemicals were purchased from commercial sources. ^1H, ^{13}C, 2D COSY and 2D ROESY NMR spectra were acquired on a Bruker 400 MHz magnetic resonance spectrometer. Data for ^1H NMR spectra are reported as follows: chemical shifts are reported as δ in units of parts per million (ppm) relative to chloroform-*d* (δ 7.26, s), acetonitrile-*d3* (δ 1.94, s), methanol-*d4* (δ 3.31, s); multiplicities are reported as: s (singlet), d (doublet), t (triplet), q (quartet), dd (doublet of doublets), m (multiplet), or br (broadened); coupling constants are reported as a J value in Hertz (Hz); the number of protons (n) for a given resonance is indicated nH, and based on the spectral integration values. Cells were purchased from the American Type Culture Collection.

MALDI-MS spectrometric analyses were performed on an Applied Biosystems 4700 MALDI TOF mass spectrometer. UV-Vis absorbance was recorded on a PekinElmer Lambda 25 UV-Vis spectrophotometer. The NIR-II *in vivo* system was purchased from Suzhou NIR-Optics Technologies Co., Ltd. NIR-II fluorescence spectra were recorded on an Applied NanoFluorescence spectrometer at room temperature with an excitation laser source of 808 nm.

Synthesis and Characterization

1. Synthesis of ligand L

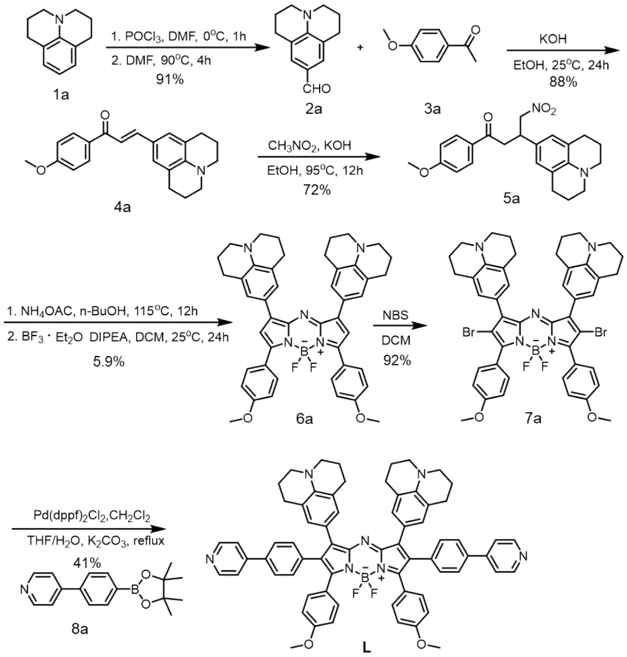

Scheme S1. The Synthesis routes of ligand **L**

Synthesis of compound 2a[S1]

DMF (3 mL) was added to freshly distilled $POCl_3$ (3 mL) under a nitrogen atmosphere at 0°C and the mixture was stirred at room temperature for 1 h. After the dropwise addition of Julolidine (8.0 g, 46.16 mmol) in DMF (20 mL), the mixture was stirred at 0°C for 5 h and then poured into an aqueous solution of $NaHCO_3$. After stirring for 2 h at room temperature, the mixture was extracted with ethyl acetate (EA), and the organic fractions were collected and dried over anhydrous Na_2SO_4. The obtained crude product was purified by silica gel chromatography (petroleum ether (PE): EA = 10: 1) to afford a yellow solid **2a** (8.4 g, 91% yield). ^{1}H NMR (400 MHz, $CDCl_3$) δ 9.55 (s, 1H), 7.24 (s, 2H), 3.25 (t, J = 5.6 Hz, 4H), 2.72 (t, J = 6.1 Hz, 4H), 1.94 – 1.89 (m, 4H). ^{13}C NMR (100 MHz, $CDCl_3$) δ 189.99, 147.88, 129.39, 123.98, 120.30, 50.02, 27.63, 21.24.

Synthesis of compound 4a [S1]

Compound **2a** (4.0 g, 19.88 mmol) and compound **3a** (2.98 g, 19.88 mmol) were dissolved in EtOH (60 mL). Potassium hydroxide (5.5 g, 99.36 mmol) was then added and the reaction mixture was stirred at room temperature for 24 h. The EtOH were removed under reduced pressure and the residue was diluted with brine and extracted with EA. The combined organic fractions were dried over anhydrous Na_2SO_4. The organic phase was subject to filtration before being concentrated under reduced pressure to afford a crude residue that was purified via flash chromatography on a silica gel column (PE: EA = 15: 1) to afford **4a** as a red solid (5.8 g, 88% yield). ^{1}H NMR (400 MHz, $CDCl_3$) δ 8.01 (d, J = 8.6 Hz, 2H), 7.70 (d, J = 15.3 Hz, 1H), 7.28 (d, J = 15.6 Hz, 1H), 7.10 (s, 2H), 6.95 (d, J = 7.7 Hz, 2H), 3.86 (s, 3H), 3.22 (t, J = 5.1 Hz, 4H), 2.74 (t, J = 6.1 Hz, 4H), 1.96 (dd, J = 11.2, 5.6 Hz, 4H). ^{13}C NMR (100 MHz, $CDCl_3$) δ 188.75, 162.90, 145.43, 145.17, 132.21, 130.39, 128.16, 121.77, 120.97, 115.55, 114.01, 55.36, 49.87, 27.99, 21.03.

Synthesis of compound 5a

Compound **4a** (2 g, 6.00 mmol) was dissolved in EtOH (20 mL), CH_3NO_2 (5.5 g, 89.98 mmol) and potassium hydroxide (336.7 mg, 6.00 mmol) were added. The reaction mixture was heated under reflux for 24 h. The EtOH were removed under reduced pressure and the residue was diluted with brine and extracted with EA. The combined organic fractions were dried over anhydrous Na_2SO_4, filtered and concentrated to afford a crude residue, which was purified via flash chromatography on a silica gel column (PE: EA=10: 1, v/v) to afford compound **5a** as a tan oil (1.7 g, 72% yield). ^{1}H NMR (400 MHz, $CDCl_3$) δ 7.91 (d, J = 8.8 Hz, 2H), 6.92 (d, J = 8.8 Hz, 2H), 6.64 (s, 2H), 4.74 (dd, J = 12.2, 6.8 Hz, 1H), 4.58 (dd, J = 12.2, 7.9 Hz, 1H), 3.98 (p, J = 7.3 Hz, 1H), 3.86 (s, 3H), 3.31 (qd, J = 17.3, 7.0 Hz, 2H), 3.12 – 3.07 (m, 4H), 2.70 (t, J = 6.4 Hz, 4H), 1.97 – 1.90 (m, 4H). ^{13}C NMR (100 MHz, $CDCl_3$) δ 195.96,

163.68, 142.45, 130.37, 129.68, 125.90, 125.80, 121.76, 113.79, 80.06, 55.50, 49.88, 41.59, 38.73, 27.65, 21.94.

Synthesis of compound 6a

Compound **5a** (1.7 g, 4.31 mmol) was dissolved in n-butanol (40 mL). Ammonium acetate (4.96 g, 64.3 mmol) was added and the reaction was stirred at 115 °C for 12 h. The reaction mixture was then cooled to room temperature and the volume was concentrated to 5 mL under reduced pressure. The reaction mixture was then filtered. The isolated solid was washed with ethanol (2× 10 mL) to afford a dark blue solid with metallic luster. The blue solid was dissolved in anhydrous dichloromethane (DCM). Anhydrous N,N-diisopropylethylamine (DIPEA) (843.6 mg, 6.52 mmol, 1.14 mL) and boron trifluoride etherate (1.20 g, 8.58 mmol, 1.08 mL) were added and then stirred at room temperature for 24 h under N_2. The resulting solution was diluted with water (40 mL) and extracted with DCM. The combined organic fractions were dried over anhydrous Na_2SO_4, filtered and concentrated under reduced pressure to afford a crude residue, which was purified via flash chromatography on a silica gel column (PE: DCM=1: 1, v/v) to afford **6a** as a metallic brown solid (380 mg, 5.9% yield). ^{1}H NMR (400 MHz, $CDCl_3$) δ 8.01 (d, J = 8.7 Hz, 4H), 7.60 (s, 4H), 6.96 (d, J = 8.7 Hz, 4H), 6.73 (s, 2H), 3.86 (s, 6H), 3.29 – 3.25 (m, 8H), 2.77 (t, J = 5.9 Hz, 8H), 2.02 – 1.96 (m, 8H). ^{13}C NMR (100 MHz, $CDCl_3$) δ 160.94, 155.99, 145.16, 143.86, 143.10, 131.06, 128.53, 125.31, 121.14, 120.78, 113.83, 55.31, 50.12, 28.02, 21.80.

Synthesis of compound 7a

Compound **6a** (112 mg, 0.148 mmol) was dissolved in dichloromethane, and then N-bromosuccinimide (8.5 mg, 0.148 mmol) was added to the solution. The reaction mixture was stirred at room temperature for 1 h. After diluting with dichloromethane (100 mL), the organic layer was washed with saturated aqueous brine (100 mL). It was then dried over anhydrous magnesium sulfate and filtered as well as concentrated to dryness. The crude product was purified by silica gel chromatography (PE:DCM = 1:1, v/v) to give **7a** as a blue-green solid (124 mg, 92% yield). ^{1}H NMR (400 MHz, $CDCl_3$) δ 7.74 (d, J = 8.3 Hz, 4H), 7.58 (s, 4H), 7.00 (d, J = 8.1 Hz, 4H), 3.88 (s, 6H), 3.32 – 3.27 (m, 8H), 2.83 (t, J = 6.1 Hz, 8H), 2.04 (dd, J = 11.1, 5.5 Hz, 8H). ^{13}C NMR (100 MHz, $CDCl_3$) δ 160.89, 155.11, 144.25, 143.99, 141.92, 132.17, 130.18, 122.93, 120.64, 119.09, 113.30, 55.21, 50.11, 27.78, 21.75.

Synthesis of compound 8a

4-(4-bromophenyl)pyridine[S4] (410 mg, 1.75 mmol), bis(pinacolate)diboron (800 mg, 3.16 mmol) and KOAc (472 mg, 4.21 mmol) were dissolved in 1,4-dioxane (30 mL), then bis(triphenylphosphine)palladium (II) dichloride (31 mg, 0.044 mmol) was added and the reaction mixture

was stirred at 100 °C for 12 h under an argon atmosphere. The reaction solution was extracted with ethyl acetate and water, dried and distilled under reduced pressure. The crude product was purified by flash column chromatography on silica gel (PE: EA=4: 1). The compound **8a** was obtained as white solid (375 mg, 76 % yield). ^{1}H NMR (400 MHz, $CDCl_3$) δ 8.64 (d, J = 6.0 Hz, 2H), 7.91 (d, J = 8.1 Hz, 2H), 7.63 (d, J = 8.1 Hz, 2H), 7.51 (dd, J = 4.7, 1.3 Hz, 2H), 1.35 (s, 12H). ^{13}C NMR (100 MHz, $CDCl_3$) δ 150.05, 148.20, 140.49, 135.53, 126.25, 121.73, 84.01, 83.98, 74.76, 24.87.

Synthesis of ligand L

Compound **7a** (60 mg, 0.066 mmol) and compound **8a** (56 mg, 0.198 mmol) were dissolved in THF (8 mL). Potassium carbonate (11 mg, 0.081 mmol) in 2 mL distilled water and 1,1'-bis(diphenylphosphino) ferrocenepalladium(II) dichloride dichloromethane complex (3.6 mg, 0.0042 mmol) were added under argon atmosphere. The mixture was heated in an oil bath at 75°C for 8 h. After cooling to room temperature, the volatiles were removed *in vacuo*. The residue was dissolved in dichloromethane, and the resulting solution was washed with water and then saturated brine. After drying over anhydrous magnesium sulfate and removal of the solvents under reduced pressure, the crude product was purified by silica gel chromatography (pure EA) to afford a blue solid **L** (25 mg, 41% yield). ^{1}H NMR (400 MHz, $CDCl_3$) δ 8.64 (s, 4H), 7.50 (d, J = 6.9 Hz, 8H), 7.37 (d, J = 7.6 Hz, 4H), 7.15 (d, J = 7.0 Hz, 4H), 7.06 (s, 4H), 6.73 (d, J = 7.4 Hz, 4H), 3.74 (s, 6H), 3.17 (d, J = 4.4 Hz, 9H), 2.51 (t, J = 5.5 Hz, 8H), 1.92 – 1.87 (m, 8H). ^{13}C NMR (100 MHz, $CDCl_3$) δ 160.18, 149.95, 148.19, 143.49, 140.94, 136.47, 135.63, 132.15, 131.74, 130.55, 126.57, 123.66, 121.36, 120.43, 119.93, 113.16, 55.10, 50.04, 29.71, 27.55, 21.92.

2. Synthesis of acceptor A1, A2, A3 and A4

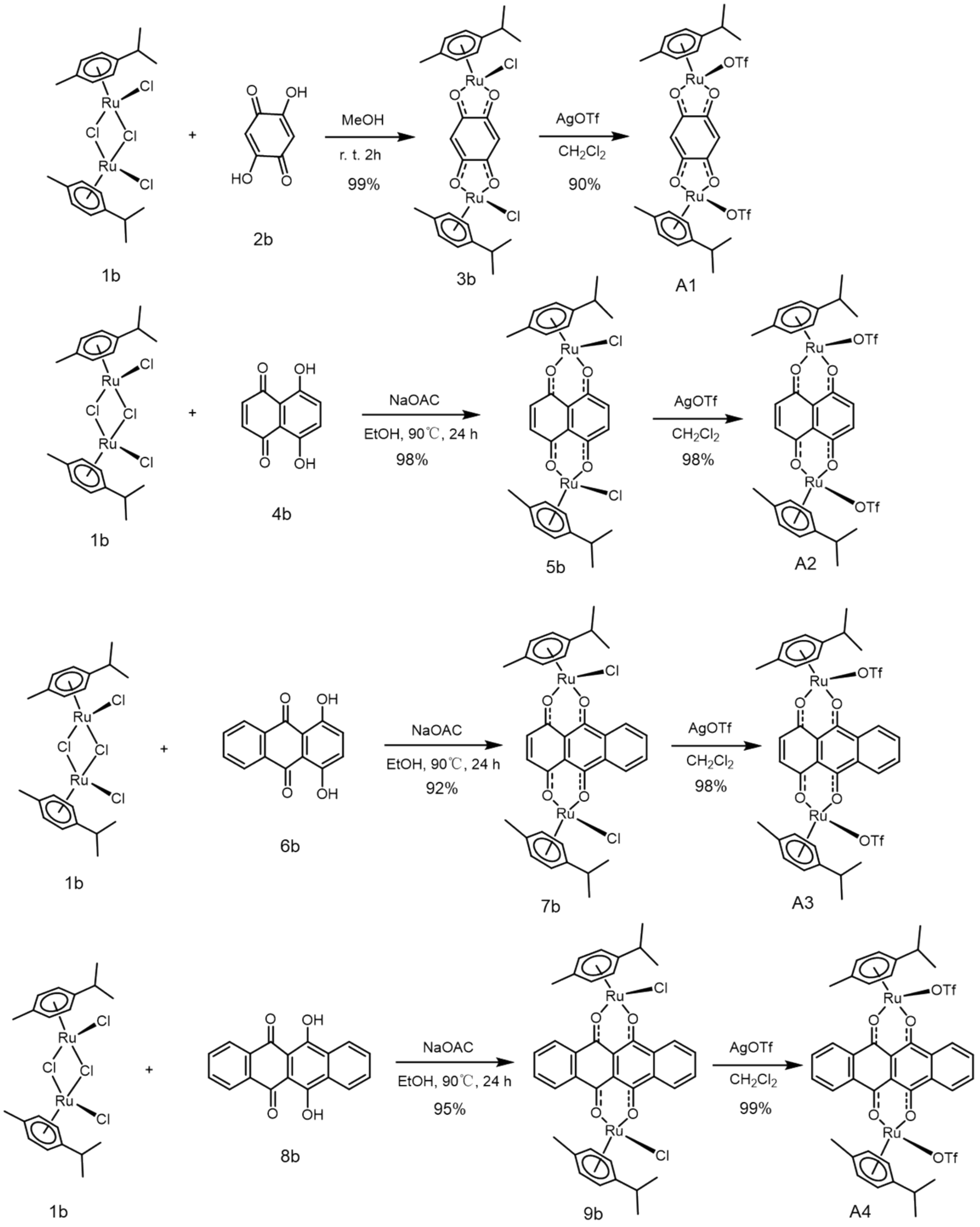

Scheme S2. The Synthesis routes of acceptor **A1**, **A2**, **A3** and **A4**

Synthesis of compound 3b [S2]

Compound **1b** (100 mg, 0.163 mmol) and compound **2b** (22.9 mg, 0.163 mmol) were dissolved in anhydrous methanol (20.0 mL) under argon atmosphere. The mixture was stirred at room temperature for 3 h. After the reaction was completed, the reaction mixture was concentrated under reduced pressure, subject to suction filtration and washed with ethanol, water, acetone, and ether to obtain product **3b** as a

brown solid (110 mg, 99% yield). ^{1}H NMR (400 MHz, $CDCl_3$) δ 5.80 (s, 2H), 5.63 (d, J = 6.0 Hz, 4H), 5.38 (d, J = 5.9 Hz, 4H), 2.91 (dd, J = 13.8, 6.6 Hz, 2H), 2.29 (s, 6H), 1.33 (d, J = 6.9 Hz, 12H). ^{13}C NMR (100 MHz, $CDCl_3$) δ 101.88, 93.81, 84.66, 81.14, 79.20, 43.34, 31.29, 22.46, 18.67.

Synthesis of compound A1 [S2]

Compound **3b** (110 mg, 0.16 mmol) and silver trifluoromethanesulfonate (123.3 mg, 0.48 mmol) were dissolved in DCM and stirred at room temperature for 3 h. The black-green reaction solution was subject to suction filtration and washed with methanol to give product **A1** (130.6 mg, 90% yield). ^{1}H NMR (400 MHz, CD_3CN) δ 6.03 (t, J = 5.5 Hz, 4H), 5.86 (d, J = 11.0 Hz, 2H), 5.76 (t, J = 6.1 Hz, 4H), 2.90 (dt, J = 13.6, 6.8 Hz, 2H), 2.30 (d, J = 5.1 Hz, 6H), 1.37 (d, J = 4.2 Hz, 12H). ^{13}C NMR (100 MHz, CD_3CN) δ 185.48, 185.26, 103.63, 102.07, 101.97, 101.02, 99.61, 84.67, 81.35, 31.77, 22.09, 18.30.

Synthesis of compound 5b [S2]

Compound **1b** (980 mg, 1.60 mmol), sodium acetate (280 mg, 3.42 mmol) and compound **4b** (360 mg, 1.90 mmol) were added to absolute ethanol (40.0 mL) under an argon atmosphere. The reaction mixture was heated in an oil bath at 80°C for 24 h. After allowing to cool, the reaction mixture was concentrated under reduced pressure, subject to suction filtration and washed with ethanol, water, acetone, and ether to obtain product **5b** as a black solid (1.14 g, 98% yield). ^{1}H NMR (400 MHz, $CDCl_3$) δ 6.95 (s, 4H), 5.49 (d, J = 5.9 Hz, 4H), 5.23 (d, J = 5.9 Hz, 4H), 2.85 (dd, J = 13.9, 7.0 Hz, 2H), 2.22 (s, 6H), 1.31 (d, J = 6.9 Hz, 12H). ^{13}C NMR (100 MHz, $CDCl_3$) δ 170.97, 137.06, 100.37, 98.00, 82.87, 79.66, 30.77, 22.38, 17.94.

Synthesis of compound A2 [S2]

Compound **5b** (1.14 g, 1.56 mmol) and silver trifluoromethanesulfonate (0.82 g, 3.20 mmol) were dissolved in DCM and stirred at room temperature for 3 h. The black-green reaction solution was subject to suction filtration and washed with methanol to give product **A2** (1.14 g, 98 % yield). ^{1}H NMR (400 MHz, CD_3CN) δ 7.20 (s, 4H), 5.88 (d, *J* = 6.2 Hz, 4H), 5.57 (d, *J* = 6.2 Hz, 4H), 2.90 (dd, *J* = 13.8, 6.9 Hz, 2H), 2.23 (s, 6H), 1.36 (d, *J* = 6.9 Hz, 12H). ^{13}C NMR (100 MHz, CD_3CN) δ 171.79, 138.00, 123.26, 120.07, 87.97, 85.83, 85.14, 81.37, 31.23, 21.96, 21.79, 17.43.

Synthesis of compound 7b [S2]

Compound **1b** (100 mg, 0.163 mmol), sodium acetate (26.8 mg, 0.326 mmol) and compound **6b** (39.23 mg, 0.163 mmol) were added to absolute ethanol (20.0 mL) under an argon atmosphere. The mixture was heated in an oil bath at 80°C for 24 h. After allowing to cool, the reaction mixture was concentrated under reduced pressure, subject to suction filtration and washed with ethanol, water, acetone, and ether

continuously to give product **7b** as a black solid (116 mg, 92% yield). ^{1}H NMR (400 MHz, $CDCl_3$) δ 5.80 (s, 2H), 5.63 (d, *J* = 6.0 Hz, 4H), 5.38 (d, *J* = 5.9 Hz, 4H), 2.91 (dd, *J* = 13.8, 6.6 Hz, 2H), 2.29 (s, 6H), 1.33 (d, *J* = 6.9 Hz, 12H).^{13}C NMR (100 MHz, $CDCl_3$) δ 170.90, 169.70, 137.36, 134.02, 131.60, 126.83, 110.24, 99.98, 97.82, 83.46, 82.72, 79.36, 79.30, 30.85, 22.51, 22.43, 17.96.

Synthesis of compound A3 [S2]

Compound **7b** (116 mg, 0.15 mmol) and silver trifluoromethanesulfonate (115.6 mg, 0.45 mmol) were dissolved in DCM and stirred at room temperature for 3 h. The black-green reaction solution was subject to suction filtration and washed with methanol to give product **A3** (148 mg, 98% yield). ^{1}H NMR (400 MHz, CD_3CN) δ 8.55 (dd, *J* = 5.9, 3.4 Hz, 2H), 7.94 (dd, *J* = 5.8, 3.3 Hz, 2H), 7.25 (s, 2H), 5.96 (d, *J* = 6.1 Hz, 4H), 5.63 (d, *J* = 6.1 Hz, 4H), 2.97 (dq, *J* = 13.5, 6.8 Hz, 2H), 2.31 (s, 6H), 1.41 (d, *J* = 6.9 Hz, 12H). ^{13}C NMR (100 MHz, CD_3CN) δ 170.99, 169.72, 137.60, 133.35, 133.11, 127.07, 109.66, 102.66, 101.35, 85.12, 80.56, 30.67, 21.43, 17.04.

Synthesis of compound 9b [S2]

Compound **1b** (100 mg, 0.163 mmol), sodium acetate (26.8 mg, 0.326 mmol) and compound **8b** (47.4 mg, 0.163 mmol) were added to absolute ethanol (20.0 mL) under an argon atmosphere. The mixture was heated in an oil bath at 80°C for 24 h. After allowing to cool, the reaction mixture was concentrated under reduced pressure, subject to suction filtration and washed with ethanol, water, acetone, and ether continuously to give product **9b** as a blue solid (128 mg, 95% yield). ^{1}H NMR (400 MHz, $CDCl_3$) δ 8.49 (dd, *J* = 6.0, 3.4 Hz, 4H), 7.67 (dd, *J* = 6.0, 3.4 Hz, 4H), 5.66 (d, *J* = 5.9 Hz, 4H), 5.38 (d, *J* = 5.9 Hz, 4H), 3.14 – 3.04 (m, 2H), 2.37 (s, 6H), 1.48 (d, *J* = 7.0 Hz, 12H). ^{13}C NMR (100MHz, $CDCl_3$) δ 169.50, 134.59, 131.20, 126.80, 99.88, 97.49, 83.14, 79.41, 30.92, 22.55, 18.08.

Synthesis of compound A4 [S2]

Compound **9b** (128 mg, 0.154 mmol) and silver trifluoromethanesulfonate (118.7 mg, 0.462 mmol) were dissolved in DCM and stirred at room temperature for 3 h. The black-blue reaction solution was subject to suction filtration and washed with methanol to give product **A4** (162 mg, 99% yield). ^{1}H NMR (400 MHz, CD_3CN) δ 8.62 (dd, *J* = 6.0, 3.3 Hz, 4H), 7.94 (dd, *J* = 5.9, 3.3 Hz, 4H), 6.03 (d, *J* = 6.1 Hz, 4H), 5.71 (d, *J* = 6.0 Hz, 4H), 3.07 (dt, *J* = 13.8, 6.9 Hz, 2H), 2.39 (s, 6H), 1.44 (d, *J* = 6.9 Hz, 12H). ^{13}C NMR (100 MHz, CD_3CN) δ 169.40, 133.89, 132.71, 126.94, 107.10, 102.62, 101.36, 85.10, 80.58, 30.72, 21.51, 17.36.

3. Synthesis of metallacycles Ru1-Ru4 and Ru-M

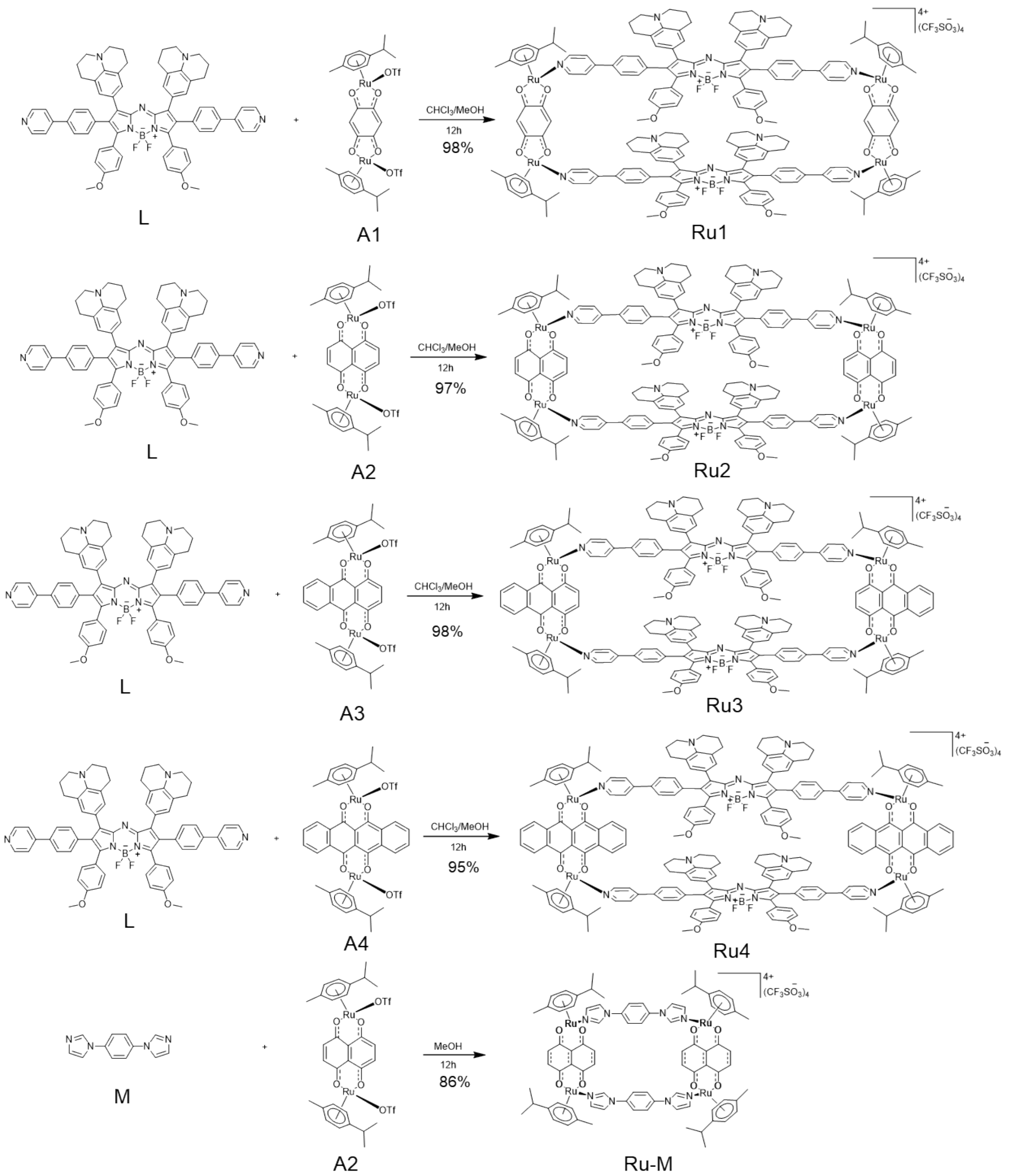

Scheme S3. The Synthesis routes of metallacycles **Ru1-Ru4** and **Ru-M**

Synthesis of compound Ru1

At a 1:1 molar ratio, the **L** (8.0 mg, 0.0076 mmol) and **A1** (7.26 mg, 0.0076 mmol) were placed in an 8 mL of vial, followed by the addition of $CHCl_3/CH_3OH$= 2/1 (12 mL). After stirring at ambient temperature for 24 h, the solution was concentrated to 0.5 mL. The self-assembly products were isolated via precipitation by adding diethyl ether into the concentrated solution, washing twice with diethyl ether and drying under vacuum to obtain product **Ru1** (14.6 mg, 98% yield). ^1H NMR (400 MHz, CD_3CN) δ 8.09 (d, J = 6.3 Hz, 8H), 7.46 (d, J = 6.1 Hz, 8H), 7.20 (d, J = 8.4 Hz, 8H), 7.09 (d, J = 8.5 Hz, 8H), 7.05

(s, 8H), 6.90 (d, J = 8.2 Hz, 8H), 6.37 (d, J = 7.8 Hz, 8H), 5.85 (d, J = 6.4 Hz, 8H), 5.66 (s, 4H), 5.64 (d, J = 6.1 Hz, 8H), 3.61 (s, 12H), 3.19 – 3.15 (m, 16H), 2.79 (d, J = 6.8 Hz, 4H), 2.38 (t, J = 6.0 Hz, 16H), 2.11 (s, 12H), 1.83 – 1.80 (m, 16H), 1.29 (s, 24H). ESI-TOF-MS is shown in Figure S21: m/z = 831.38 $[M{-}4OTf]^{4+}$.

Synthesis of compound Ru2

At a 1:1 molar ratio, the **L** (10.0 mg, 0.0095 mmol) and **A2** (9.09 mg, 0.0095 mmol) were placed in an 8 mL of vial, followed by the addition of $CHCl_3/CH_3OH$= 2/1 (10 mL). After stirring at ambient temperature for 24 h, the solution was concentrated to 0.5 mL. The self-assembly products were isolated via precipitation by adding diethyl ether into the concentrated solution, washing twice with diethyl ether and drying under vacuum to obtain product **Ru2** (15.8 mg, 97% yield).^{1}H NMR (400 MHz, CD_3CN) δ 8.27 (d, J = 5.8 Hz, 8H), 7.43 (d, J = 5.6 Hz, 8H), 7.24 (d, J = 7.7 Hz, 9H), 7.15 (s, 8H), 7.12 (d, J = 8.5 Hz, 8H), 7.03 (s, 8H), 6.92 (d, J = 7.7 Hz, 7H), 6.41 (d, J = 7.8 Hz, 8H), 5.68 (d, J = 6.0 Hz, 8H), 5.48 (d, J = 6.0 Hz, 8H), 3.21 – 3.12 (m, 17H), 2.81 (d, J = 7.0 Hz, 4H), 2.38 (s, 16H), 1.83 (d, J = 9.9 Hz, 17H), 1.25 (s, 24H). ESI-TOF-MS is shown in Figure S22: m/z = 856.23 $[M{-}4OTf]^{4+}$, 1191.96 $[M{-}3OTf]^{3+}$.

Synthesis of compound Ru3

At a 1:1 molar ratio, the **L** (5.0 mg, 0.0047 mmol) and **A3** (4.78 mg, 0.0047 mmol) were placed in an 20 mL of vial, followed by the addition of $CHCl_3/CH_3OH$= 2/1 (8 mL). After stirring at ambient temperature for 24 h, the solution was concentrated to 0.5 mL. The self-assembly products were isolated via precipitation by adding diethyl ether into the concentrated solution, washing twice with diethyl ether and drying under vacuum to obtain product **Ru3** (9.5 mg, 98% yield). ^{1}H NMR (400 MHz, CD_3CN) δ 8.68 (s, 4H), 8.38 (d, *J* = 5.3 Hz, 8H), 7.98 (s, 4H), 7.42 (s, 8H), 7.26 (s, 4H), 7.23 (d, *J* = 6.5 Hz, 8H), 7.14 (d, *J* = 6.7 Hz, 8H), 7.07 (s, 8H), 6.94 – 6.91 (m, 8H), 6.38 (d, *J* = 18.9 Hz, 6H), 5.81 (dd, *J* = 19.1, 6.2 Hz, 16H), 5.61 (d, *J* = 3.7 Hz, 8H), 3.64 (s, 12H), 3.23 – 3.19 (m, 16H), 2.92 (d, *J* = 7.0 Hz, 4H), 2.43 – 2.38 (m, 16H), 2.21 (s, 12H), 1.87 – 1.82 (m, 16H), 1.36 (d, *J* = 2.6 Hz, 24H). ESI-TOF-MS is shown in Figure S23: m/z = 811.40 $[M{-}4OTf]^{4+}$, 1224.86 $[M{-}3OTf]^{3+}$.

Synthesis of compound Ru4

At a 1:1 molar ratio, the **L** (8.0 mg, 0.0076 mmol) and **A4** (8.02 mg, 0.0076 mmol) were placed in an 8 mL of vial, followed by the addition of $CHCl_3/CH_3OH$= 2/1 (12 mL). After stirring at ambient temperature for 24 h, the solution was concentrated to 0.5 mL.The self-assembly products were isolated via precipitation by adding diethyl ether into the concentrated solution, washing twice with diethyl ether and drying under vacuum to obtain product **Ru4** (15.2 mg, 95% yield). ^{1}H NMR (400 MHz, CD_3CN) δ

8.76 (dd, J = 5.9, 3.5 Hz, 8H), 8.43 (d, J = 6.6 Hz, 8H), 7.98 (dd, J = 6.0, 3.3 Hz, 8H), 7.37 (d, J = 5.5 Hz, 8H), 7.17 (d, J = 8.4 Hz, 8H), 7.10 (d, J = 8.1 Hz, 8H), 7.05 (s, 8H), 6.88 (d, J = 8.0 Hz, 8H), 6.37 (d, J = 9.9 Hz, 8H), 5.91 (d, J = 6.2 Hz, 8H), 5.70 (d, J = 6.3 Hz, 8H), 3.61 (s, 12H), 3.23 – 3.16 (m, 16H), 3.02 – 2.98 (m, 4H), 2.38 (t, J = 6.1 Hz, 16H), 2.24 (s, 12H), 1.85 – 1.79 (m, 16H), 1.36 (d, J = 6.9 Hz, 24H).ESI-TOF-MS is shown in Figure S24: m/z = 906.64 $[M-4OTf]^{4+}$, 1207.85 $[M-3OTf]^{3+}$.

Synthesis of compound M [S3]

1,4-Dibromobenzene (2.0 g, 8.5 mmol), imidazole (2.4 g, 35.6 mmol), K_2CO_3 (3.75 g, 27.2 mmol) and $CuSO_4$ (0.027 g, 0.17 mmol) were stirred and heated at 180 °C for 12 h. After the reaction was completed, the mixture was cooled to room temperature, and water was added to wash the mixture. The crude product was dissolved in ethanol (30 mL). The organic layer was separated. The organic layer was evaporated to dryness to give the crude product. The residue was recrystallized from water and methanol to give a white solid (1.5 g, 86% yield). ^{1}H NMR (400 MHz, $CDCl_3$) δ 7.90 (s, 2H), 7.54 (s, 4H), 7.32 (s, 2H), 7.25 (s, 2H). ^{13}C NMR (100 MHz, $CDCl_3$) δ 136.42, 135.53, 130.85, 118.19.

Synthesis of compound Ru-M

In a 1:1 molar ratio, the **M** (10 mg, 0.048 mmol) and **A2** (47.52 mg, 0.048 mmol) were placed in an 5 mL of vial, followed by the addition of CH_3OH (5 mL). After stirring at ambient temperature for 24 h, the solution was concentrated to 0.5 mL, self-assembly products were isolated via precipitation by addition of diethyl ether into concentrated solution, washed twice with diethyl ether and dried under vacuumto obtain product **Ru-M** (48 mg, 85% yield). ^{1}H NMR (600 MHz, CD_3OD) δ 8.29 (s, 4H), 7.49 (s, 8H), 7.47 (s, 4H), 7.09 (s, 8H), 6.91 (s, 4H), 5.75 (d, *J* = 5.9 Hz, 8H), 5.53 (d, *J* = 5.8 Hz, 8H), 2.75 – 2.71 (m, 4H), 2.04 (s, 12H), 1.23 (d, *J* = 6.8 Hz, 24H). ^{13}C NMR (150 MHz, CD_3OD) δ 172.00, 138.22, 136.54, 130.36, 123.27, 120.79, 112.42, 103.80, 100.98, 85.71, 82.63, 31.77, 22.25, 17.29.

Photophysical properties

1. Absorption and photoluminescence excitation (PLE) spectral studies

The UV-Vis-NIR absorbance spectra of **Ru1-Ru4** were recorded on a PerkinElmer Lambda 25 UV-Vis spectrophotometer. PLE spectra of **Ru1-Ru4** solutions were obtained using an Applied Nano Fluorescence spectrometer.

2. Stability tests of Ru1-Ru4.

For chemical-stability tests, **Ru1**-**Ru4** (10 μM) was incubated in PBS or 10% FBS and stored for a whole week. The UV-Vis absorption of **Ru1**-**Ru4** was measured every day. The photostability of **Ru1**-**Ru4** was investigated by recording the absorption spectra, for which the UV-Vis absorption of **Ru1**-**Ru4** (10 μM) was measured after 808 nm laser illumination (1 W/cm^2) for various time (0, 10, 20, 30 min).

3. The FL penetration depths measurement

To detect the tissue penetration depth response of Ru metallacycles, 1% intralipid was used to mimic biological tissue (5 mL 20% intralipid were mixed with 100 mL water). Glass capillary tubes were filled with either **Ru1**-**Ru4** (200 μM) or **Ru-M1** (200 μM) and taped to the bottom of a cylindrical dish. The dish was filled with different volumes of intralipid that approximates the wavelength dependence of light scattering in biological tissues. The depth of the capillary tubes was calculated from the area of the dish. The fluorescence images were then obtained by using 808 nm laser illumination for **Ru1**-**Ru4** or **Ru-M1** images were analyzed by ImageJ software.

4. The ROS penetration depths measurement

To examine the tissue penetration depth response of **Ru1**-**Ru4**, biological tissue was simulated using 1% intralipid (5 mL of 20% adipose lactide mixed with 100 mL of water). A 96 well plates were filled with a solution of DCFH treated with **Ru1**-**Ru4** (10 μM) or **Ru-M1** (10 μM). Petri dishes were filled with different volumes of fat, which approximated the wavelength dependence of light scattering in biological tissue. The depth of the capillary is calculated from the area of the petri dish. Place the culture dish on the top of 96 well plate, and then irradiated with 808 nm laser for 3 min to obtain fluorescence image, and the images were analyzed by ImageJ software.

5. Test of ROS generation *in vitro*

The ROS generation capacity of **Ru1**-**Ru4** was evaluated using a classic ROS indicator DCFH-DA (2’,7’-dichlorodihydrofluorescein diacetate).[S5] DCFH-DA (MedChem Express, USA) was dissolved in DMSO (1.0 mM, 0.8 mL) and mixed with NaOH (0.01 M, 2 mL).The resulting deacetylation gave DCFH. The DCFH (20 μM) obtained in this way was added into a DMSO solution of **Ru1**-**Ru4** (10 μM). The solution was then subject to 808 nm laser photoirradiation (1 W/cm^2) for 0, 15, 30, 45 and 60 s. The fluorescent spectrum of DCF (λ_{ex}= 488 nm, λ_{em}= 525 nm) was recorded.

6. Detection of intracellular ROS generation.

A549 cells were seeded on confocal dishes (~5 × 10^4 cells/dish). After cells were incubated with **Ru4** (10 μM) for 6 h, the medium was replaced by DCFH-DA (20 μM) for further incubation (20 min). Then the

medium was replaced with fresh medium, and cells were illuminated with 808 nm laser (1 W/cm^2) for 5 min and subsequently visualized by an invert fluorescence microscope (λ_{ex} = 488 nm; λ_{em} = 520–550 nm).

7. Superoxide anion radical ($O_2^{-\bullet}$) detection

The light triggered $O_2^{\bullet-}$ generation was measured. Detection of $O_2^{\bullet-}$ was performed by taking advantage of the interaction between dihydroethidine and DNA. Dihydroethidium (DHE) was utilized as $O_2^{\bullet-}$ specific probe because it could intercalate in DNA and emit red fluorescence at the participation of $O_2^{\bullet-}$. Briefly, photosensitizer (**Ru1-Ru4**, 10 μM) and DHE (50 μM) were dissolved in a water solution that contained 250 μg/mL of ctDNA. A mixture only containing DHE and ctDNA was used as the control. The mixtures were irradiated with the 808 nm laser (1 W/cm^2). After different irradiation times, fluorescence spectra (excited at 490 nm) of these mixtures were recorded using a Edinburgh FL900/FS900 spectro fluorometer.

8. Detection of intracellular $O_2^{\bullet-}$ generation.

About 1 × 10^5 A549 cells in Dulbecco's modified Eagle's medium (DMEM) were seeded in confocal dishes (Corning) and incubated overnight at 37 °C under a humidified 5% CO_2 atmosphere. The intracellular $O_2^{\bullet-}$ was examined by using DHE as a fluorescence probe. A549 cells were incubated with **Ru4** (10 μM) for 2 h followed by incubation with 10 μM DHE for 30 min. After then washed with PBS for two times, cells were irradiated with a 808 nm laser 1 W/cm^2 for 5 min). Then, the fluorescence was immediately observed using a Leica laser fluorescent confocal microscope (CLSM) with the excitation wavelength of 488 nm, and emission collection wavelength was 570 nm to 630 nm.

9. Theoretical calculation.

Equilibrium geometries at the ground state were optimized using the density functional theory (DFT) method with the B3LYP functional through Gaussian 16. The 6-31G(d) basis set was used for H, C, N, and S atoms, and the Los Alamos National Laboratories (LANL2DZ) basis set and pseudopotential were used for Ru. The vibrational frequencies of all the optimized structures were calculated to ensure that the optimized structures of molecules have no imaginary vibrational frequencies. The minimized structures were used for the orbital generation and analyzed. The bending angle is defined by dihedral angle∠N1−N2−B1−N3, which represents the angle between the phenylpyridine modified groups on both sides of the aza-BODIPY core.

Cellular Uptake, Imaging and Cytotoxicity Studies

1. Intracellular colocalization monitored by CLSM

A549 cells were seeded onto 35 mm confocal dishes at a density of 1 × 10^4 cells/mL and allowed to adhere overnight. The cells were loaded with **Ru4** (10 μM, 1% DMSO, *v*%) for 6 h at 37 °C in the dark. The cells were then incubated with LysoTracker® Green (100 nM), MitoTracker® Deep Red (500 nM) and Hoechst 33342 (5 μg/mL) (Thermo Fisher Scientific (USA)) for 45 min at 37 °C in the dark. The cells were washed with PBS three times at the end of every period. Confocal images were taken using a laser scanning confocal microscope (LSM 710, Carl Zeiss, Germany). For the LysoTracker® Green channel, the excitation wavelength was 488 nm, and the signal was collected between 515-535 nm. For the MitoTracker® Deep Red channel, the probe was excited at 644 nm, and the emission filter was between 665-700 nm. For the Hoechst 33342 channel, the excitation wavelength was 405 nm, and the fluorescence signal was recorded at 430-460 nm. For **Ru4**, an excitation wavelength of 808 nm was used, and the emission filter was between 1000-1100 nm.

2. Elemental imaging by LA-ICP-MS.

A549 cells were seeded in 24-well cultureplates with cell climbing slices for overnight and then incubated with **Ru4** (10 μM) for 6 h. After washed by PBS w/o Ca/Mg, cells were fixed with 70% coldalcohol solution and washed by water. Then, cells were allowed to adhere on slidesto prepare LA-ICP-MS test. A laser spot size of 3 μm diameter, 10 μm s^{-1} scanspeed, 100 Hz repetition frequency, and laser fluence of ~3 J cm^{-2} were utilized to perform the test. ICP-MS parameters were as follows: radio frequency power of 1500 W, nebulizer gas flow of 1.25 L min^{-1}, auxiliary gas flow of 1.2 L min^{-1}, andplasma gas flow of 15 L min^{-1}. The monitored isotope ^{102}Ru was measured incounting mode. Images integration was performed by the IGOR-based lolite software.

3. Cellular uptake of the ruthenium content measured by ICP-MS

A549 cells were seeded at a density of 1 × 10^6 cells in 6-well cell culture plates. The cells were left to grow for 24 h in DMEM medium containing 10% FBS and 1% penicillin/streptomycin at 37 °C in 5% CO_2 humidified atmosphere. After 12 h, **Ru4** (10 μM) was added into the wells and the cells were incubated for 1 h, 3 h, 6 h, and 12 h, respectively. Following incubation, the cells were washed, digested and collected, and the Ru content in the cells determined by ICP-MS. All experiments were repeated three times.

4. Cellular uptake mechanism studies

Cellular imaging was carried out to examine the cellular uptake mechanism.[S4] A549 cells were seeded on 35 mm confocal dishes at a density of 1 × 10^4 cells/mL and allowed to adhere overnight. The culture medium was refreshed with PBS. For the temperature-dependent uptake study, A549 cells were incubated

with 10 μM **Ru4** (1% DMSO, *v*%) for 6 h at 37 °C and 4 °C, respectively. For the cellular uptake inhibition study, triethylamine (1 mM) was used as anion channel inhibitor. Methyl-β-cyclodextrin (50 mM) and sucrose (5 μM) were adopted as caveolin-mediated and clathrin-mediated inhibitors, respectively, while NH_4Cl (50 mM) and chloroquine (100 μM) were used as endocytic inhibitors. A549 cells were pretreated with these protein inhibitors for 40 min at 37 °C, respectively. PBS was then used to wash the cells and the cells were further incubated solely with 10 μM **Ru4** (1% DMSO, *v*%) for 6 h at 37°C. All of the cells were then washed with PBS three times and subjected to confocal microscopy.

5. Cell viability studies

In vitro cytotoxicity of **Ru1-Ru4**, **A1-A4**, and **L** was determined by means of MTT assays using several human cell lines, including A549 and 16HBE. For instance, A549 cells were seeded onto 96-well plates at 0.5×10^4 cells per well and incubated at 37ºC for 24 h. For normoxic photo-cytotoxicity evaluation, different concentrations of **Ru1-Ru4** in DMEM medium were added to the wells of normoxic cells, respectively. For hypoxic photo-cytotoxicity evaluation, different concentrations of **Ru1-Ru4** in DMEM medium were added to the wells of cells, and placed in closed anoxic sealed bags (O_2 content < 1%). Then, the cells were further incubated for 6 h under normoxic or hypoxic conditions, respectively. Subsequently, the cells were subjected to 808 nm laser (1 W/cm^2, 5 min) under respective conditions. Then the cells were further incubated for 12 h at 37 ºC. The addition of 10 μL of MTT (BioFrox, China) as a 0.5 mg/mL solution to each well was followed by incubation for 4 h at 37°C to allow the formation of formazan crystals. Then, the supernatant was removed and the products were lysed with 200 μL of DMSO. The absorbance value was recorded at 570 nm using a microplate reader. The absorbance of the untreated cells was used as a control and its absorbance was used as the reference value for calculating 100% cellular viability.

6. Annexin V-FITC staining assay

A549 cells were seeded in 12-well plates at a density of 1×10^6 cells/well. The dark groups containing **Ru4** were incubated in the dark for 24 h. For the light groups, after incubation with **Ru4** for 12 h, the cells were exposed to 808 nm irradiation for 5 min and then allowed to incubate for another 12 h in the dark. Cells in the control group were treated with aculture medium. The cells were further live stained with annexin V-FITC following the protocols of the manufacturer. Cells were imaged before and after being subject to 5 min of laser irradiation (808 nm, 1 W/cm^2). Finally, the samples prepared in this way were analyzed via flow cytometry (CytoFLEX, Beckman Coulter).

7. Lysosomes disruption assay

A549 cells were subject to different treatments: 1) untreated; 2) irradiated with 808 nm laser irradiation (1 W/cm^2) for 5 min; 3) incubated with 10 μM **Ru4** for 24 h; 4) incubated with 10 μM **Ru4** for 24 h and then irradiated with 808 nm laser (1 W/cm^2). After treatment, cells were incubated with acridine orange (Macklin, China) at a concentration of 10 μM for 20 min and subjected to confocal luminescence imaging. Confocal luminescence imaging was performed with excitation at 488 nm and monitoring at 505−545 nm for the green channel or 617-640 nm for the red channel.

8. Analysis of mitochondrial membrane potential (MMP)

MMP was assessed by means of JC-1 staining. A549 cells were seeded onto corning confocal dishes at a density of 1 × 10^4 cells/mL and allowed to adhere overnight. The cells were then treated with culture medium (control) or 10 μM **Ru4** (1% DMSO, *v*%), respectively. The cells were incubated at 37ºC for 2 h in the dark and then washed with PBS. The cells were then cultured with JC-1 (Solarbio, China) (5 μM) in PBS at room temperature for 20 min in the dark. Fluorescent images were captured by CLSM before and after 808 nm laser irradiation (1 W/cm^2). The excitation wavelength for the JC-1 monomer was 488 nm, and the emission filter was adjusted to around 529 nm for the JC-1 monomer (green). For the JC-1 aggregate, and excitation of 543 nm was used, and the emission was collected around 590 nm (red).

9. Caspase-3/7 activation assay

A549 cells were seeded in white-walled nontransparent-bottomed 96-well microculture plates at a density of 1.5 × 10^4 cells/well and allowed to incubate overnight to adhere. The cells were then treated with culture medium (negative control, NC), cisplatin, or **Ru4**, respectively. The cells were incubated for 12 h in the dark and divided into two equal groups. The dark group was incubated for an additional 12 h and treated with acaspase-3/7 activity kit (Beyotime Biotechnology, China) according to the manufacturer's protocol. The other group was exposed to laser irradiation (808 nm, 1 W/cm^2) for 5 min, and incubated for an additional 12 h in the dark. The caspase-3/7 activity was determined using an analogous method.

10. Cell cycle analysis

A549 cells (1 × 10^5) were seeded in 6-well plates and incubated overnight. **Ru4** (10 μM) were added into different groups. The dark groups containing **Ru4** were incubated in the dark for 24 h. For the light groups, after incubation with **Ru4** for 12 h, cells were exposed to 808 nm irradiation for 5 min and then allowed to incubate for another 12 h in the dark. After treatment, cells were lysed by RNaseA (100 μg/mL, 37ºC, 20 min) and stained with PI (100 μg/mL, r.t. 15 min) and analyzed *via* flow cytometry (CytoFLEX, Beckman Coulter).

11. *In vitro* NIR-II cell imaging

NIR-II fluorescence images of A549 cells were taken at an exposure time of 100 ms using a NIR-II fluorescence microscope. Excitation was affected at 808 nm using a diode laser with an 80 μm diameter spot focused by a 100 × objective lens (Olympus). The resulting NIR-II fluorescence (FL) was collected using a liquid-nitrogen-cooled, 320 × 256 pixels, two-dimensional InGaAs camera (Princeton Instruments) with sensitivity over the 800 to 1700 nm spectral region. The excitation light was filtered out using a 900 nm long-pass filter and an 1100 nm long-pass filter (both Thorlabs). The NIR FL images were taken at a fixed exposure time of 300 ms. For bright field white-light images, a fiber optic illuminator (Fiber-Lite) was used to illuminate the sample in the trans-illumination mode. Images were recorded using the same filters at a fixed exposure time of 100 ms.

***In Vivo* Anti-tumor Application**

1. Animals and tumor model

All animal experimental procedures were conducted in agreement with the guidelines of the Institutional Animal Care and Use Committee (CCNU-IACUC-2011-058). A549 cells (1×10^6 in 100 μL of PBS) were injected into female nude mice (Suzhou Belda Bio-Pharmaceutical Co.). When tumor volume reached to ~100 mm^3 , the nude mice bearing xenograft A549 tumors were subjected to further experiments.

2. In vivo antitumor activity

Tumor volume and body weight were measured for animals in all experiments. Tumor volume was determined by measuring the tumor in two dimensions with calipers and calculated using the formula tumor volume = (length × width^2)/2. The mice were divided into four groups randomly (n = 5) when the mean tumor volume reached about 100 mm^3 and this day was set as day 0. Mice were administrated intratumorally with PBS (group 1), PBS plus laser (group 2), cisplatin (1 mg Ru/kg) (group 3), **Ru4** (1 mg Ru/kg) (group 4) and **Ru4** (1 mg Ru/kg) plus laser (group 5). Tumor volumes and body weights were measured every 2 days for A549 tumor-bearing mice. 12 h after intratumoral injection, the tumors (group 2 and 5) were illustrated with 808 nm laser (1 W cm^{-2}, 10 min).

3. Histological examination

Upon completion of the PDT treatment, the mice were sacrificed. The tumors and organs including heart, liver, spleen, lung, kidney, intestine and brain were resected, immersed in 4% paraformaldehyde and stored at 4 °C for seven days. The sections of the tumors and organs were obtained as paraffin-embedded samples and stained with hematoxylin and eosin (H&E). Deep blue-purple hematoxylin and pink Eosin

stained nucleic acids and proteins, respectively. A Carl Zeiss Axio Imager Z2 microscope was used to observe the tissue structure and cell state of the sections.

Supplementary Figures

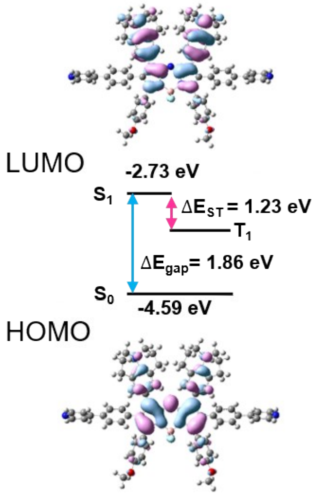

Figure S1. Density functional theory (DFT) calculation of **L**.

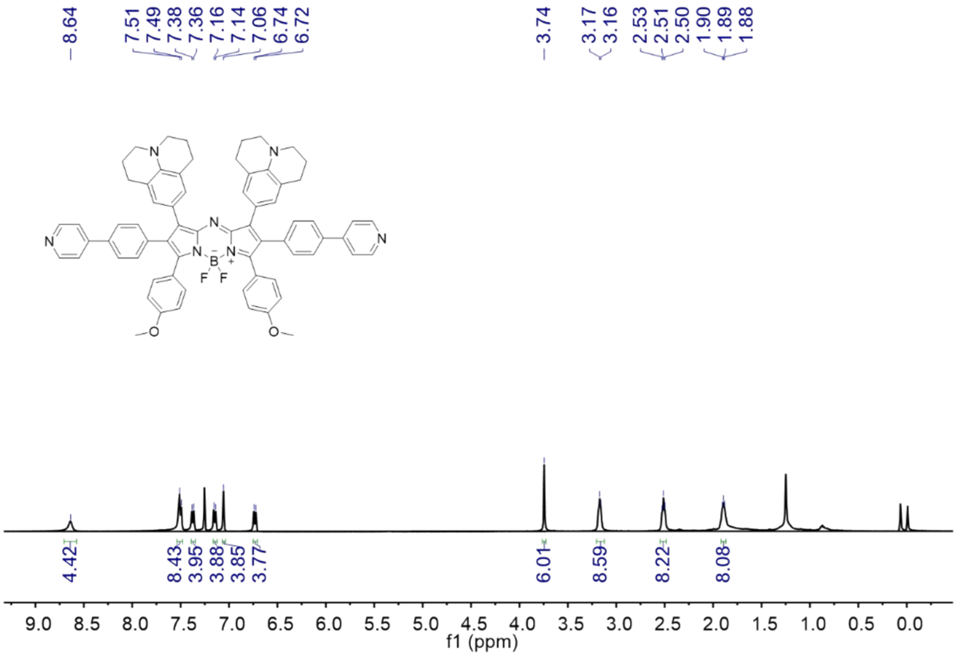

Figure S2. ^{1}H NMR spectrum (400 MHz, $CDCl_3$, 298 K) of **L**.

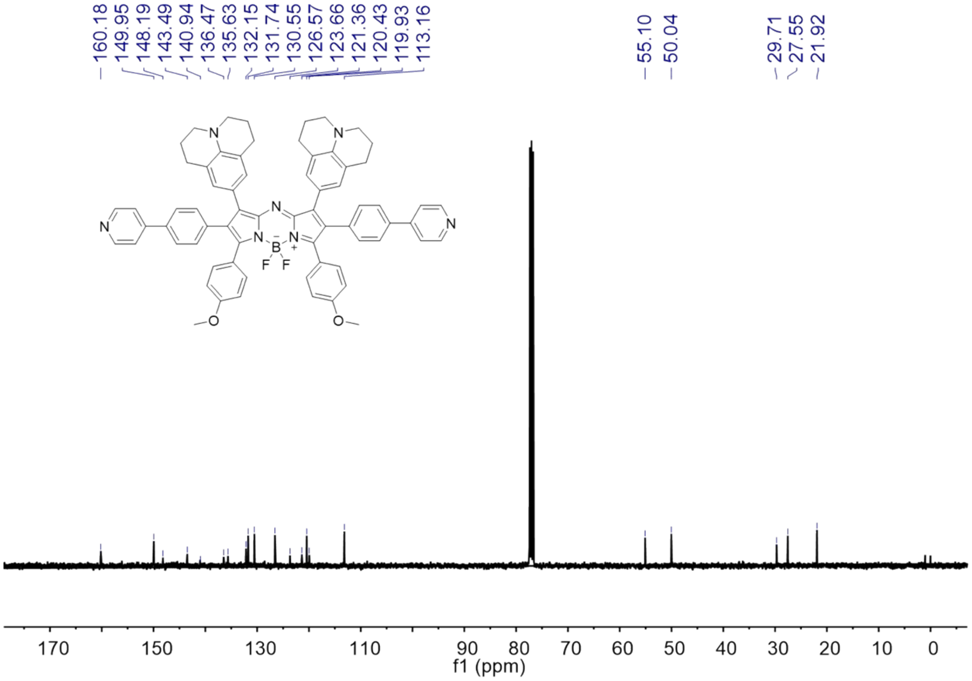

Figure S3. ^{13}C NMR spectrum (100 MHz, $CDCl_3$, 298 K) of **L**.

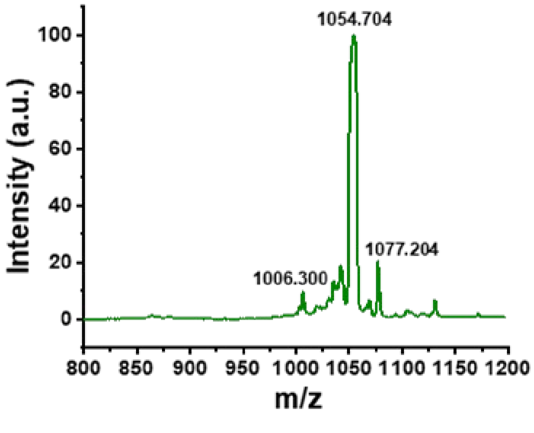

Figure S4. MALDI-TOF-MS of ligand **L**.

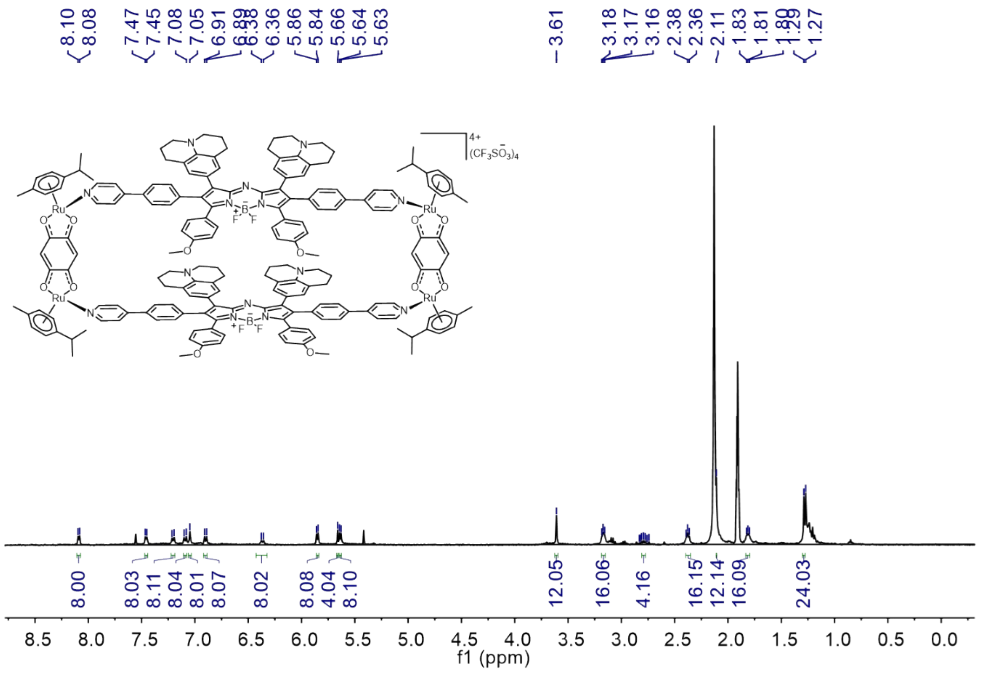

Figure S5. ^{1}H NMR spectrum (400 MHz, CD_3CN, 298 K) of **Ru1**.

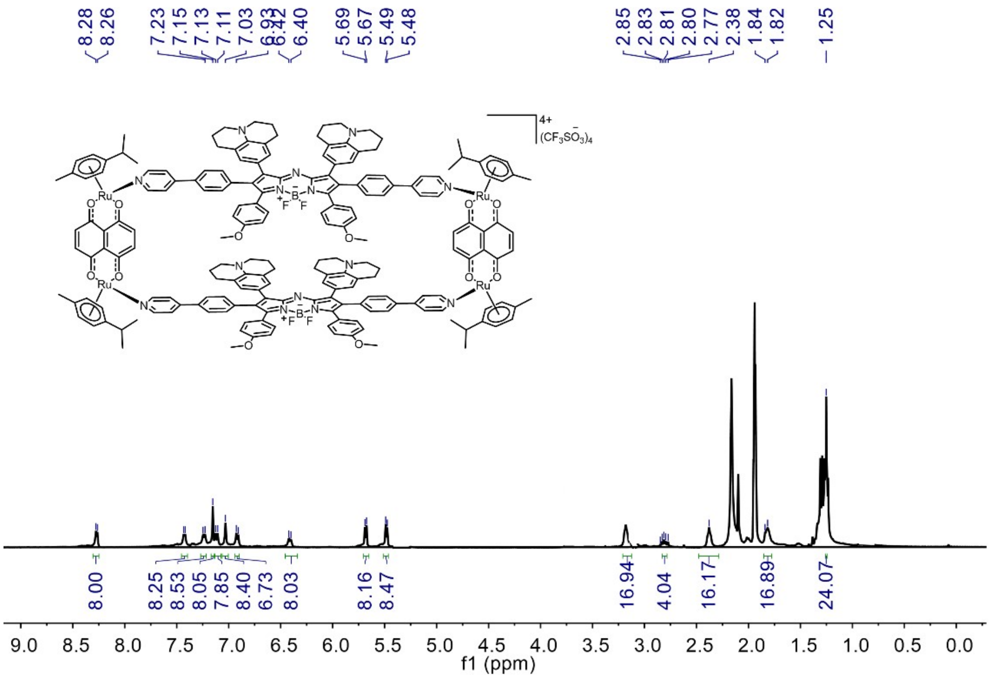

Figure S6. ^{1}H NMR spectrum (400 MHz, CD_3CN, 298 K) of **Ru2**.

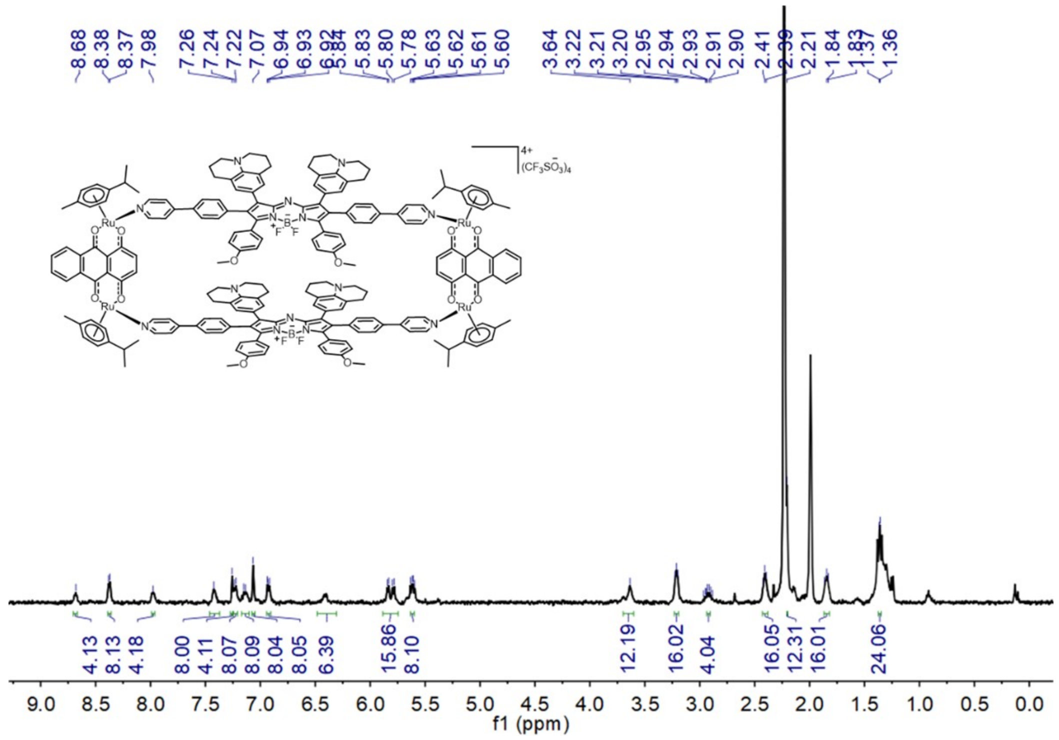

Figure S7. ^{1}H NMR spectrum (400 MHz, CD_3CN, 298 K) of **Ru3**.

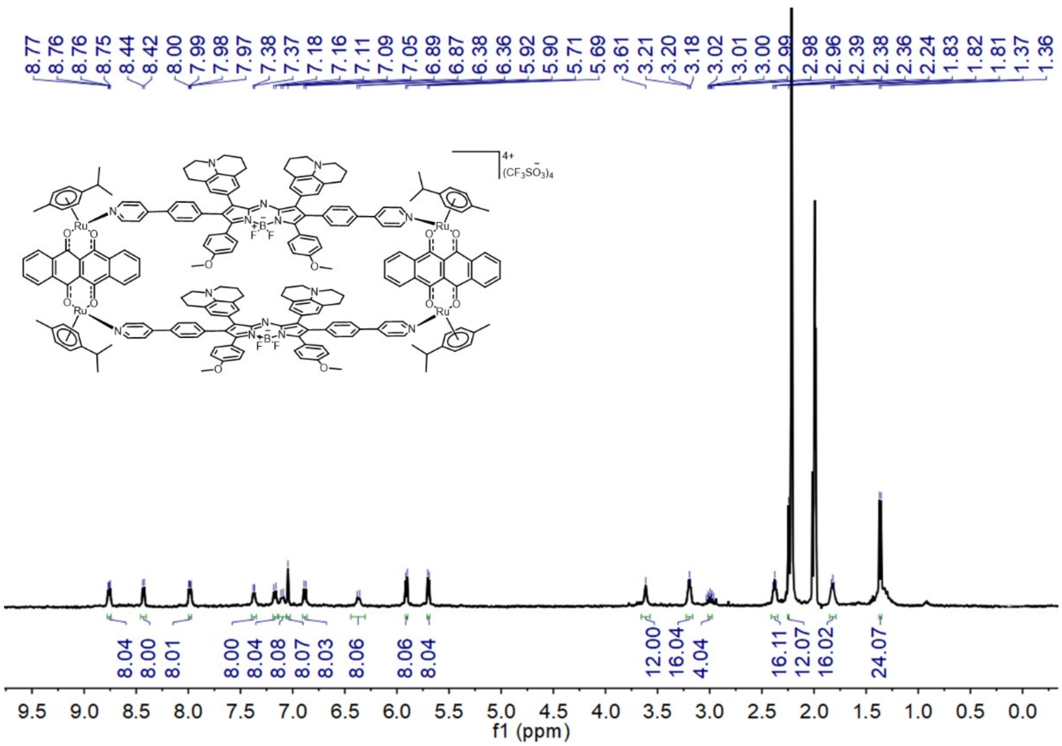

Figure S8. ^{1}H NMR spectrum (400 MHz, CD_3CN, 298 K) of **Ru4**.

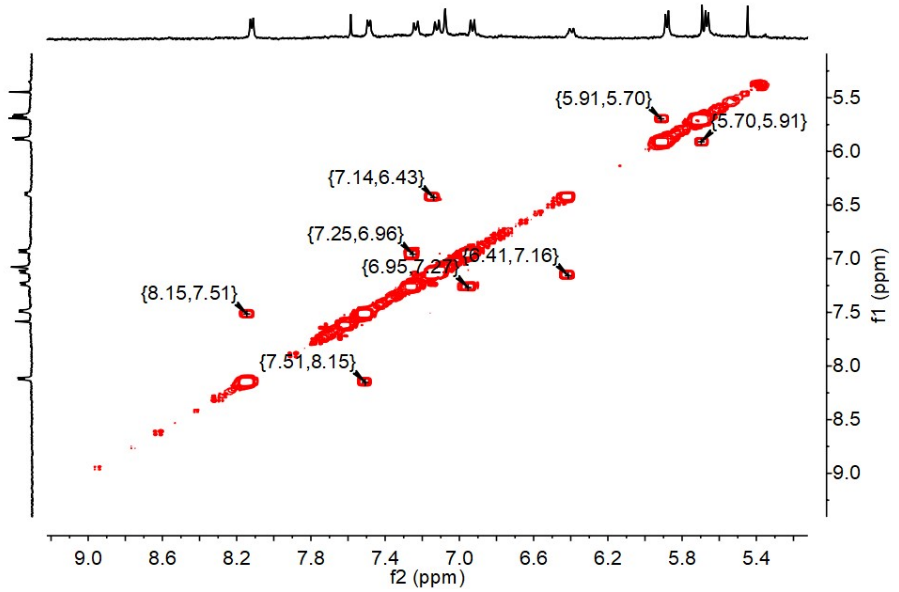

Figure S9. Partial 2D COSY NMR spectrum (400 MHz, CD_3CN, 298 K) of **Ru1**.

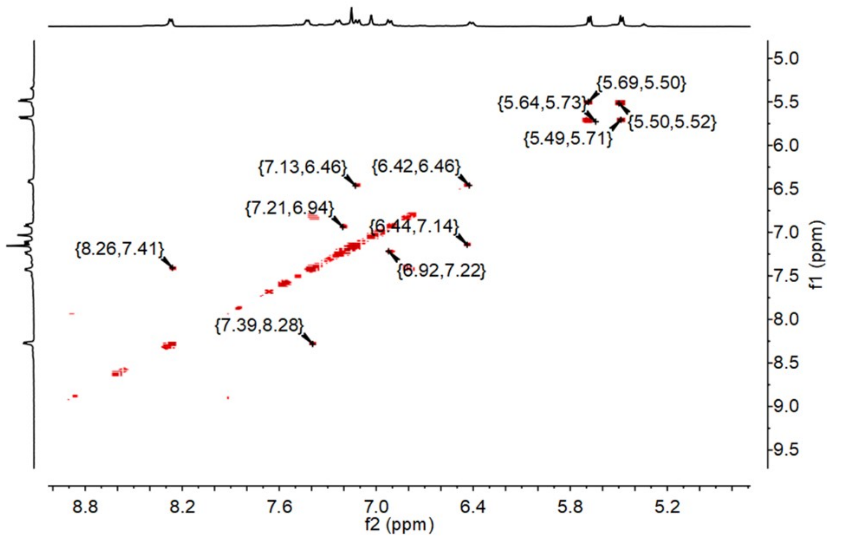

Figure S10. Partial 2D COSY NMR spectrum (400 MHz, CD_3CN, 298 K) of **Ru2**.

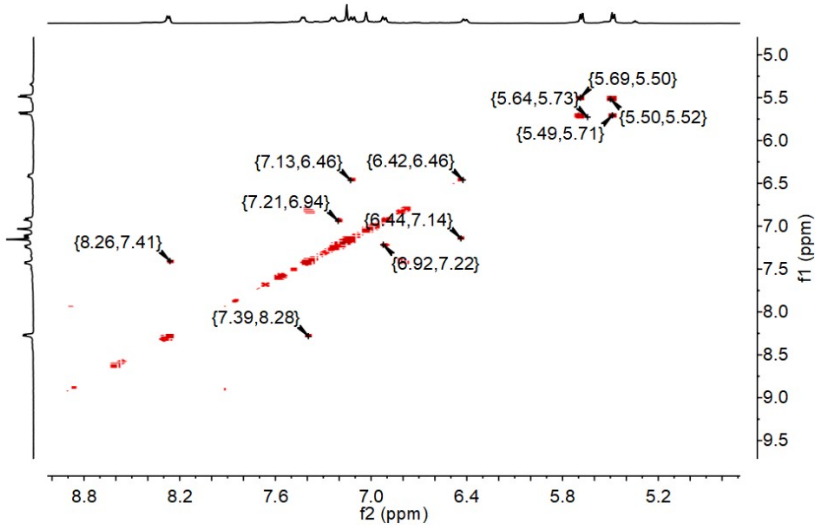

Figure S11. Partial 2D COSY NMR spectrum (400 MHz, CD_3CN, 298 K) of **Ru3**.

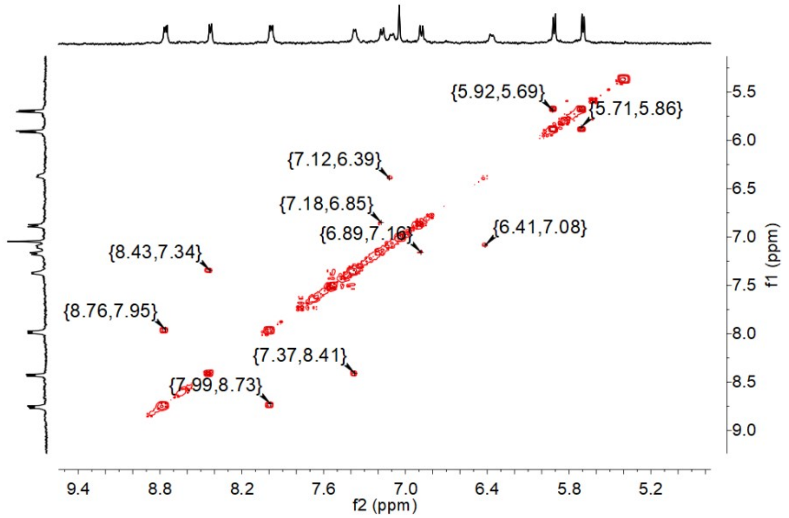

Figure S12. Partial 2D COSY NMR spectrum (400 MHz, CD_3CN, 298 K) of **Ru4**.

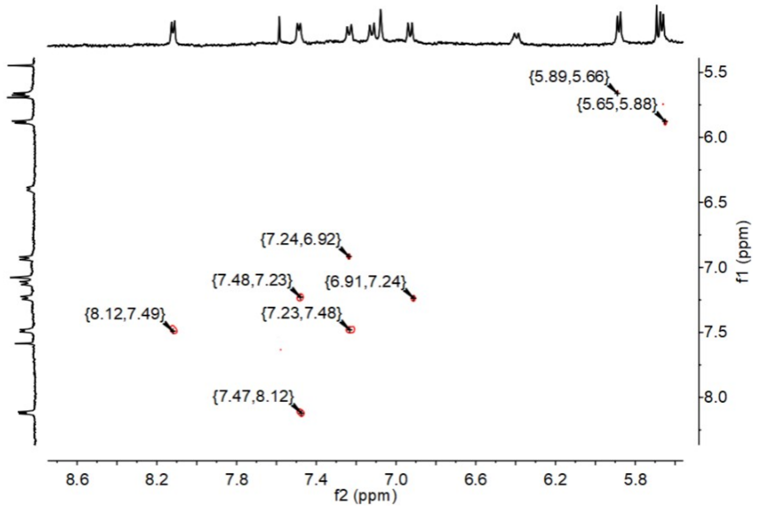

Figure S13. Partial 2D ROESY NMR spectrum (400 MHz, CD_3CN, 298 K) of **Ru1**.

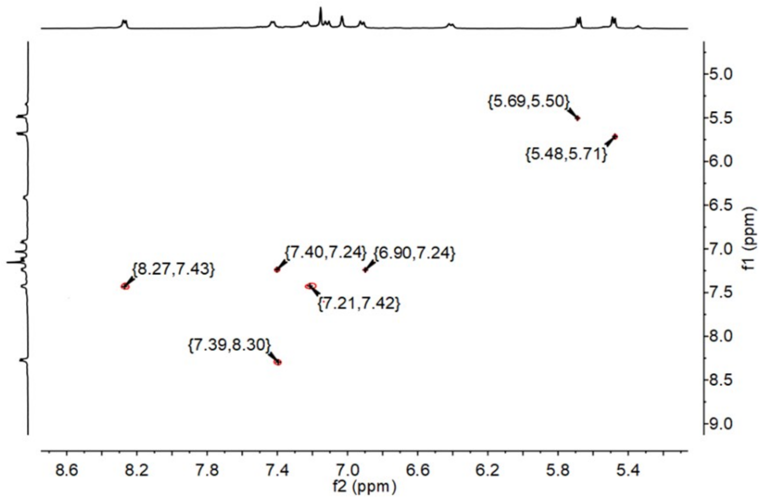

Figure S14. Partial 2D ROESY NMR spectrum (400 MHz, CD_3CN, 298 K) of **Ru2**.

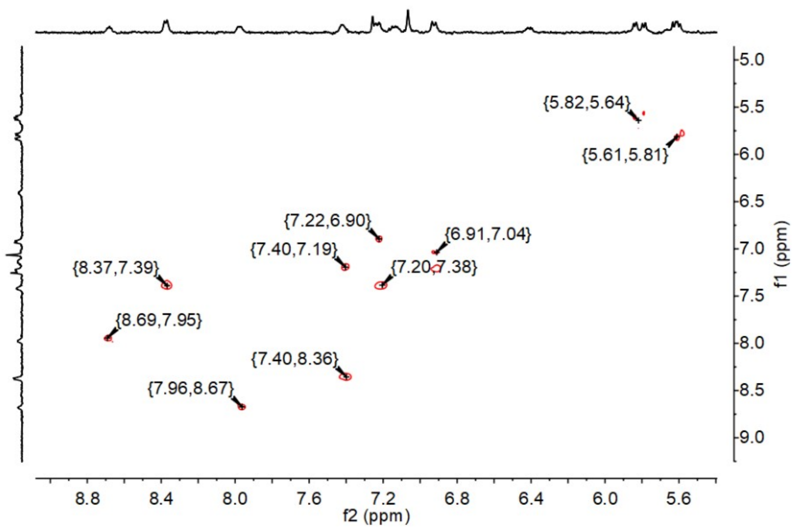

Figure S15. Partial 2D ROESY NMR spectrum (400 MHz, CD_3CN, 298 K) of **Ru3**.

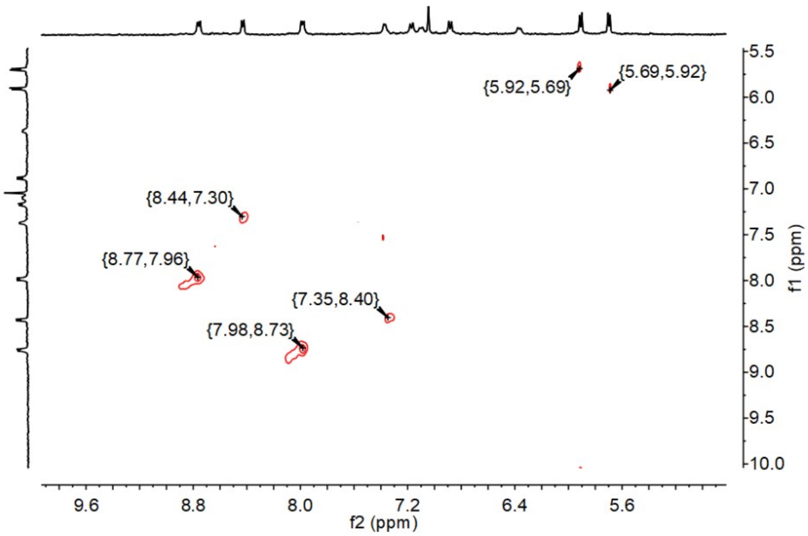

Figure S16. Partial 2D ROESY NMR spectrum (400 MHz, CD_3CN, 298 K) of **Ru4**.

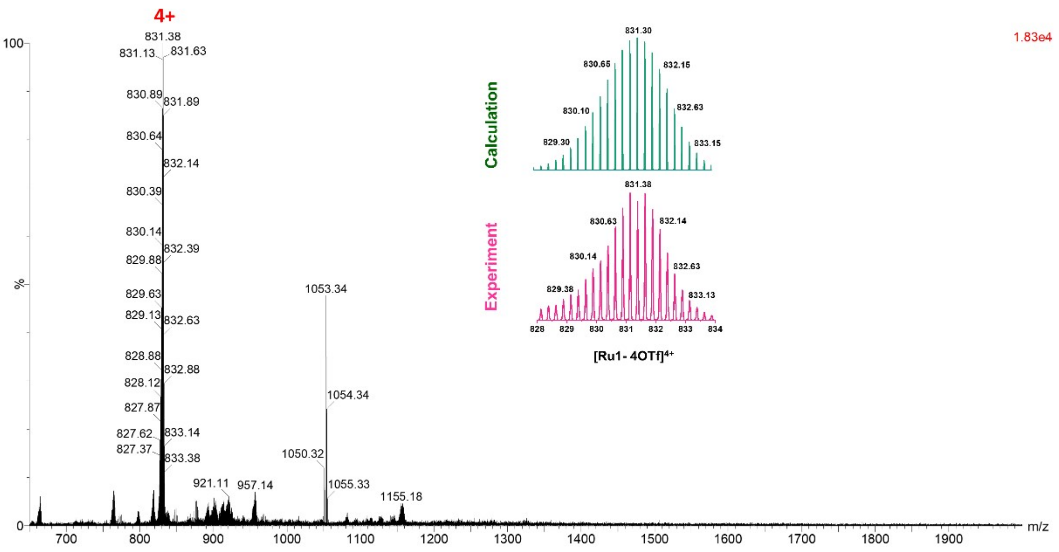

Figure S17. Experimental (magenta) and calculated (green) electrospray ionization mass spectrum of **Ru1**.

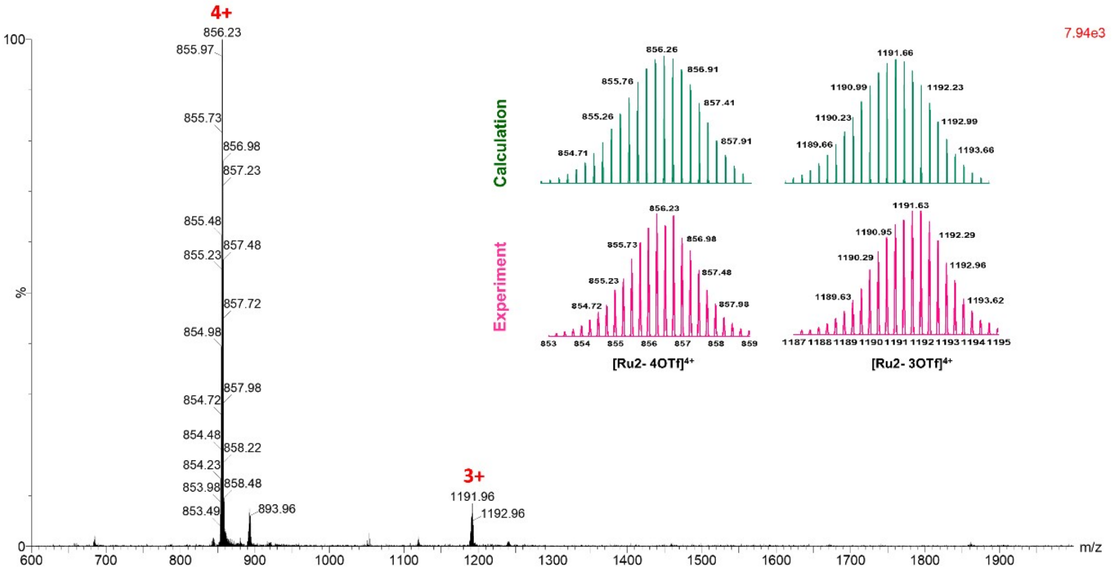

Figure S18. Experimental (magenta) and calculated (green) electrospray ionization mass spectrum of **Ru2**.

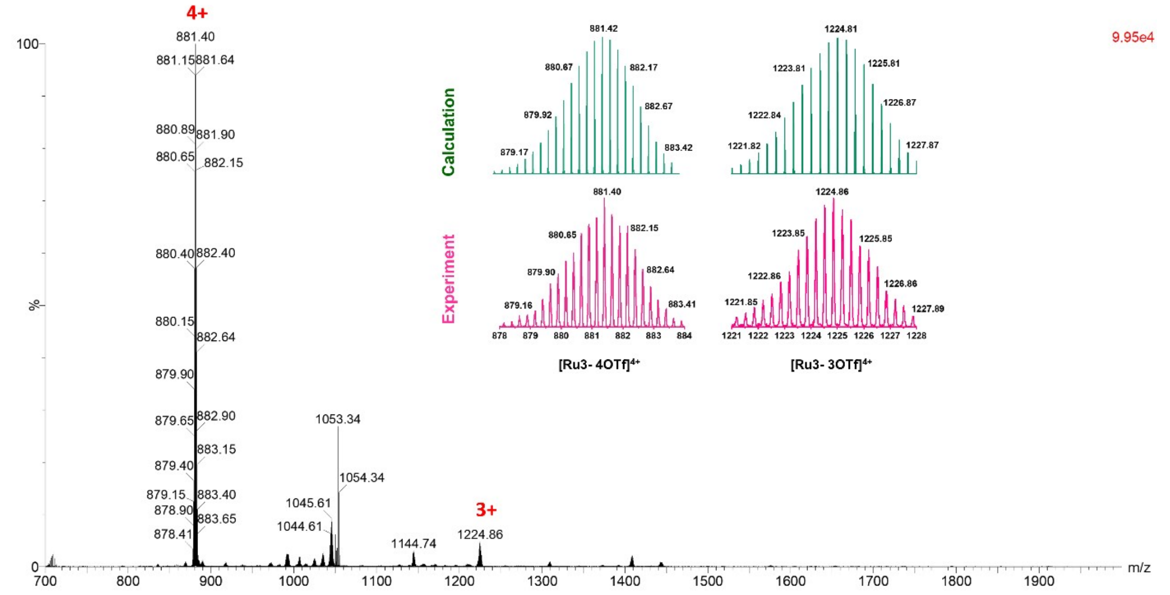

Figure S19. Experimental (magenta) and calculated (green) electrospray ionization mass spectrum of **Ru3**.

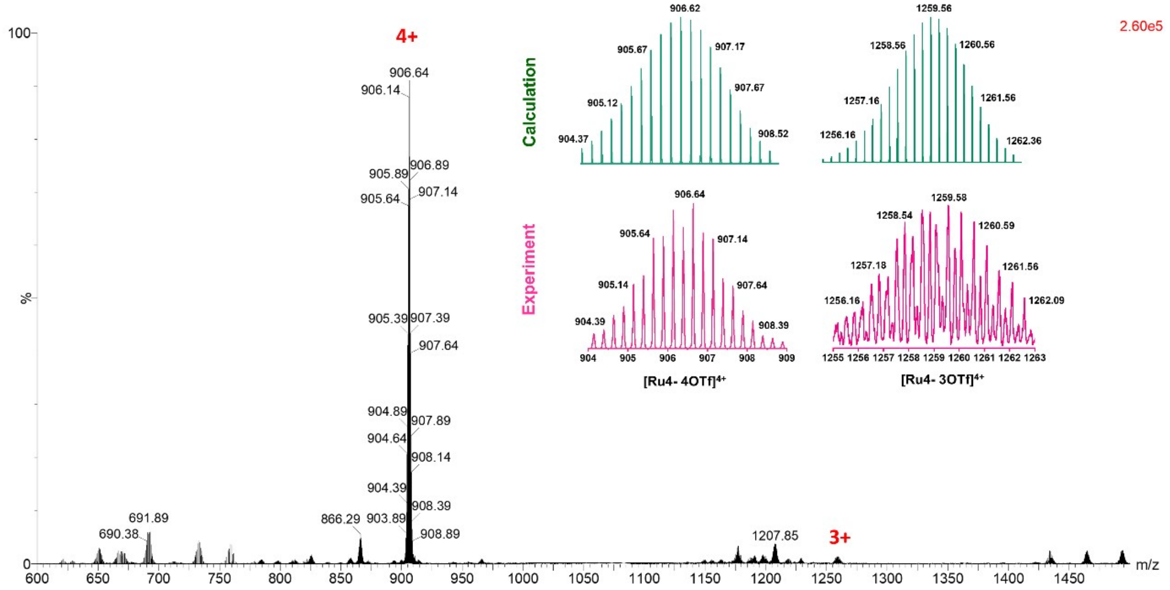

Figure S20. Experimental (magenta) and calculated (green) electrospray ionization mass spectrum of **Ru4**.

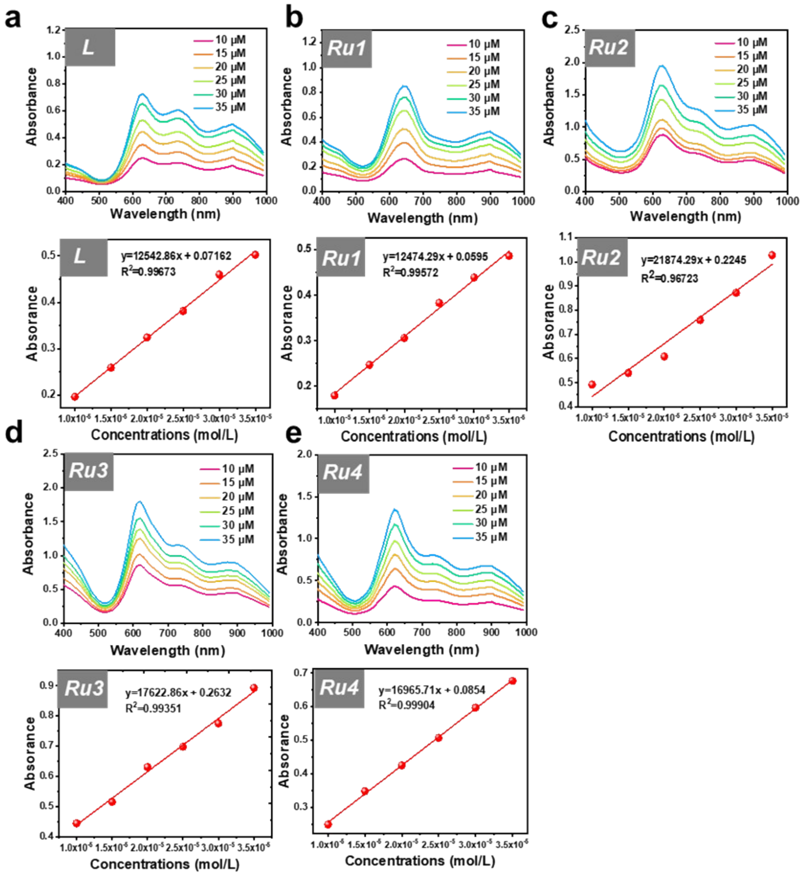

Figure S21. Absorption spectra and linear fitting curves of (a) **L**, (b) **Ru1**, (c) **Ru2**,(d) **Ru3** and (e) **Ru4** at different concentrations in DMSO.

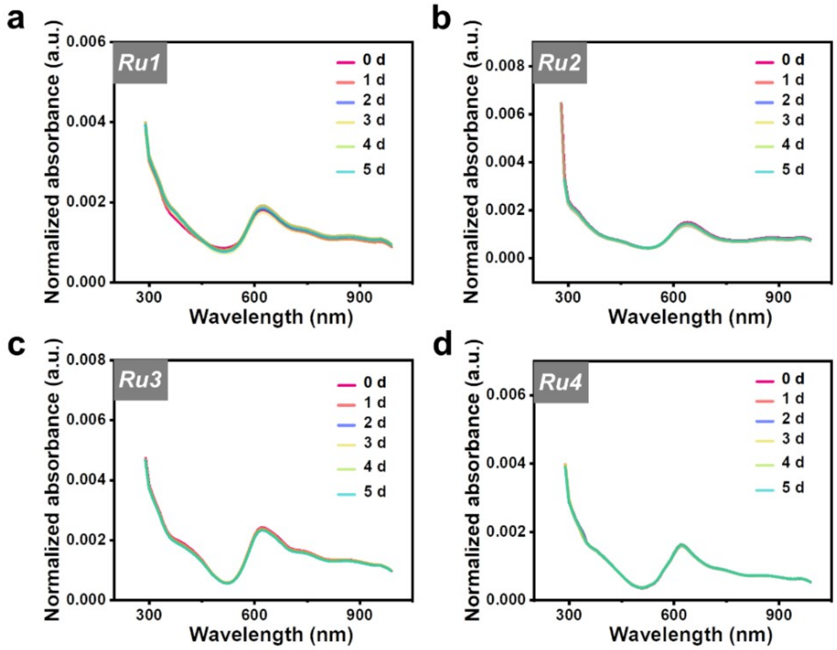

Figure S22. Absorption spectra of (a) **Ru1**, (b) **Ru2**, (c) **Ru3** and (d) **Ru4** incubated in PBS buffer and stored for various time (0, 1, 2, 3, 4 and 5d)

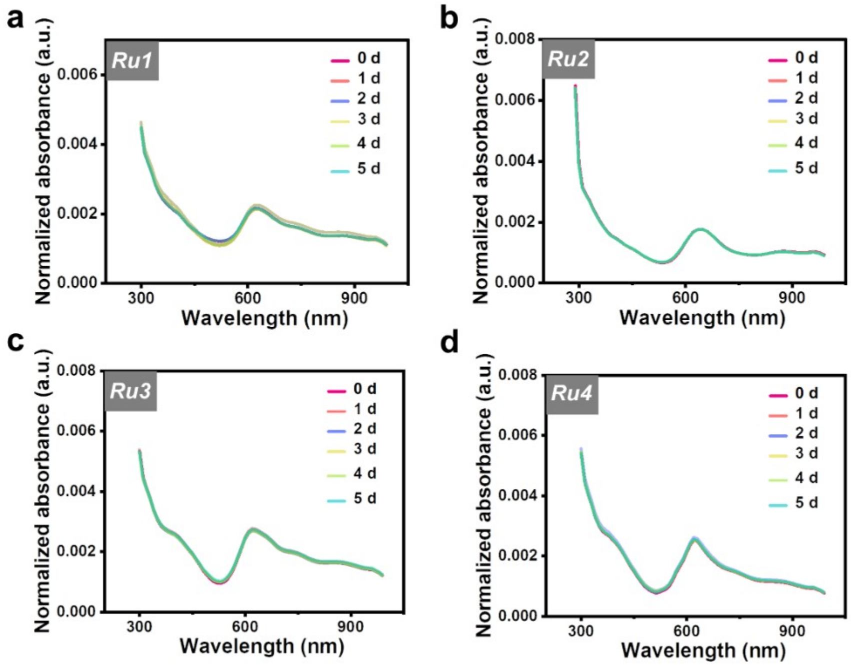

Figure S23. The stability tests of (a) **Ru1**, (b) **Ru2**,(c) **Ru3** and (d) **Ru4** incubated in 10% FBS for various time (0, 1, 2, 3, 4 and 5d).

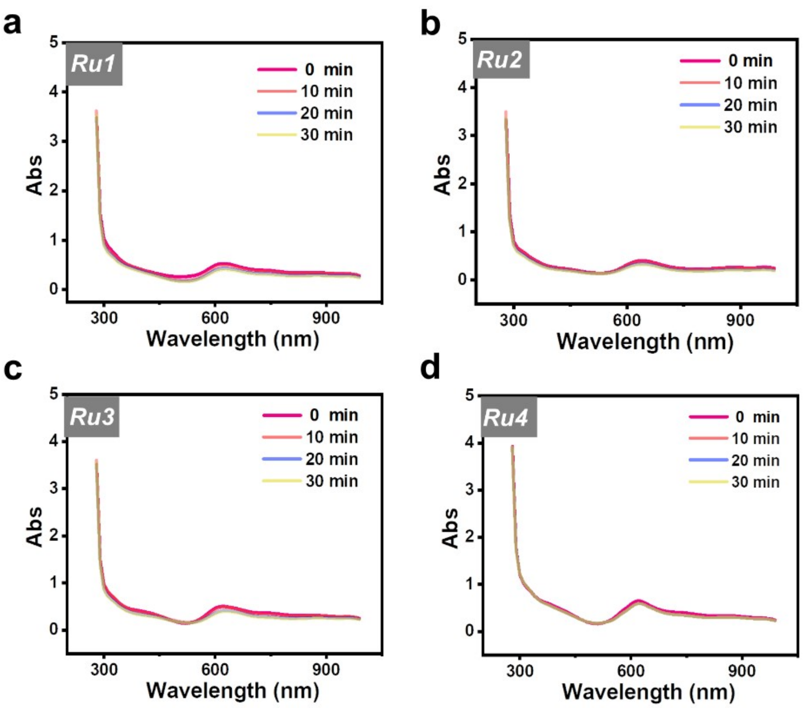

Figure S24. Absorption spectra of (a) **Ru1**, (b) **Ru2**, (c) **Ru3** and (d) **Ru4** in DMSO under 808 nm laser illumination (1 W/cm^2) for various time (0, 10, 20 and 30 min).

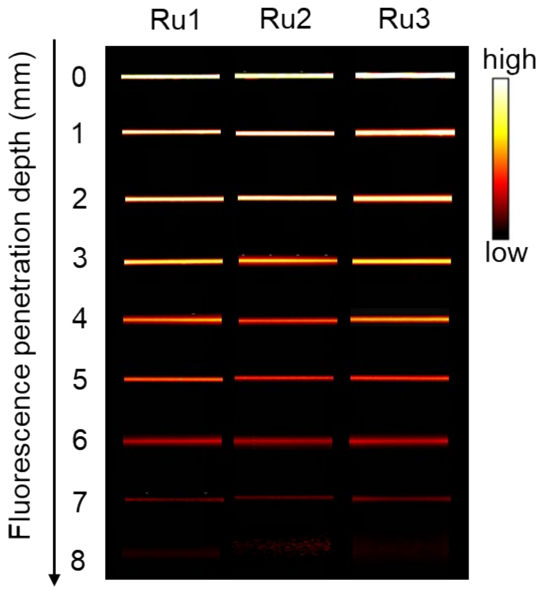

Figure S25. Fluorescence images of **Ru1**-**Ru3** (10 μM) encapsulated in capillaries and immersed at varied depths in 1% intralipid.

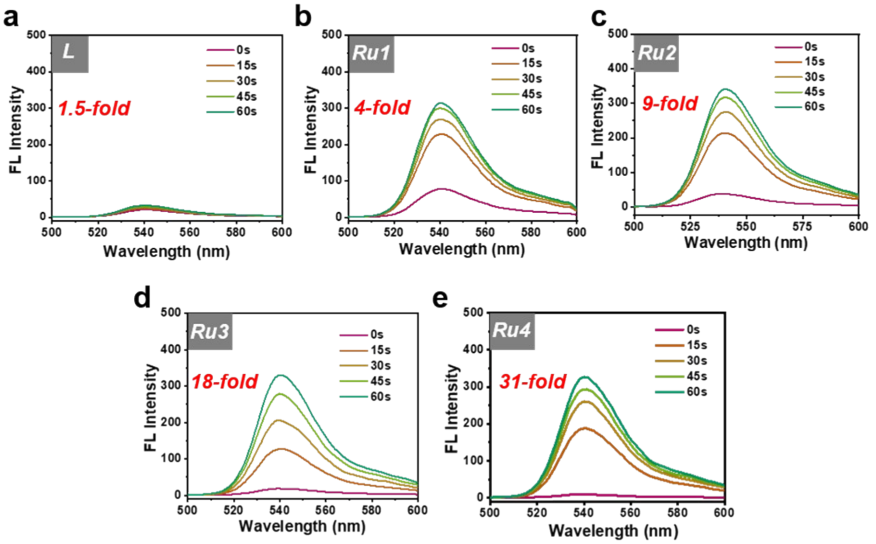

Figure S26. The DCF fluorescent spectra of (a) **L**, (b) **Ru1**, (c) **Ru2**, (d) **Ru3** and (e) **Ru4** under 808 nm laser irradiation (1 W/ cm^2) recorded at different time points, the concentration of **Ru1-Ru4** is 10 μM.

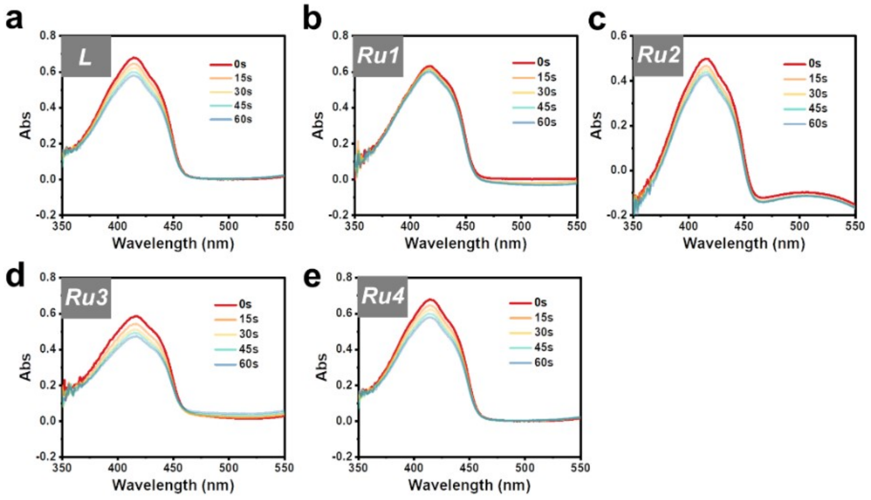

Figure S27. Absorption spectra of DPBF ($^1O_2$ probe, 10 μM) recorded after different irradiation times in the presence of (a) **L**, (b) **Ru1**, (c) **Ru2**, (d) **Ru3** and (e) **Ru4**, the concentration of **Ru1-Ru4** is 10 μM.

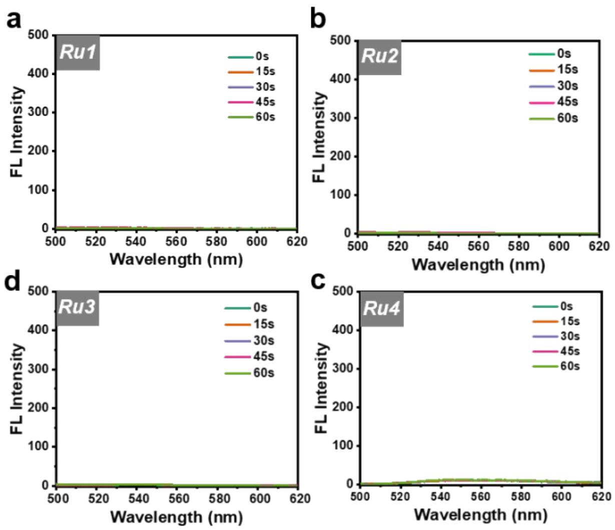

Figure S28. Fluorescence emission spectra of HPF (OH• probe, 10 μM) recorded after different irradiation times in the presence of (a) **Ru1**, (b) **Ru2**, (c) **Ru3** and (d) **Ru4**, the concentration of **Ru1-Ru4** is 10 μM.

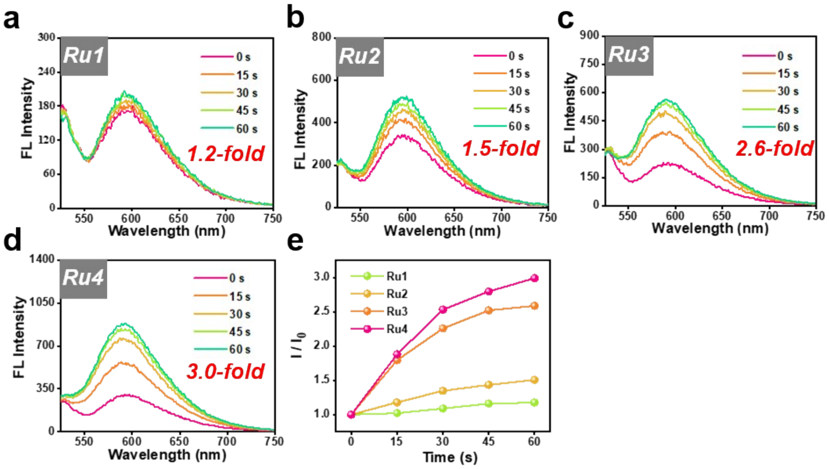

Figure S29. $O_2^{-\bullet}$ detection using the DHE (10 μM) assay for (a) **Ru1**, (b) **Ru2**, (c) **Ru3** and (d) **Ru4** in aqueous solution. (e) Comparison of fluorescence intensity for $O_2^{-\bullet}$ using DHE as fluorescence probe of and **Ru1-Ru4** (10 μM).

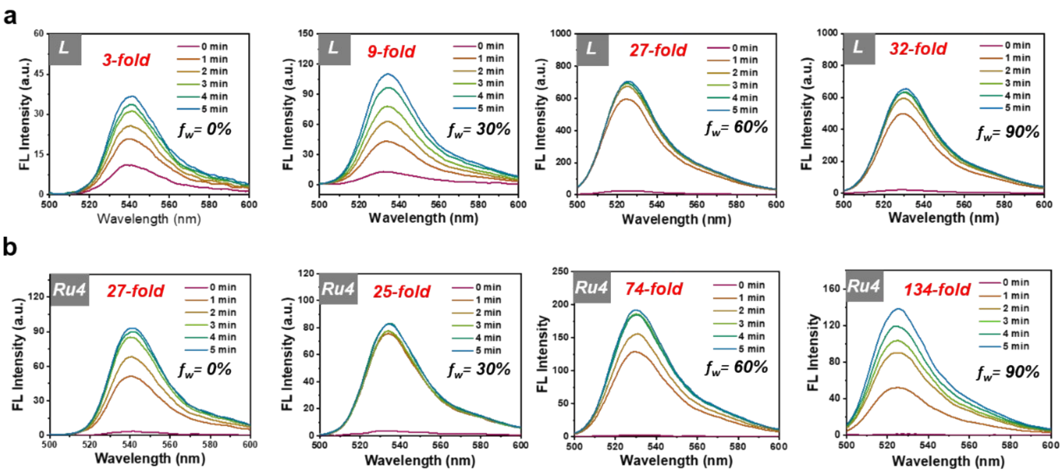

Figure S30. The DCF fluorescent spectra of (a) **L** (20 μM) and (b) **Ru4** (10 μM) under 808 nm laser irradiation (1 W/cm^2) in DMSO/water mixtures with different water fractions (f_w), respectively.

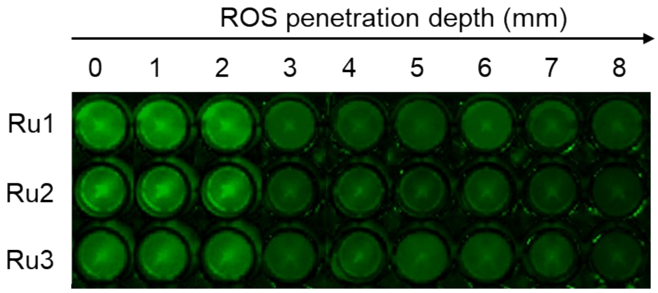

Figure S31. The fluorescence imaging of **Ru1**-**Ru3** (10 μM) simulated tissues to detect the depth-activated ROS generation using DCFH-DA as ROS probe under 808 nm laser irradiation for 60 s (1 W/cm^2).

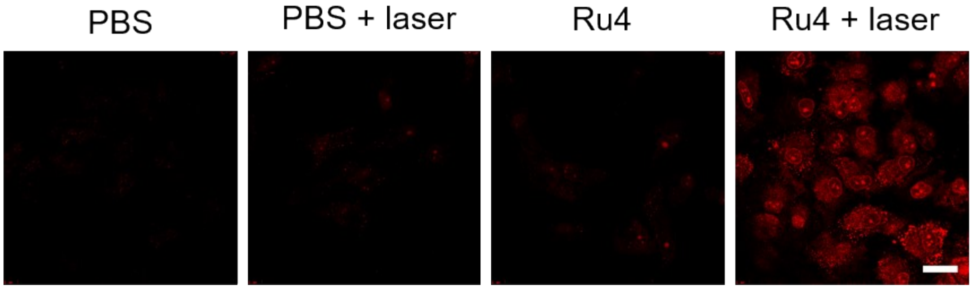

Figure S32. Confocal fluorescence images of **Ru4** (10 μM)-treated A549 cells in the presence of DHE with or without laser irradiation (808 nm, 1 W/cm^2). Scale bars: 20 μm.

Table S1. IC_{50} value of **Ru1-Ru4** (μM) and cisplatin against different cell lines and under normoxia/hypoxia conditions

Cell	Dark/Light	Cisplatin	Ru1	Ru2	Ru3	Ru4
A549	Dark	16.7 ± 1.3	214.8 ± 2.9	18.0 ± 3.5	89.0 ± 2.4	305.6 ± 29.5
	Light	17.4 ± 2.0	10.7 ± 0.9	15.6 ± 4.1	19.9 ± 1.1	3.6 ± 0.3
	PI[a]	1.0	20.0	1.1	4.5	85.8
A549/DDP	Dark	55.9 ± 5.6	105.3 ± 5.3	21.9 ± 2.0	125.4 ± 5.5	322.1 ± 28.5
	Light	52.7 ± 3.2	11.8 ± 0.9	14.1 ± 1.7	9.9 ± 0.5	11.7 ± 0.4
	PI[a]	1.1	9.0	1.5	12.7	27.6
A549/ hypoxia	Dark	50.2 ± 4.1	272.2 ± 9.7	22.1 ± 2.6	87.8 ± 13.8	178.1 ± 16.2
	Light	51.9 ± 5.2	8.0 ± 3.1	7.6 ± 1.2	13.4 ± 0.2	1.2 ± 0.3
	PI[a]	1.0	34.2	2.9	6.5	146.0
16HBE	Dark	7.6 ± 1.0	202.6 ± 34.6	23.9 ± 2.0	67.5 ± 1.6	162.4 ± 7.8
	Light	7.3 ± 0.4	14.7 ± 1.6	15.8 ± 2.4	17.8 ± 3.7	5.1 ± 1.2
	SI[b]	0.4	1.4	1.0	0.9	2.7

[a]PI (Photocytotoxicity Index) = $IC_{50(dark)}/IC_{50(light)}$, [b]SI (Selectivity Index) is defined as $IC_{50(light)}$ in 16HBE/$IC_{50(light)}$ in A549.

Table S2. IC_{50} value of **L** and **A1-A4** (μM) in A549 cell

Cell	Dark/Light	L	A1	A2	A3	A4
A549	Dark	104.9 ± 10.1	34.8 ± 2.7	18.2 ± 4.0	53.7 ± 8.4	84.5 ± 9.3
	Light	47.3 ± 11.7	–	–	–	–
	PI[a]	2.2	–	–	–	–

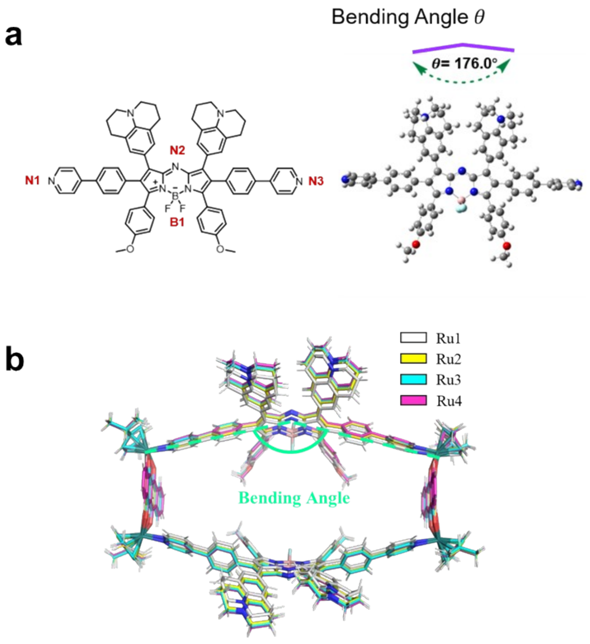

Figure S33. (a) Optimal configuration and bending angle of ligand **L** and (b) **Ru1-Ru4**

Table S3. The octanol/water partition coefficient (log $P_{o/w}$) of **Ru1-Ru4** and **L**

	Ru1	Ru2	Ru3	Ru4
LUMO+5	-9.25977	-8.94765	-8.88697	-8.83663
LUMO+4	-9.80263	-9.69842	-9.66603	-9.63746
LUMO+3	-9.80699	-9.70195	-9.6693	-9.63964
LUMO+2	-10.4364	-10.3237	-9.97434	-9.64699
LUMO+1	-10.4372	-10.3237	-9.97434	-9.65052
LUMO	-11.148	-11.0505	-11.0187	-11.0016
HOMO	-12.6254	-12.4174	-12.3839	-12.2651
HOMO-1	-12.4639	-12.3608	-12.4128	-12.3121
HOMO-2	-12.4688	-12.3659	-12.4171	-12.317
HOMO-3	-12.6111	-12.5126	-12.4838	-12.4664
HOMO-4	-12.6198	-12.5197	-12.4903	-12.4751
HOMO-5	-12.6955	-12.5915	-12.5804	-12.5246

Table S4. The octanol/water partition coefficient (log $P_{o/w}$) of **Ru1-Ru4** and **L**

Compound	Ru 1	Ru 2	Ru 3	Ru 4	L
**Log（$P_{o/w}$）**	1.27	1.47	1.28	1.60	1.71

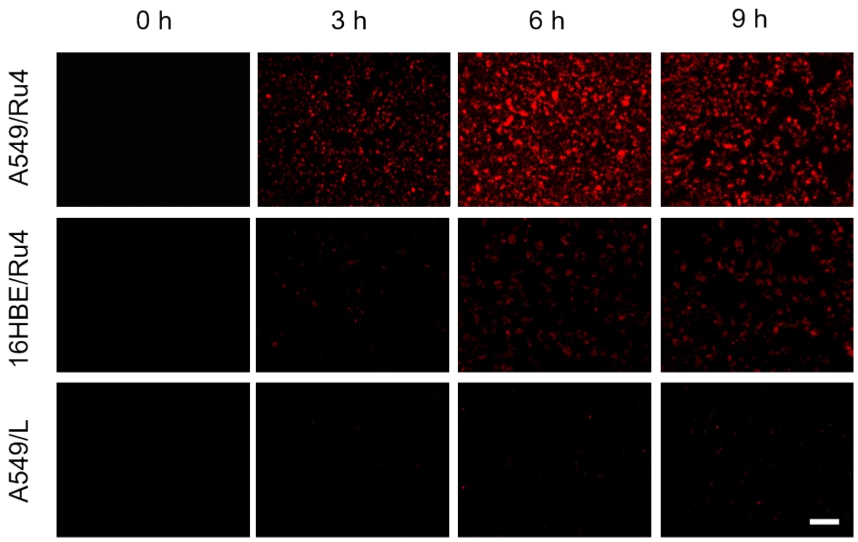

Figure S34. NIR-II fluorescence images of A549 cells incubated with **Ru4** (10 μM) and **L** (20 μM), respectively and 16HBE cells incubated with **Ru4** at different time points. Scale bars = 300 μm.

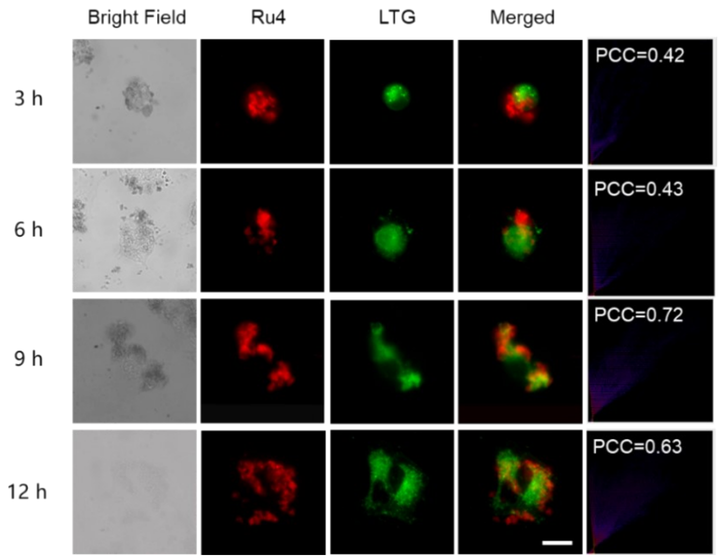

Figure S35. Colocalization images of **Ru4** (10 μM) and LysoTracker® Green (LTG) at different time points with the corresponding correlation coefficients. Scale bars = 10 μm.

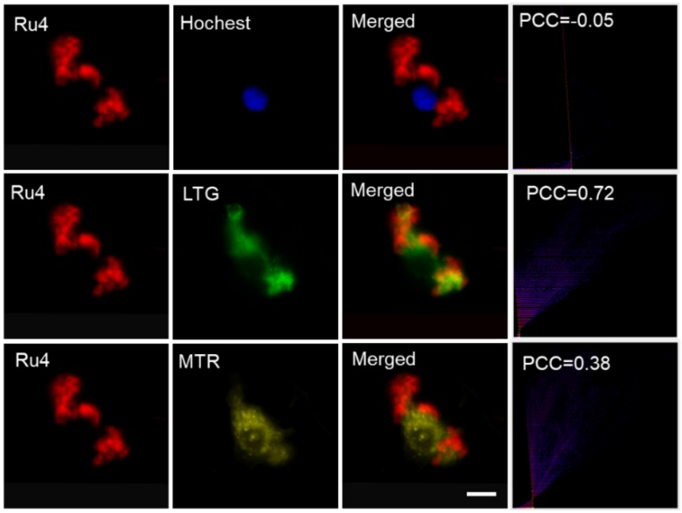

Figure S36. Co-localization assay of **Ru4** (10 μM) incubated with A549 cells for 9 h using LysoTracker Green as lysosomal dye, Mito-Tracker® Red CMXRos as mitochondrial dye and Hoechst 33342 as nuclear dye. The Pearson correlation coefficients (PCC) were analyzed by ImageJ. Scale bars = 10 μm.

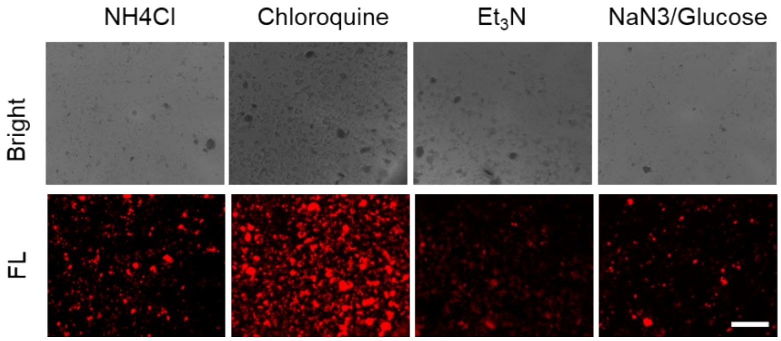

Figure S37. Cell uptake mechanism study of **Ru4** (10 μM) in the presence of different inhibitors/conditions. Scale bars=300 μm.

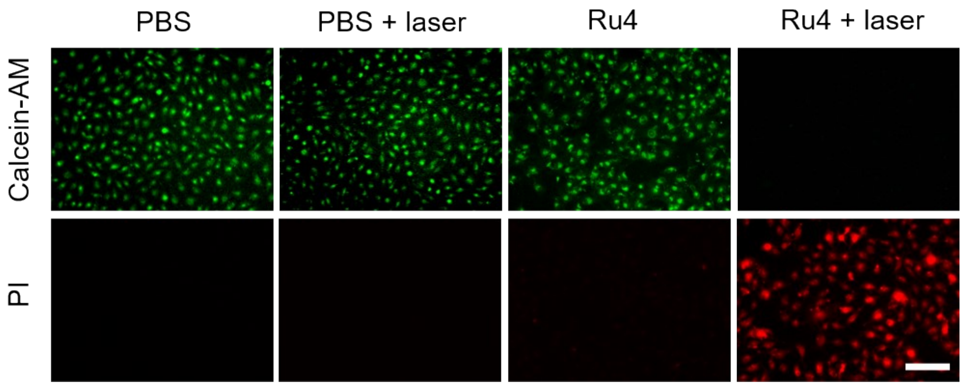

Figure S38. Confocal fluorescence images of live (green) and dead (red) A549 cells with Calcein-AM and PI staining after various treatments. Scale bar: 300 μm.

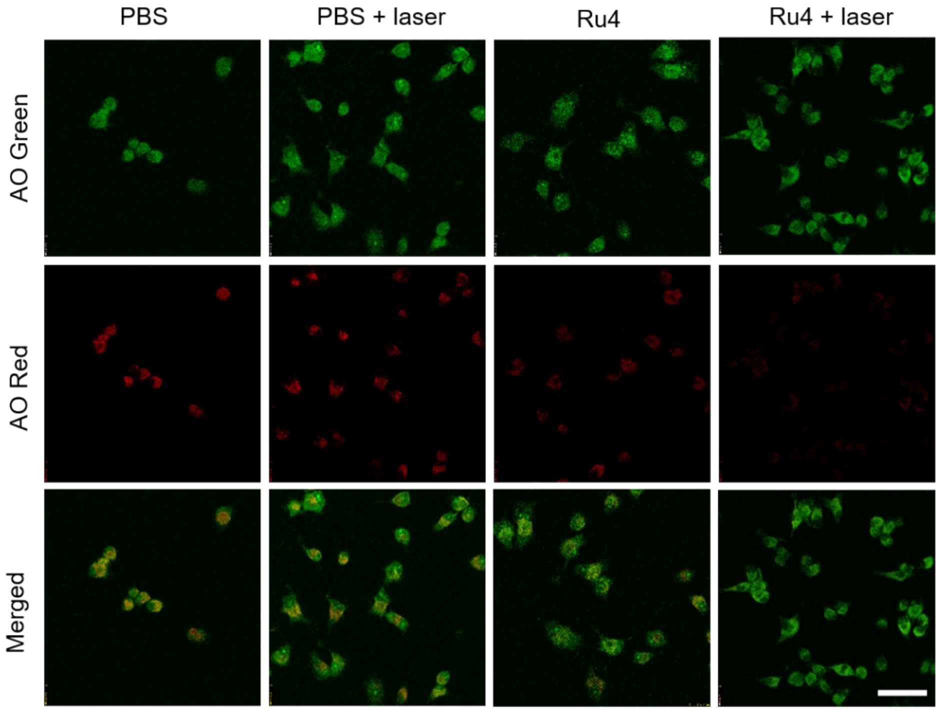

Figure S39. After incubation with **Ru4** (10 μM) or serum-free medium (control) and treated with or without 808 nm laser illumination (1 W/cm^2, 5 min), the A549 cells were taken AO staining. Scale bar =20 μm.

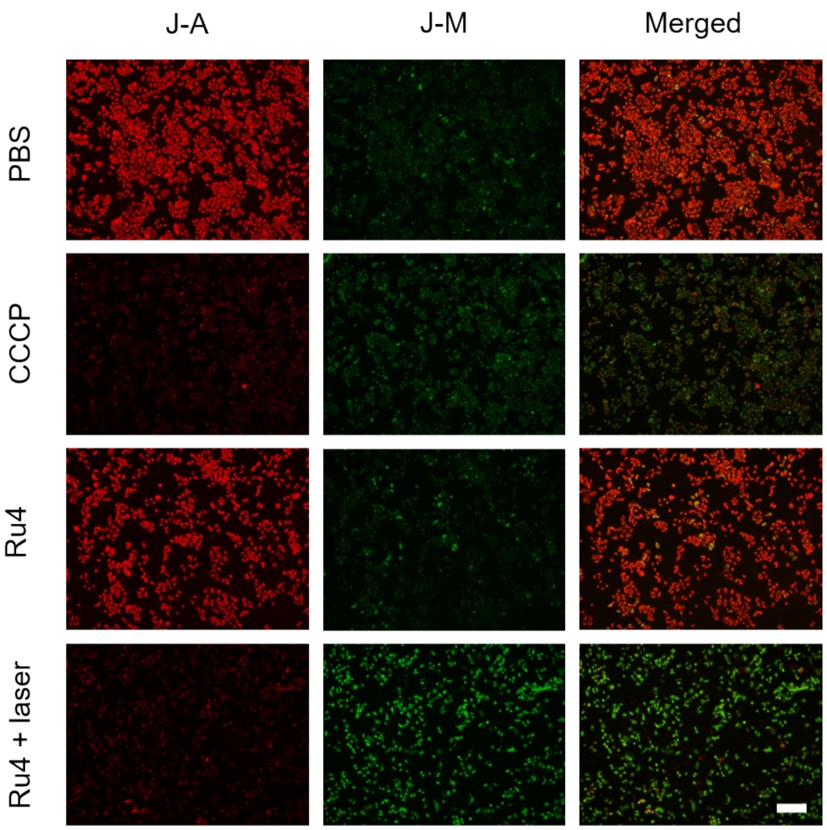

Figure S40. After incubation with **Ru4** (10 μM) or serum-free medium (control) and treated with or without 808 nm laser illumination (1 W/ cm^2, 5 min), the A549 cells were taken JC-1 staining. Carbonyl cyanide m-chlorophenylhydrazone (CCCP) as positive control. Scale bar = 300 μm.

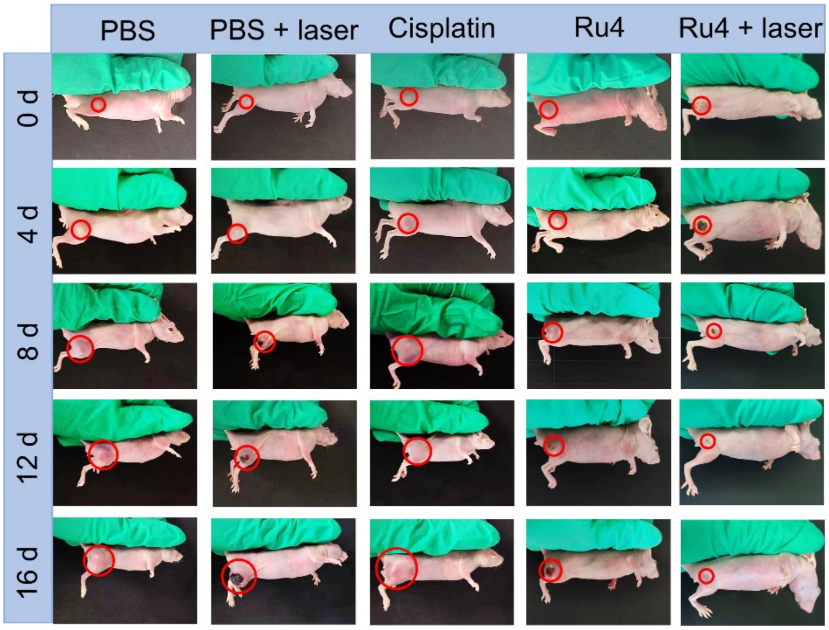

Figure S41. Bright images of mice treated with the indicated formulations at 0, 4, 12 and 16 days.

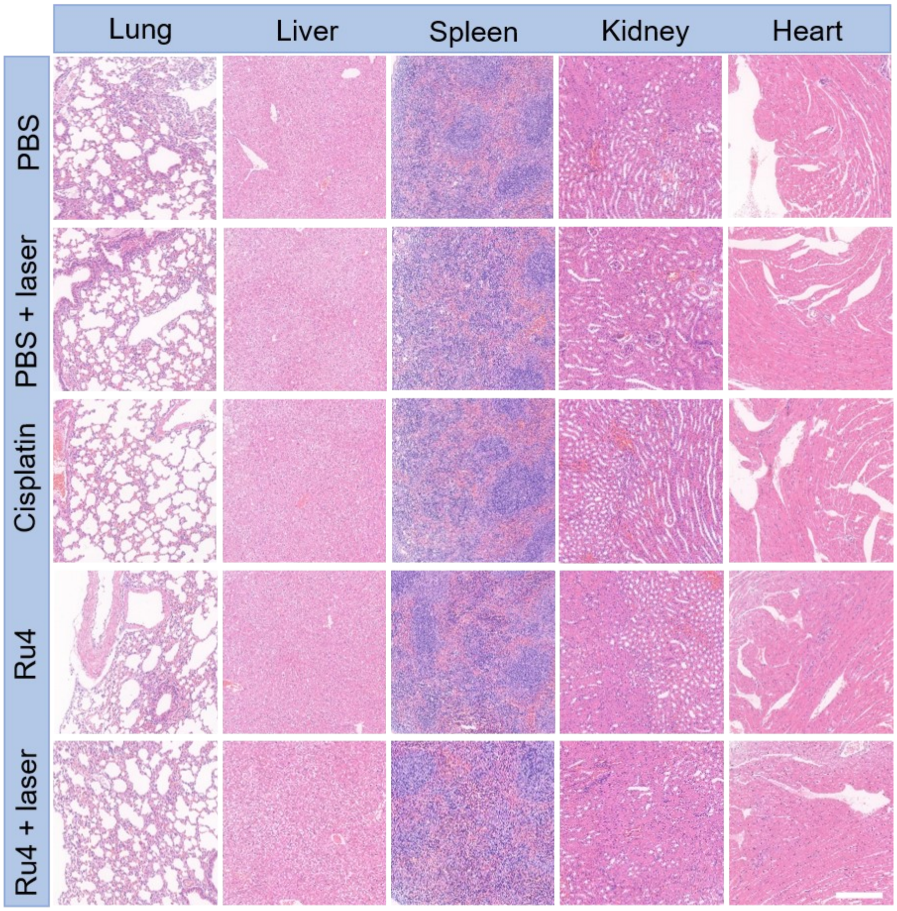

Figure S42. H&E staining of major organs including the lungs, liver, spleen, kidneys and heart of mice treated with different formulations (PBS, PBS + laser, **Ru4** and **Ru4** + laser). Scale bar: 50 μm.

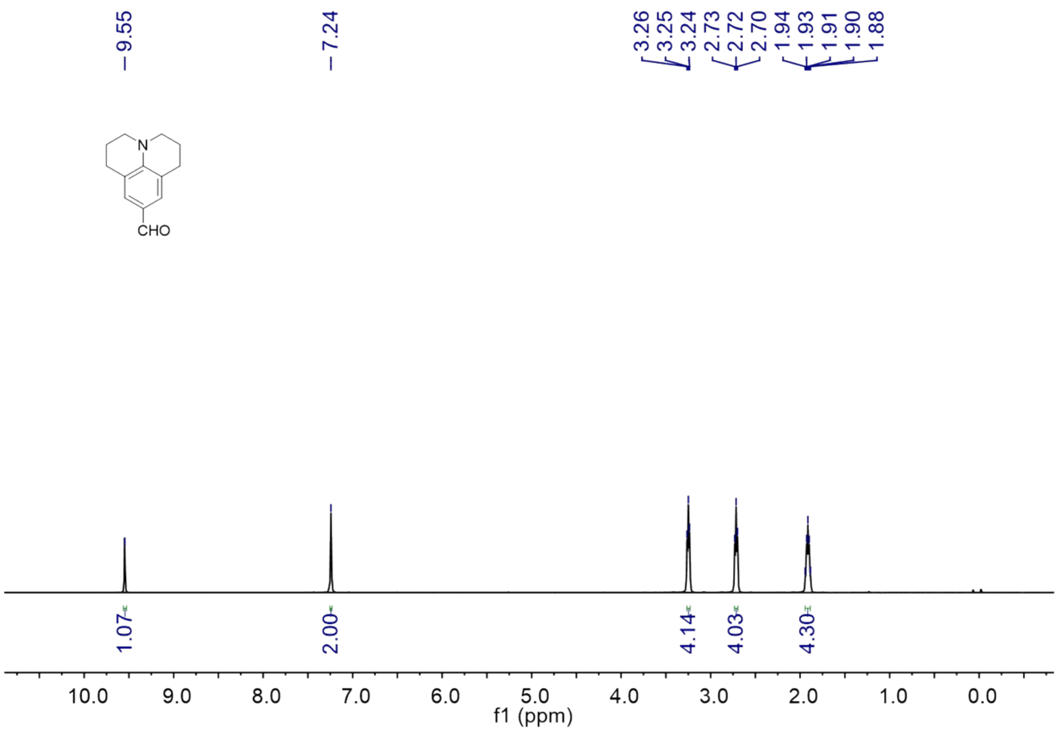

Figure S43. ^{1}H NMR spectrum (400 MHz, $CDCl_3$, 298 K) of **2a**.

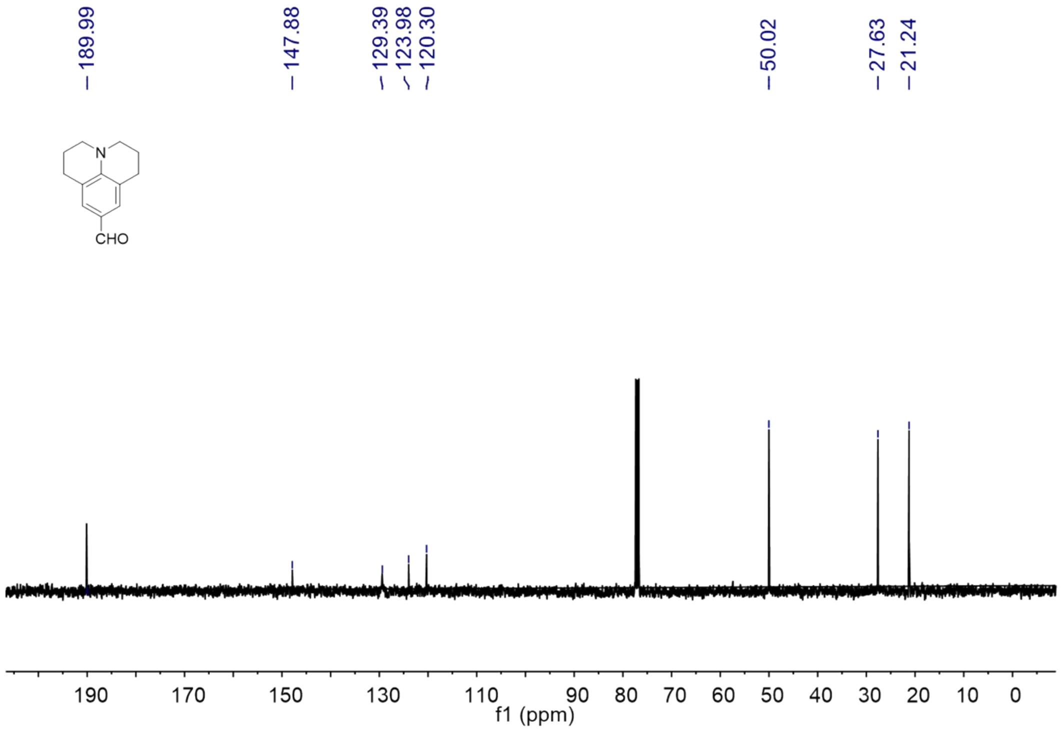

Figure S44. ^{13}C NMR spectrum (100 MHz, $CDCl_3$, 298 K) of **2a**.

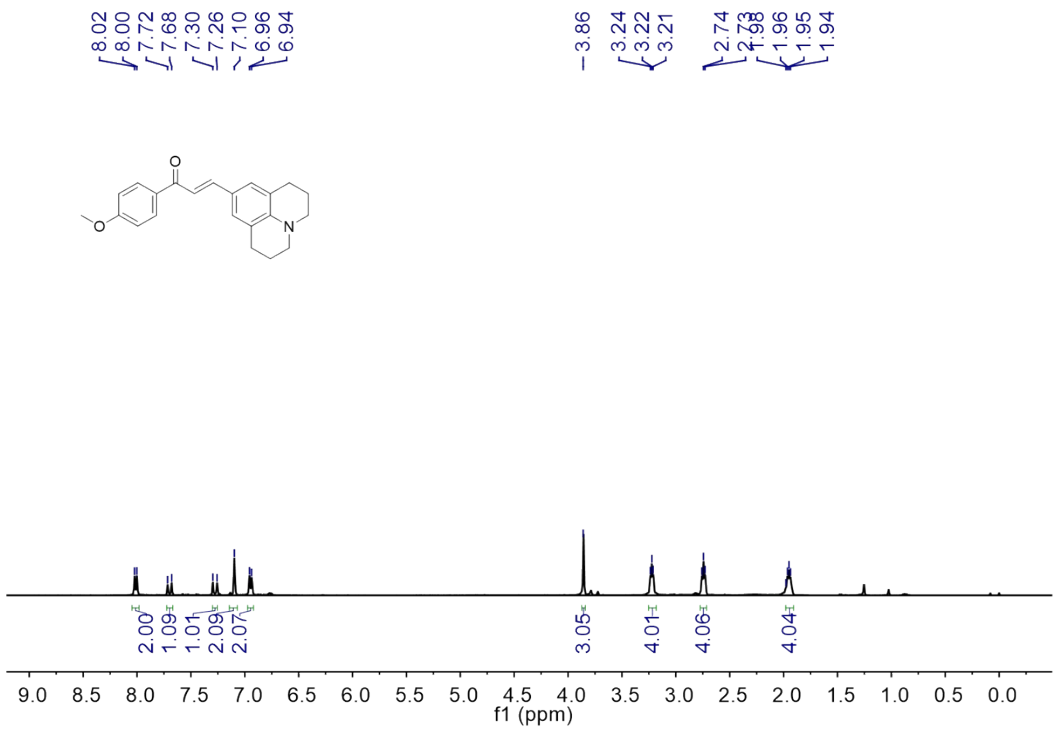

Figure S45. ^{1}H NMR spectrum (400 MHz, $CDCl_3$, 298 K) of **4a**.

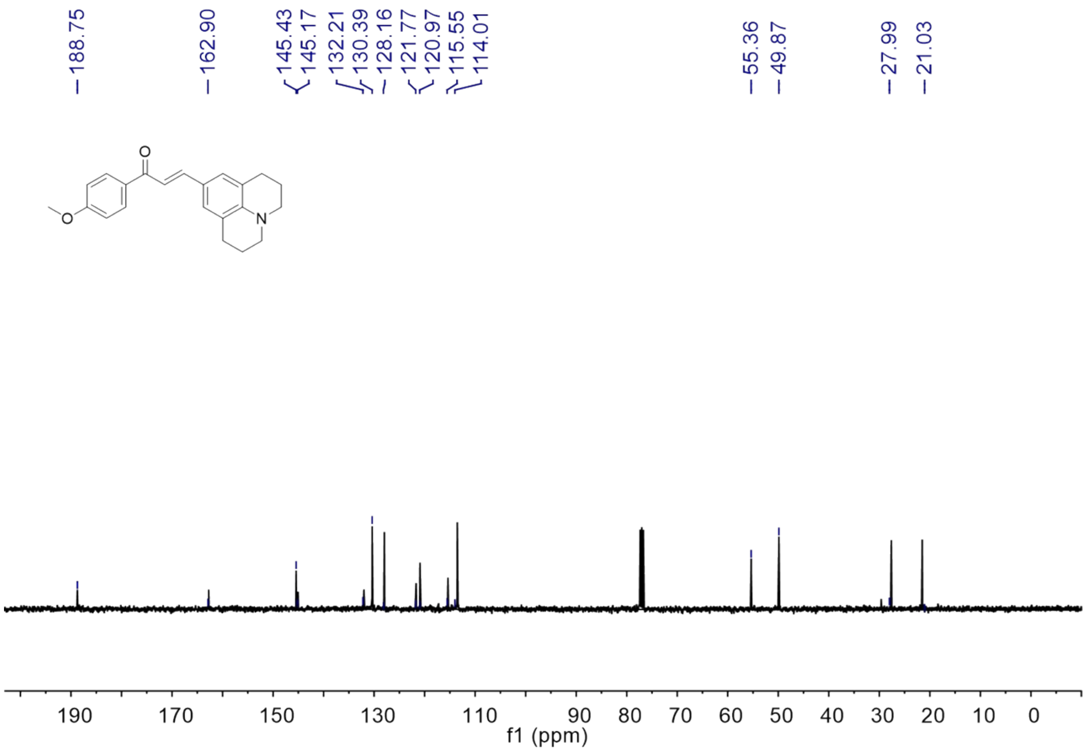

Figure S46. ^{13}C NMR spectrum (100 MHz, $CDCl_3$, 298 K) of **4a**.

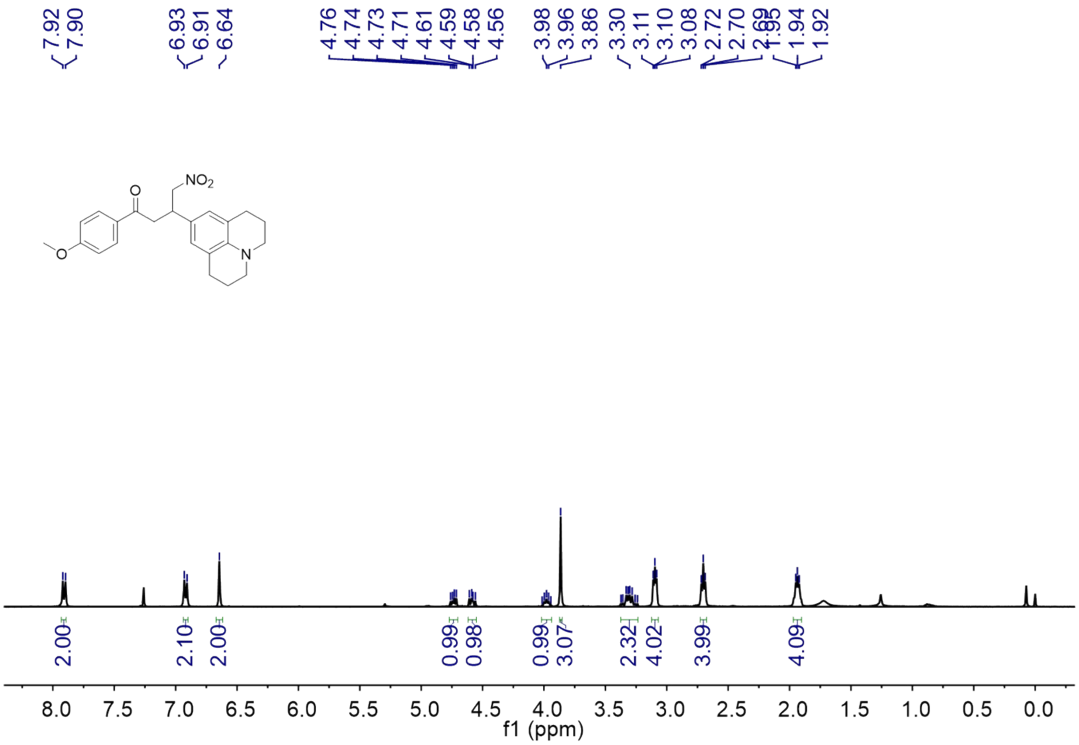

Figure S47. ^{1}H NMR spectrum (400 MHz, $CDCl_3$, 298 K) of **5a**.

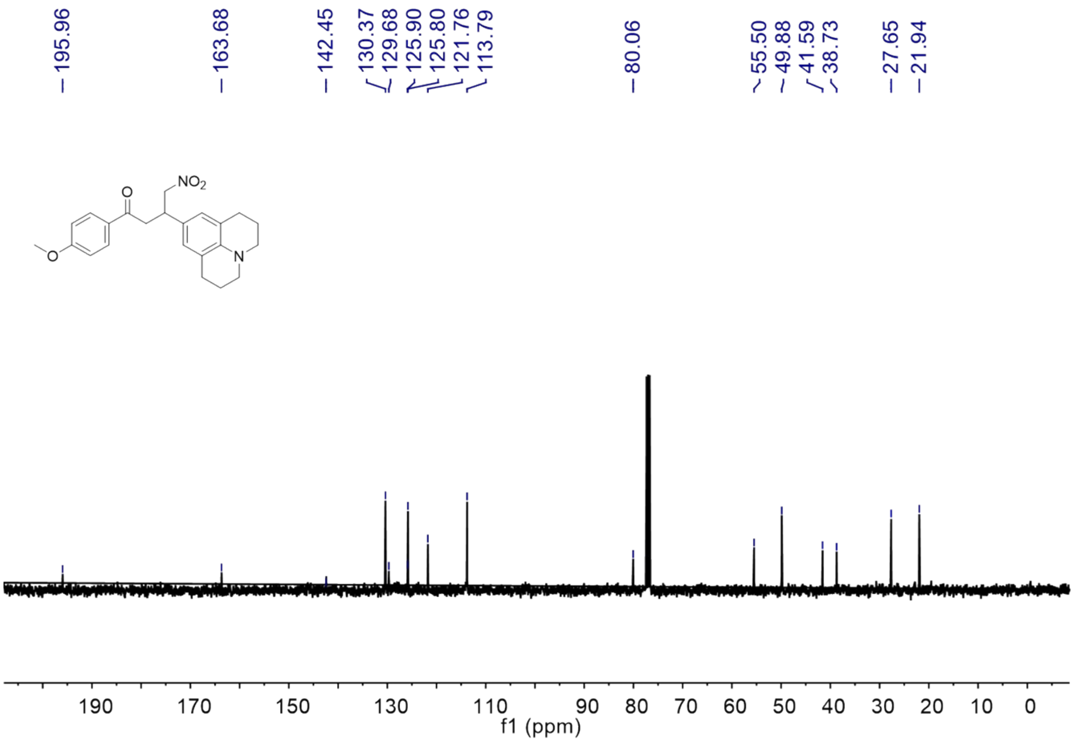

Figure S48. ^{13}C NMR spectrum (100 MHz, $CDCl_3$, 298 K) of **5a**.

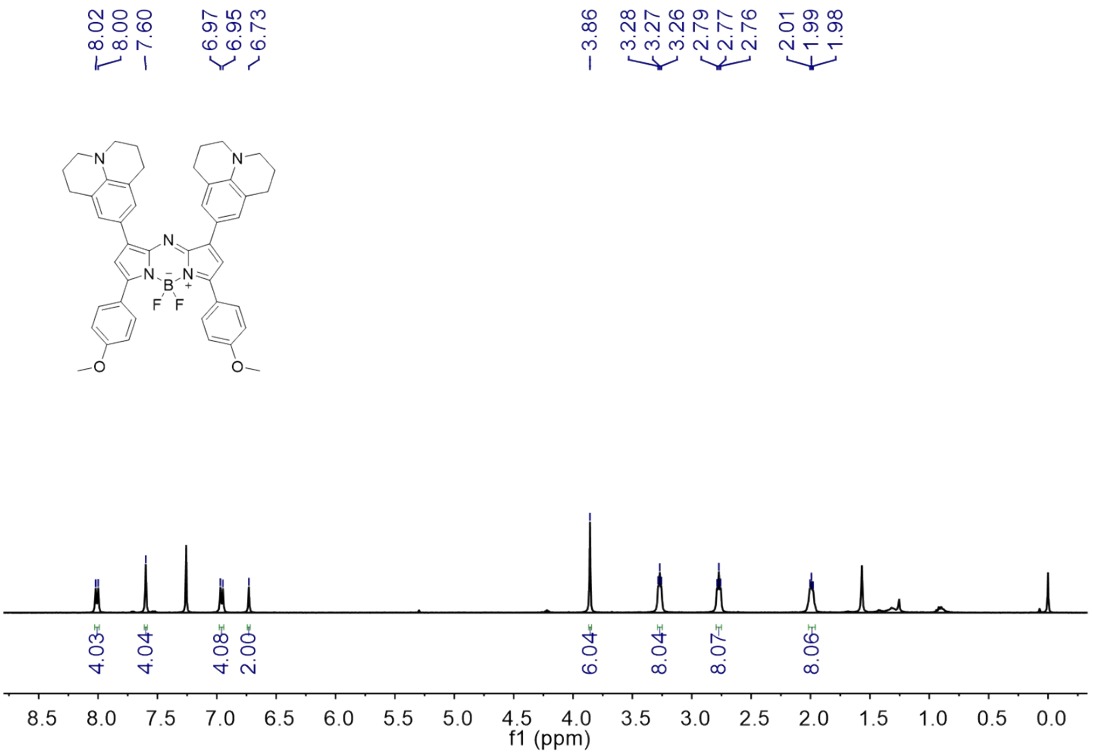

Figure S49. ^{1}H NMR spectrum (400 MHz, $CDCl_3$, 298 K) of **6a**.

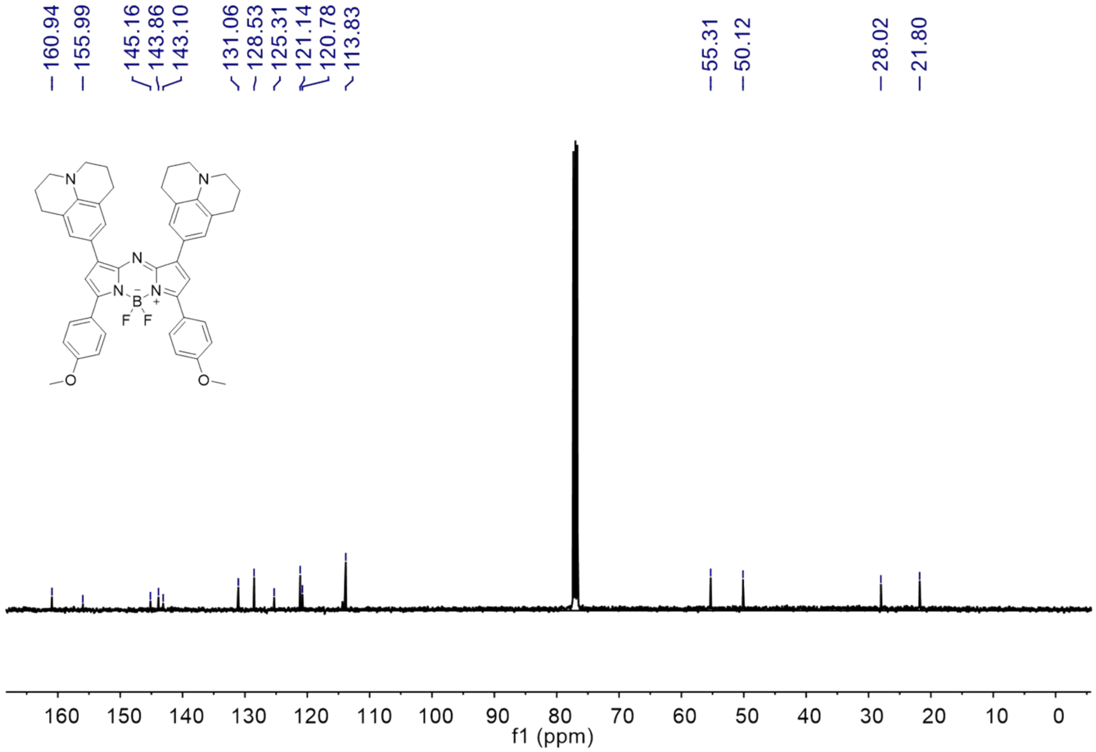

Figure S50. ^{13}C NMR spectrum (100 MHz, $CDCl_3$, 298 K) of **6a**.

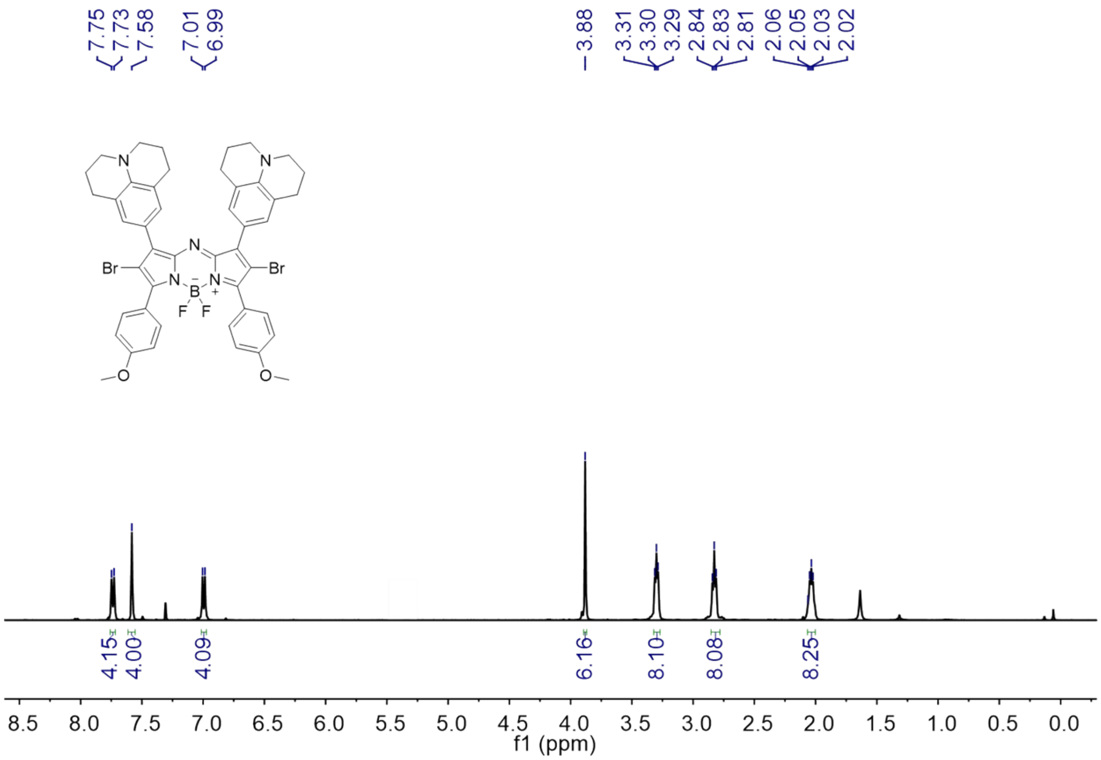

Figure S51. ^{1}H NMR spectrum (400 MHz, $CDCl_3$, 298 K) of **7a**.

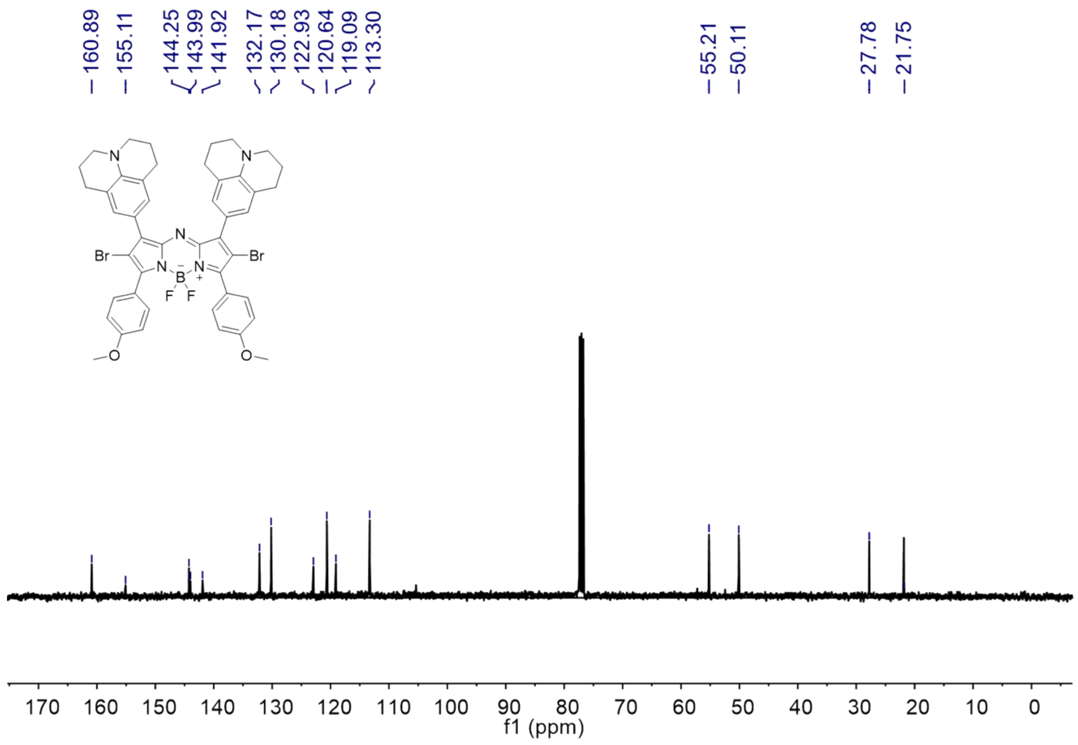

Figure S52. ^{13}C NMR spectrum (100 MHz, $CDCl_3$, 298 K) of **7a**.

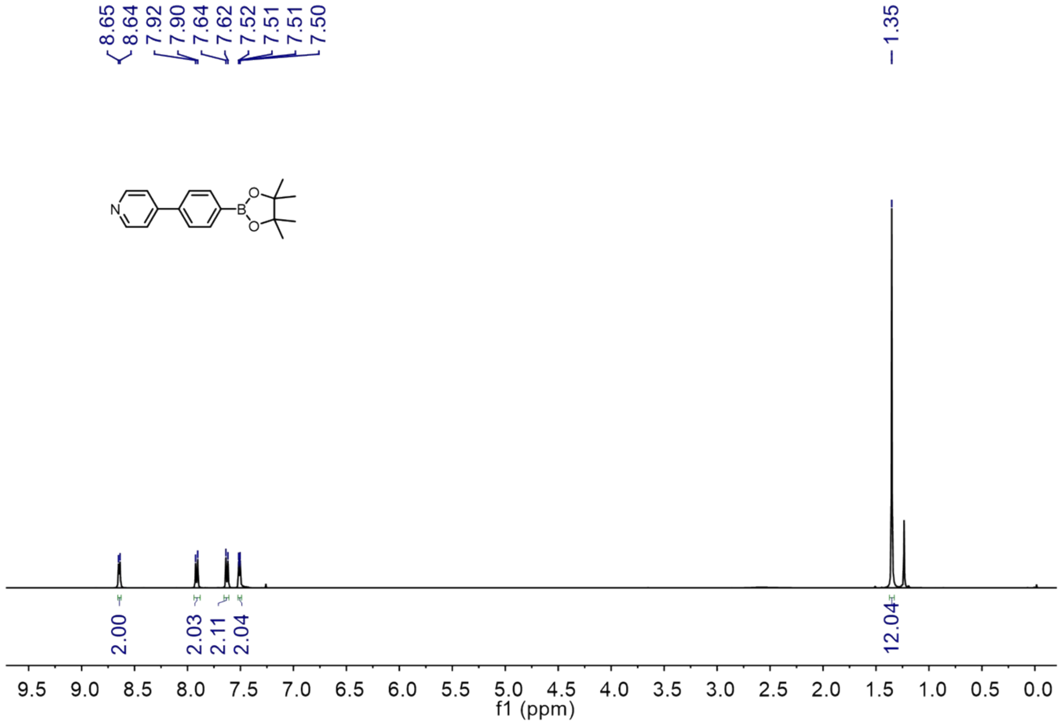

Figure S53. ^{1}H NMR spectrum (100 MHz, $CDCl_3$, 298 K) of **8a**.

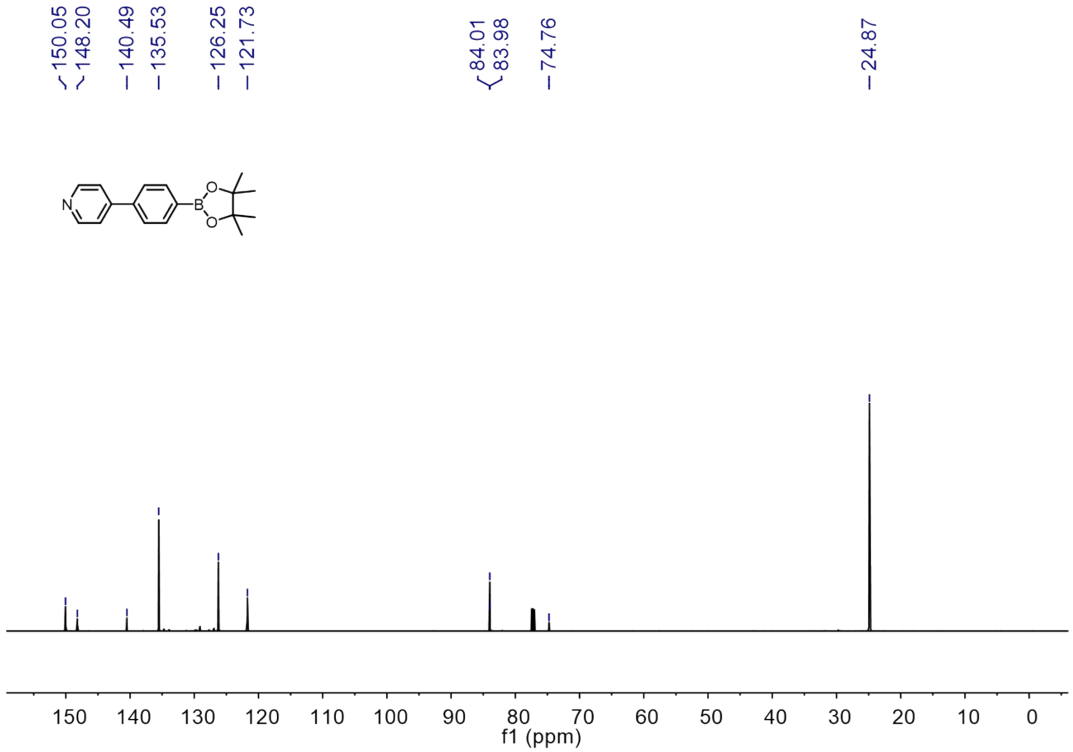

Figure S54. ^{13}C NMR spectrum (100 MHz, $CDCl_3$, 298 K) of **8a**.

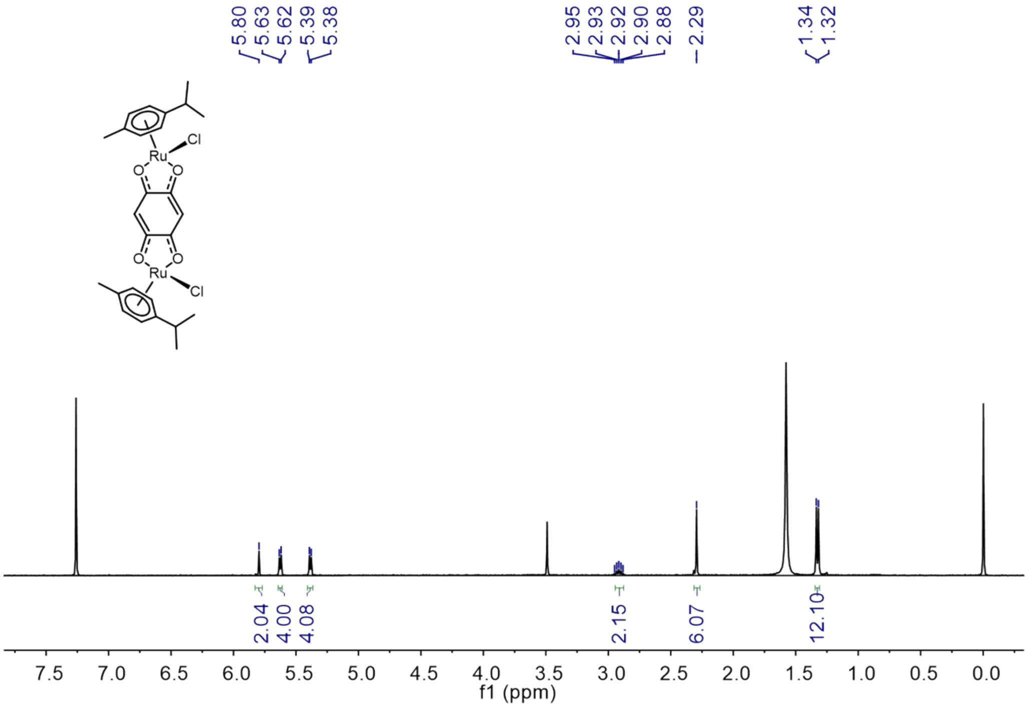

Figure S55. ^{1}H NMR spectrum (400 MHz, $CDCl_3$, 298 K) of **3b**.

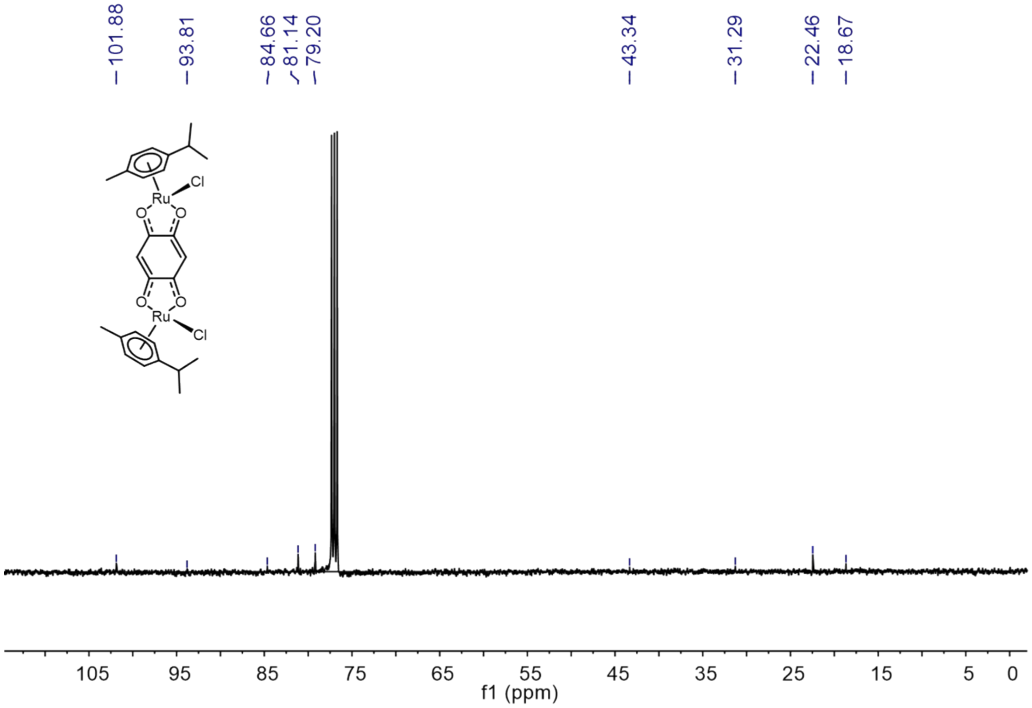

Figure S56. ^{13}C NMR spectrum (100 MHz, $CDCl_3$, 298 K) of **3b**.

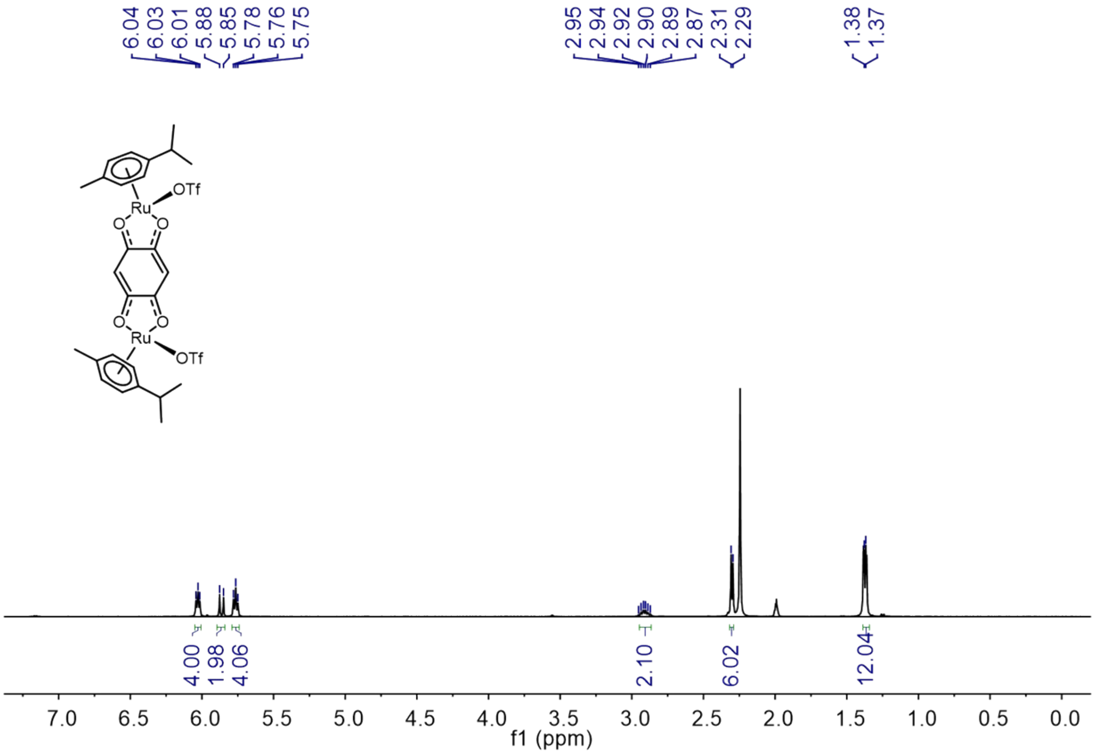

Figure S57. ^{1}H NMR spectrum (400 MHz, CD_3CN, 298 K) of **A1**.

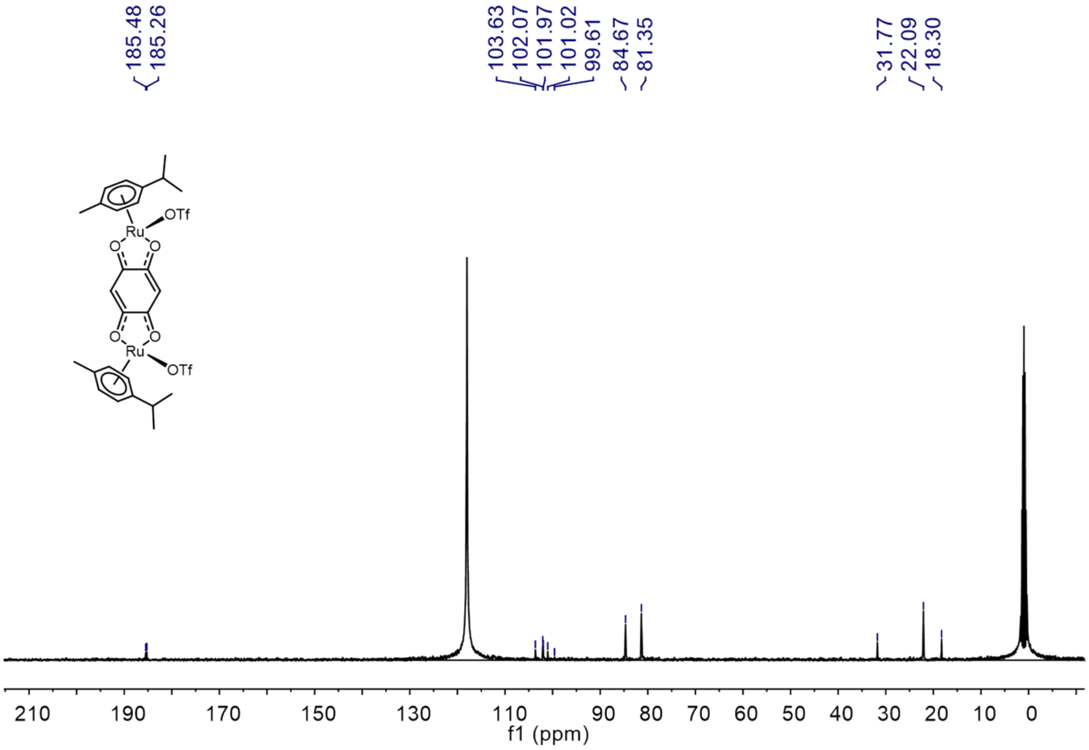

Figure S58. ^{13}C NMR spectrum (100 MHz, CD_3CN, 298 K) of **A1**.

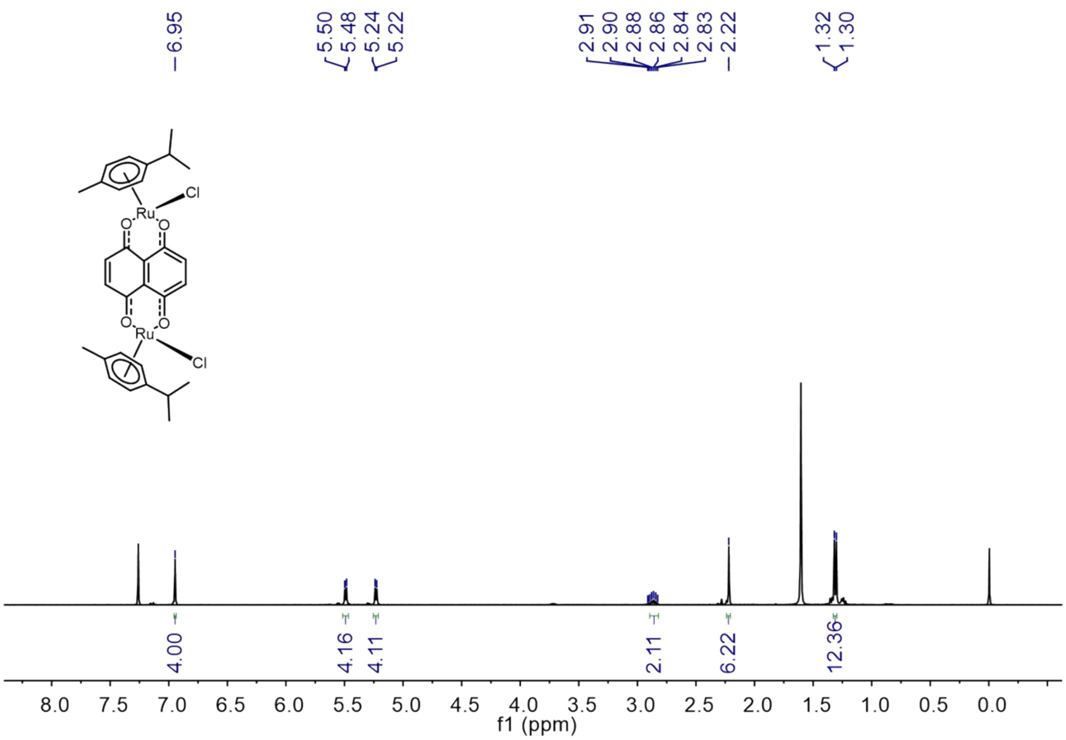

Figure S59. ^{1}H NMR spectrum (400 MHz, $CDCl_3$, 298 K) of **5b**.

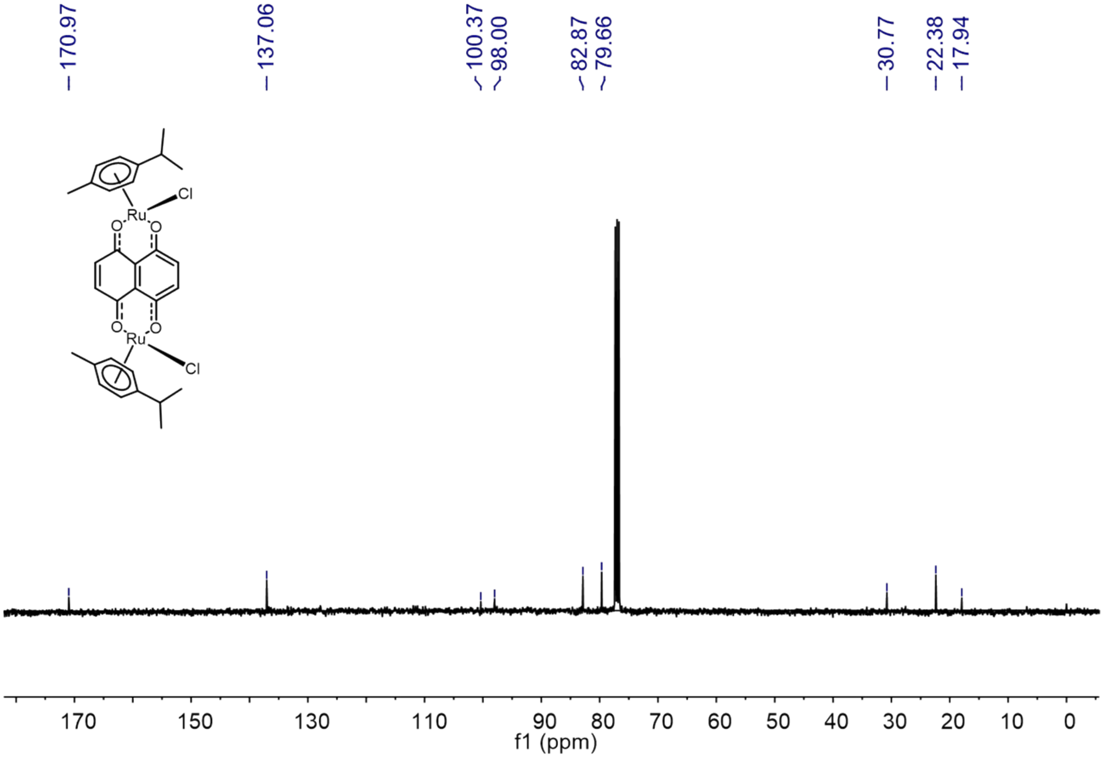

Figure S60. ^{13}C NMR spectrum (100 MHz, $CDCl_3$, 298 K) of **5b**.

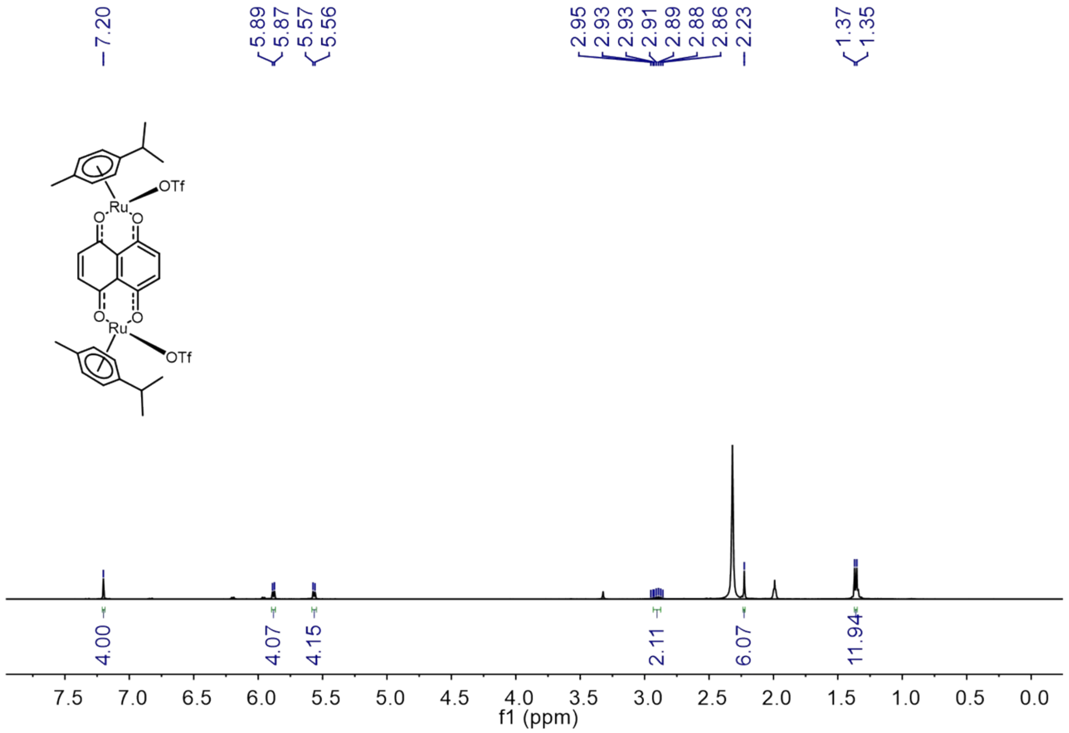

Figure S61. ^{1}H NMR spectrum (400 MHz, CD_3CN, 298 K) of **A2**.

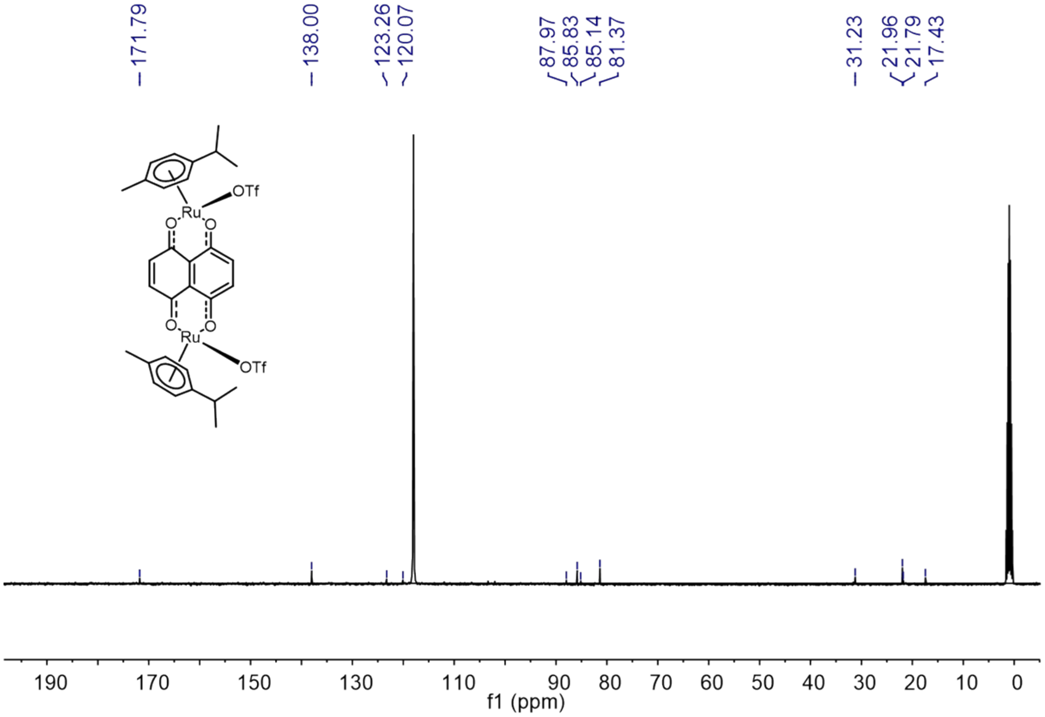

Figure S62. ^{13}C NMR spectrum (100 MHz, CD_3CN, 298 K) of A**2**.

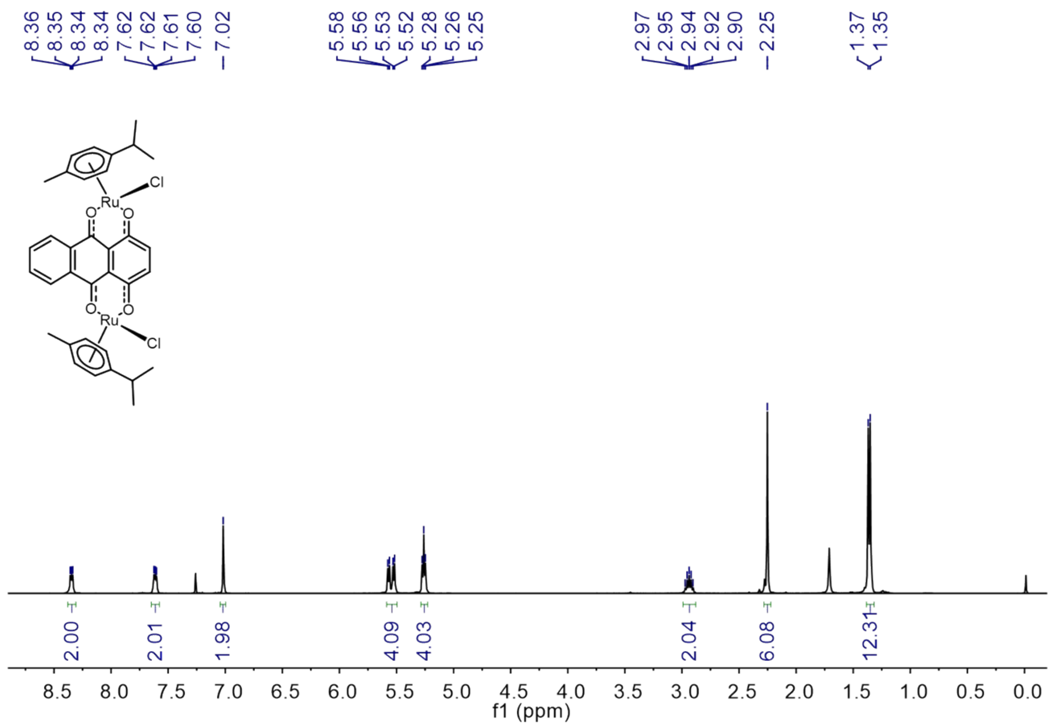

Figure S63. ^{1}H NMR spectrum (400 MHz, $CDCl_3$, 298 K) of **7b**.

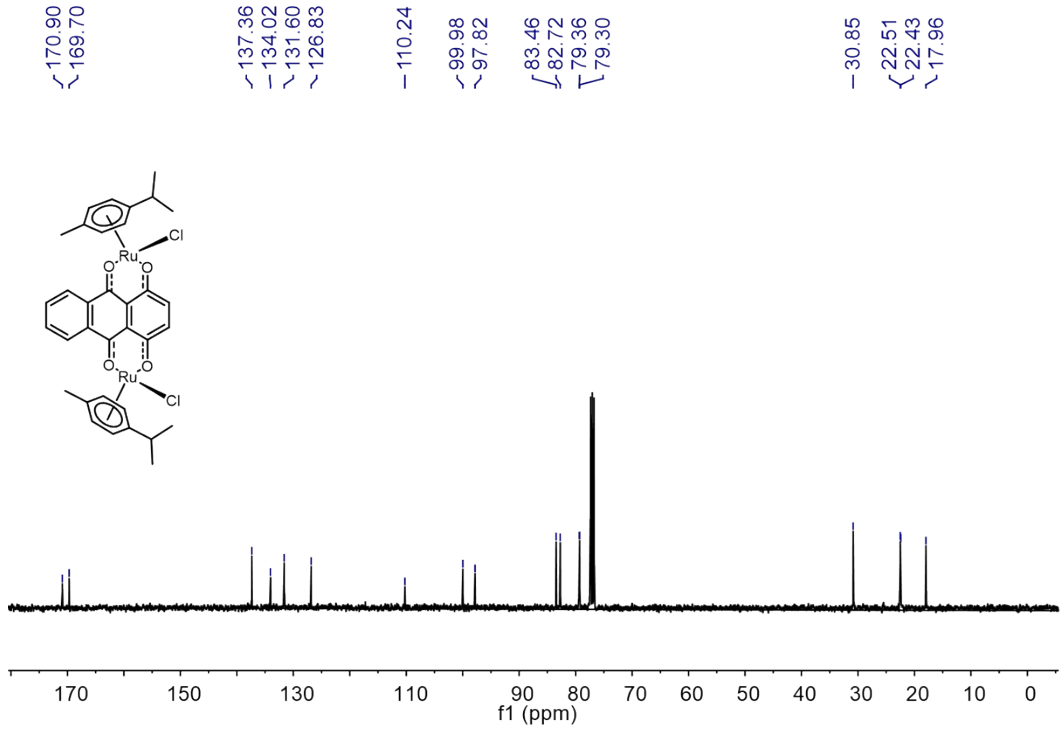

Figure S64. ^{13}C NMR spectrum (100 MHz, $CDCl_3$, 298 K) of **7b**.

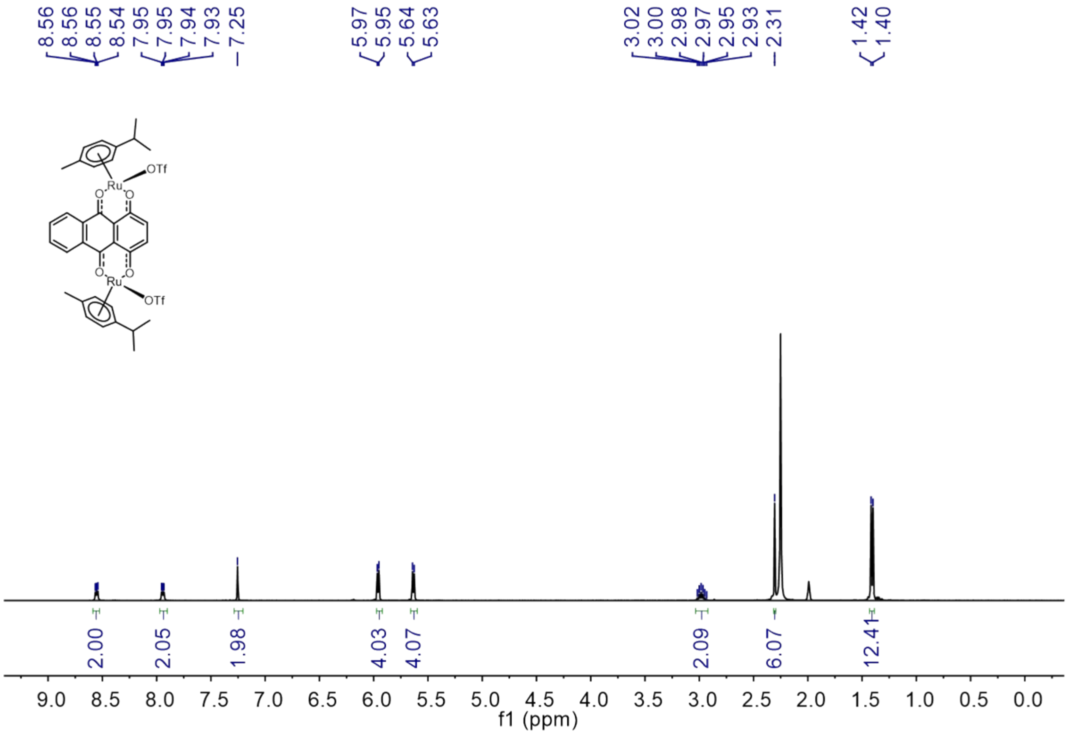

Figure S65. ^{1}H NMR spectrum (400 MHz, CD_3CN, 298 K) of **A3**.

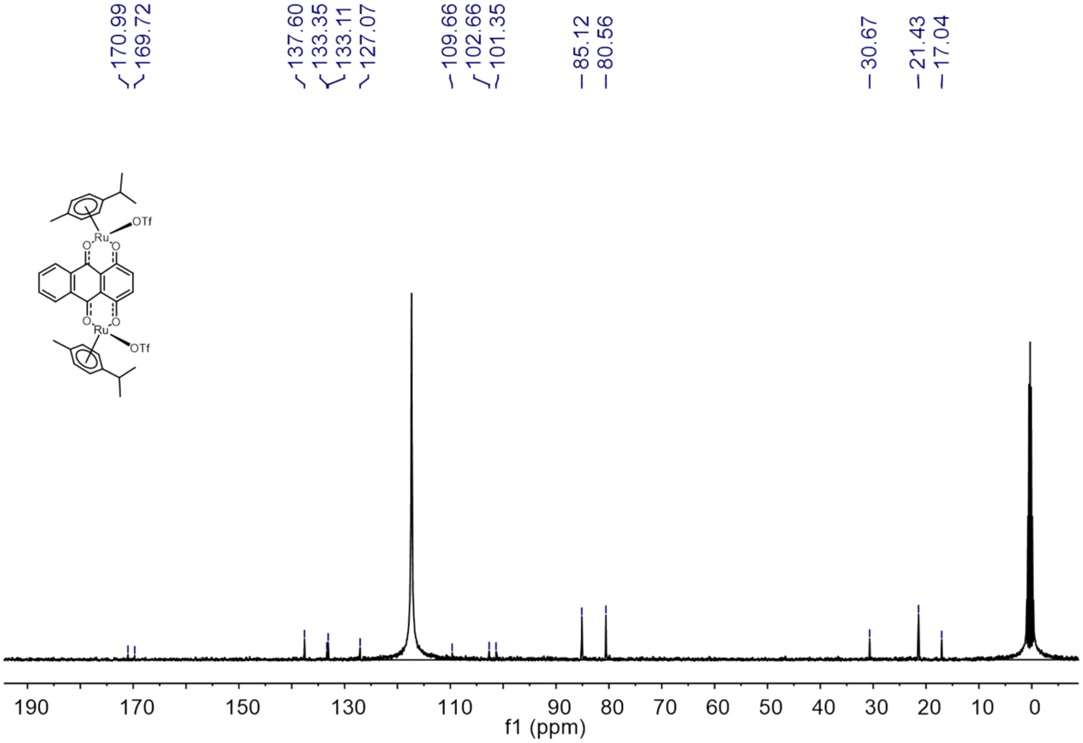

Figure S66. ^{13}C NMR spectrum (100 MHz, CD_3CN, 298 K) of **A3**.

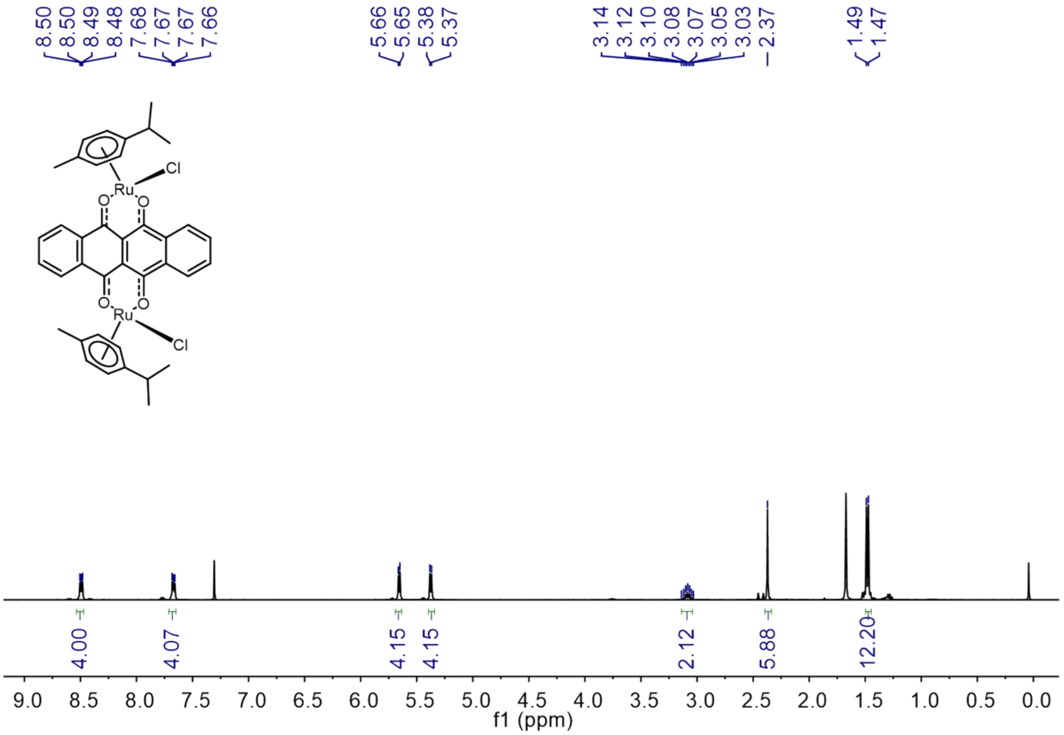

Figure S67. ^{1}H NMR spectrum (400 MHz, $CDCl_3$, 298 K) of **9b**.

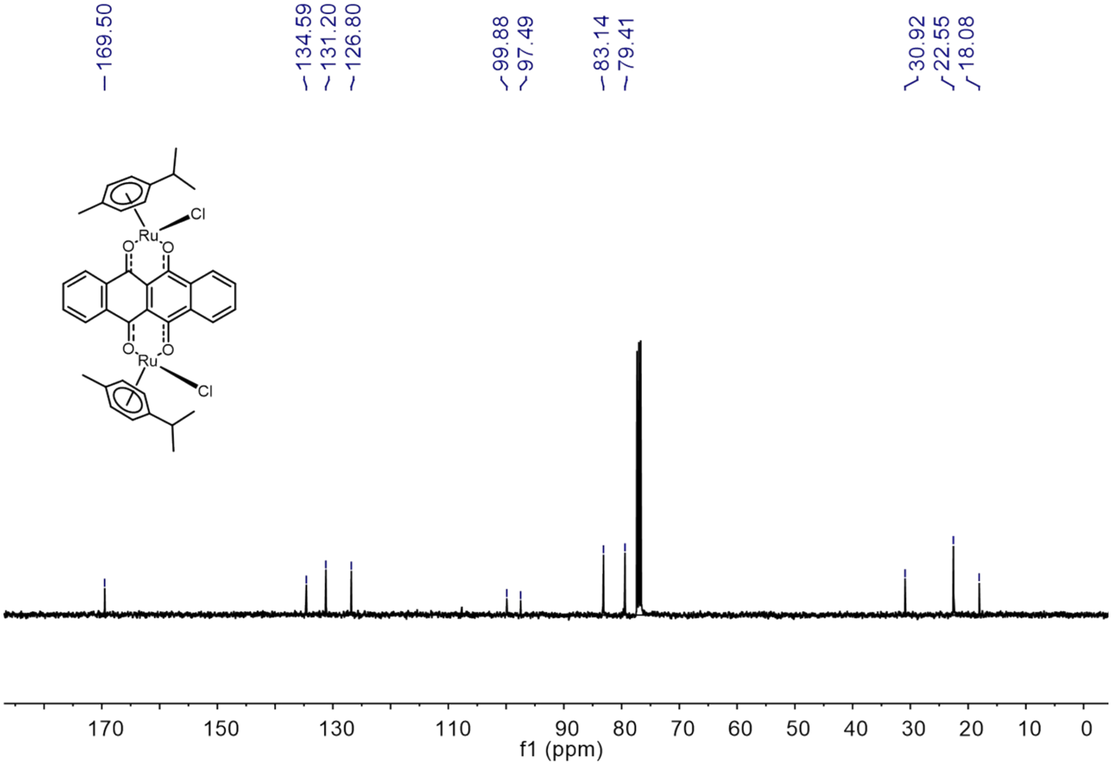

Figure S68. ^{13}C NMR spectrum (100 MHz, $CDCl_3$, 298 K) of **9b**.

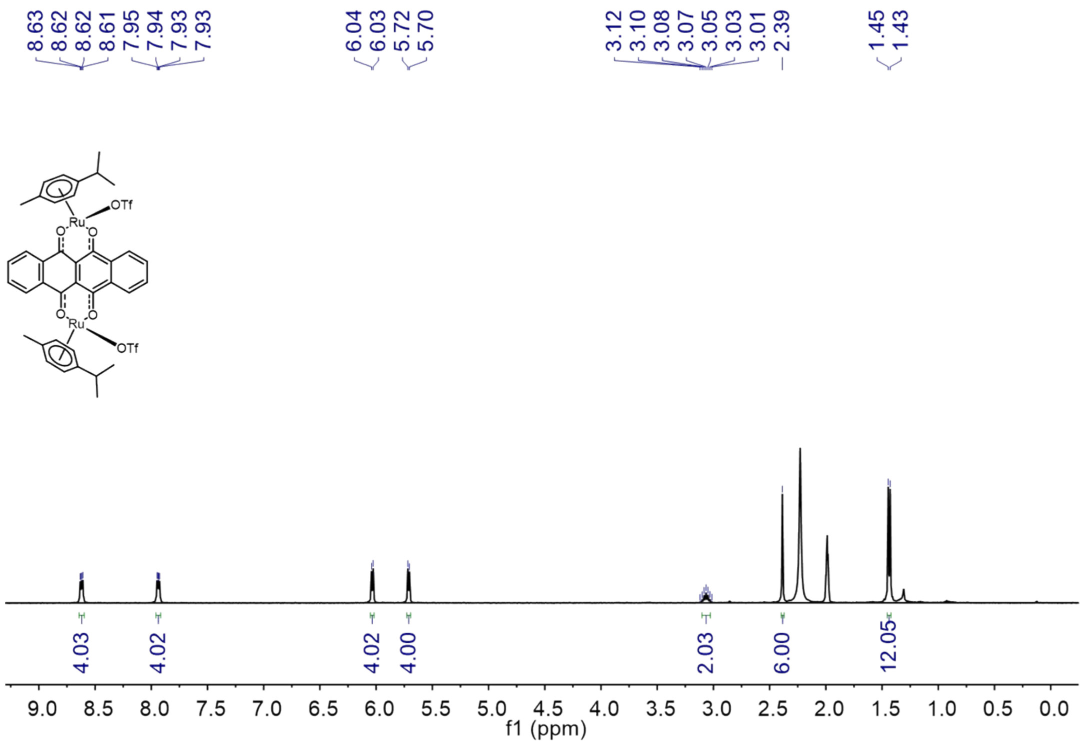

Figure S69. ^{1}H NMR spectrum (400 MHz, CD_3CN, 298 K) of **A4**.

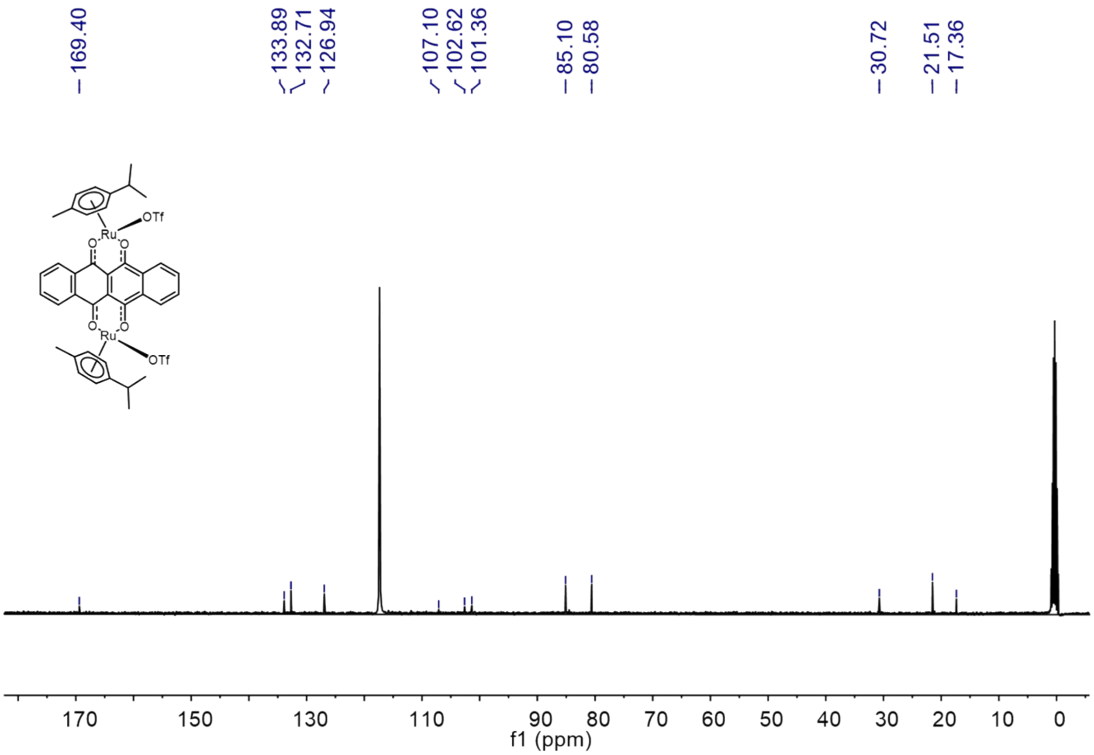

Figure S70. ^{13}C NMR spectrum (100 MHz, CD_3CN, 298 K) of **A4**.

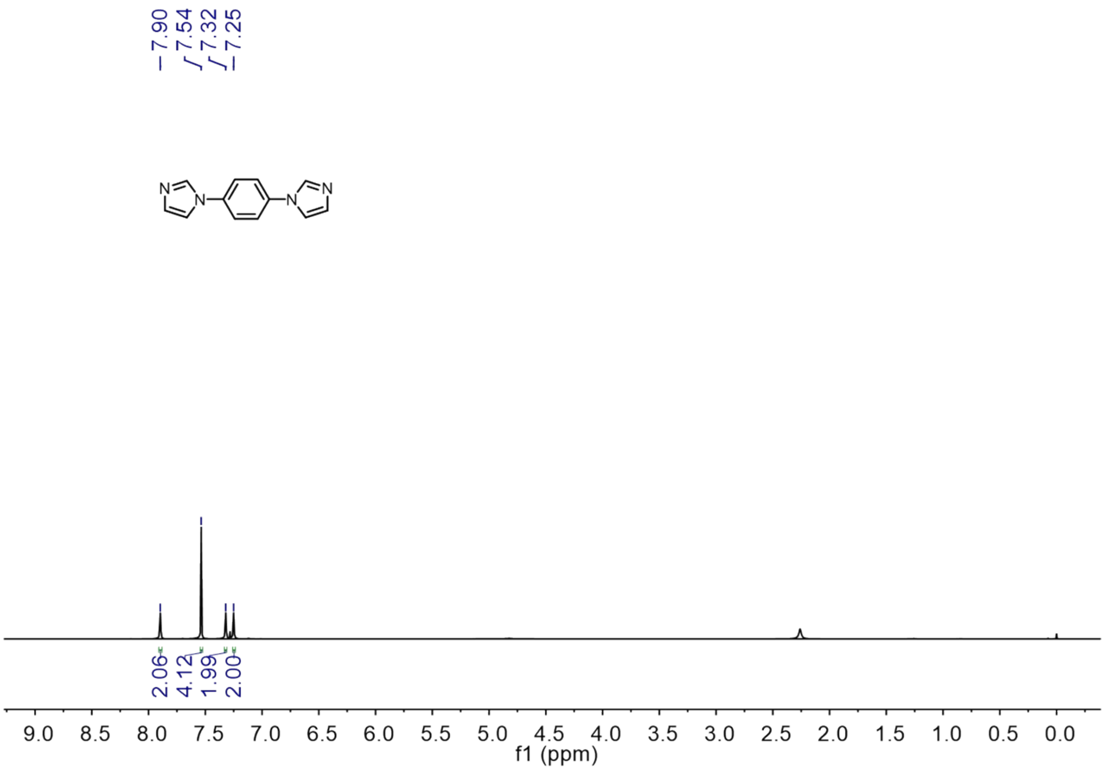

Figure S71. ^{1}H NMR spectrum (400 MHz, $CDCl_3$, 298 K) of **M**.

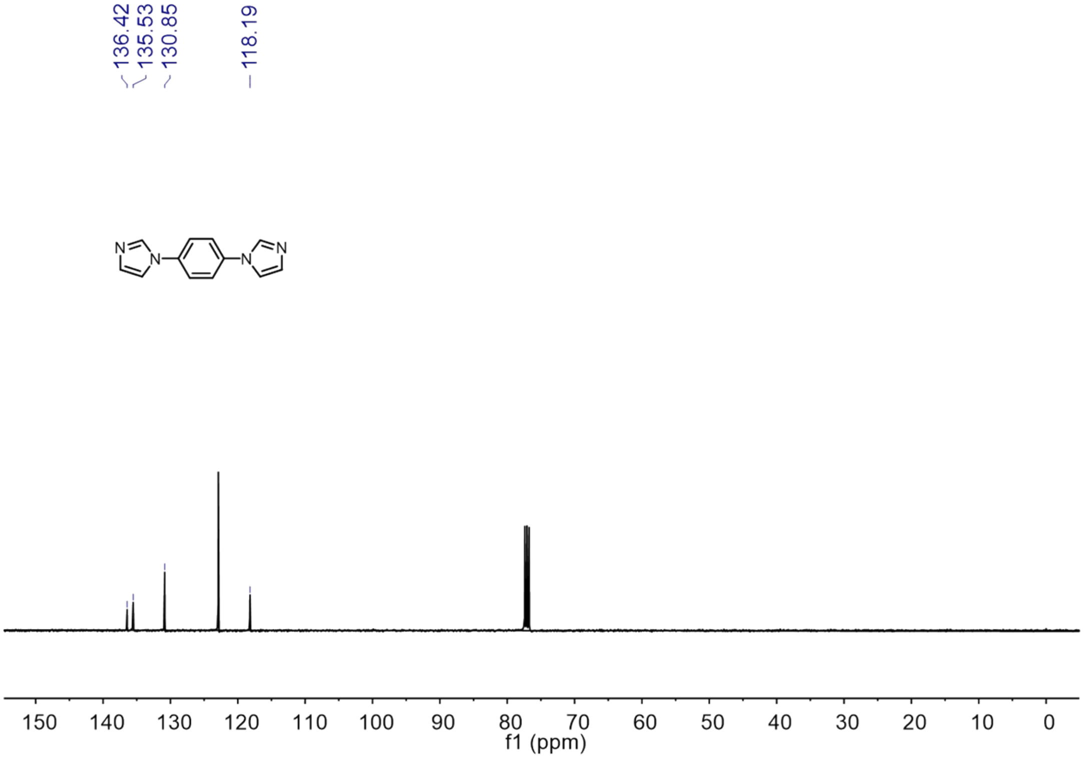

Figure S72. ^{13}C NMR spectrum (100 MHz, $CDCl_3$, 298 K) of **M**.

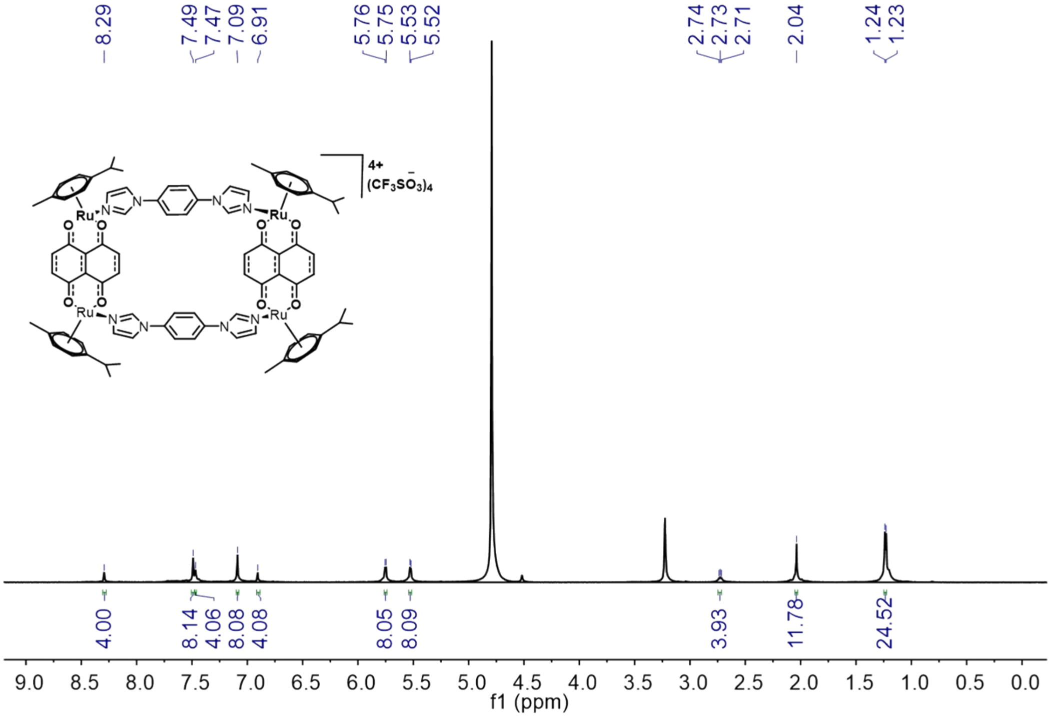

Figure S73. ^{1}H NMR spectrum (400 MHz, CD_3OD, 298 K) of **Ru-M**.

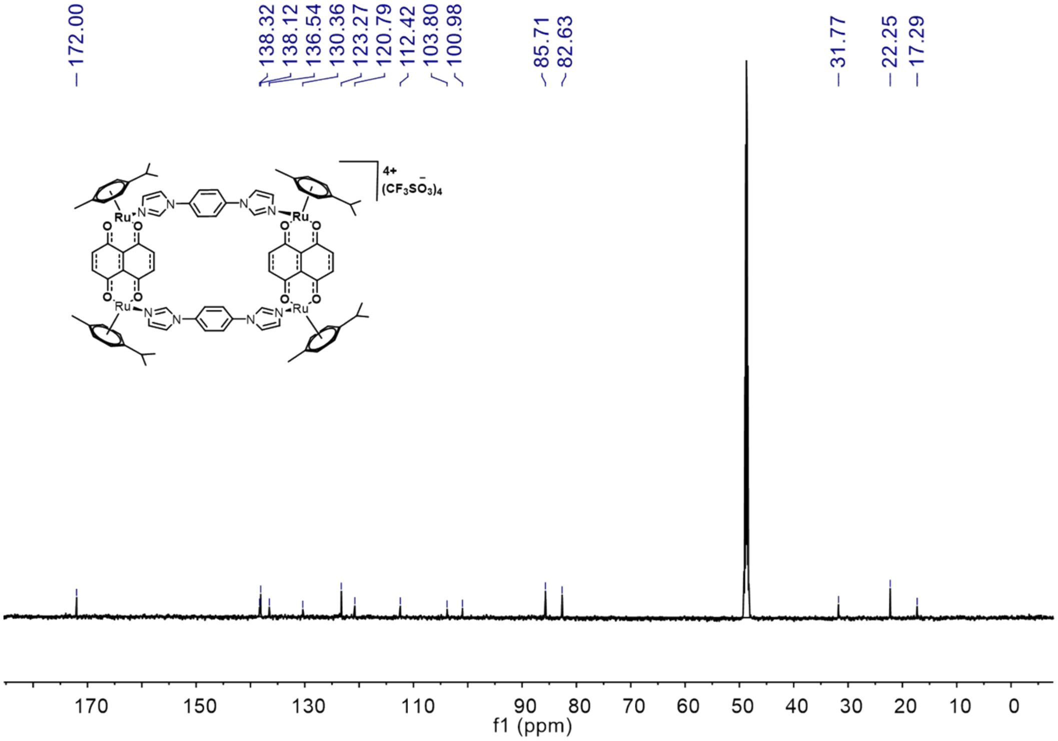

Figure S74. ^{13}C NMR spectrum (100 MHz, CD_3OD, 298 K) of **Ru-M**.

Supplementary References

S1 Bai, L.; Sun, P.; Liu, Y.; Zhang, H.; Hu, W.; Zhang, W.; Liu, Z.; Fan, Q.; Li, L.; Huang, W. Novel aza-BODIPY based small molecular NIR-II fluorophores for in vivo imaging. *Chem. Commun.* **2019**, *55*, 10920-10923.

S2 Adeyemo, A. A.; Shettar, A.; Bhat, I. A.; Kondaiah, P.; Mukherjee, P. S. Coordination-driven self-assembly of ruthenium (II) architectures: synthesis, characterization and cytotoxicity studies. *Dalton. Trans*. **2018**, 47, 8466-8475.

S3. Zhao, Y.; Zhang, L.; Li, X.; Shi, Y.; Ding, R.; Teng, M.; Zhang, P.; Cao, C.; Stang, P. J. Self-assembled ruthenium (II) metallacycles and metallacages with imidazole-based ligands and their in vitro anticancer activity. *Proc. Natl. Acad. Sci. U S A.* **2019**, 116, 4090-4098.

S4. Su, S. J.; Tanaka, D.; Li, Y.; Sasabe, H.; Takeda, T.; Kido, J. Novel four-pyridylbenzene-armed biphenyls as electron-transport materials for phosphorescent OLED. *Org. lett.* **2008**, *10*, 941-944.

S5. Gomes, A.; Fernandes, E.; Lima, J. L. Fluorescence probes used for detection of reactive oxygen species. *Biochem.Biophys. Methods* **2005**, *65*, 45-80.
